# Genetically identification of endometriosis and cancers risk in women through a two-sample Mendelian randomization study

**DOI:** 10.1038/s41598-024-58950-7

**Published:** 2024-04-10

**Authors:** Liqi Zhang, Huaqing Yan

**Affiliations:** 1 Department of Reproductive Medicine, The First Affiliated Hospital of Ningbo University (Ningbo First Hospital), Ningbo University, Ningbo, 315000 Zhejiang People’s Republic of China; 2 Department of Urology, Ningbo Medical Center Lihuili Hospital, Ningbo University, Ningbo, 315000 Zhejiang People’s Republic of China

**Keywords:** Causality, Mendelian randomization, Endometriosis, Cancer, Ovarian cancer, Cancer, Pathogenesis

## Abstract

Endometriosis is a prevalent and chronic inflammatory gynecologic disorder affecting approximately 6–10% of women globally, and has been associated with an increased risk of cancer. Nevertheless, previous studies have been hindered by methodological limitations that compromise the validity and robustness of their findings. In this study we conducted a comprehensive two-sample Mendelian randomization analysis to explore the genetically driven causal relationship between endometriosis and the risk of cancer. We conducted the analysis via the inverse variance weighted method, MR Egger method, and weighted median method utilizing publicly available genome-wide association study summary statistics. Furthermore, we implemented additional sensitivity analyses to assess the robustness and validity of the causal associations identified. We found strong evidence of a significant causal effect of endometriosis on a higher risk of ovarian cancer via inverse-variance weighted method (OR = 1.19, 95% CI 1.11–1.29, *p* < 0.0001), MR-Egger regression, and weighted median methodologies. Remarkably, our findings revealed a significant association between endometriosis and an increased risk of clear cell ovarian cancer (OR = 2.04, 95% CI 1.66–2.51, *p* < 0.0001) and endometrioid ovarian cancer (OR = 1.45, 95% CI 1.27–1.65, *p* < 0.0001). No association between endometriosis and other types of cancer was observed. We uncovered a causal relationship between endometriosis and an elevated risk of ovarian cancer, particularly clear cell ovarian cancer and endometrioid ovarian cancer. No significant associations between endometriosis and other types of cancer could be identified.

## Introduction

Endometriosis is an inflammatory condition with lesions made up of endometrial-like tissue outside the uterus, including ovaries, the perineum, the lung, or even the central nervous system^1^. It affects approximately 6–10% of women worldwide and causes symptoms such as dysmenorrhea, pelvic pain, and infertility, significantly impairing the quality of life for women^2,3^. Endometriosis has the potential to involve multiple organ systems and its symptoms are often chronic, resulting in a significant impact on work productivity, social life, intimate relationships, and mental health, as well as substantial societal costs. Additionally, endometriosis can affect fertility by modifying the peritoneal environment or distorting the pelvic anatomy, with approximately 30% of patients experiencing difficulties conceiving^4^. Moreover, several observational studies reported that women diagnosed with endometriosis through surgery have a significantly increased risk of developing various types of cancer when compared to the general population^5–9^.

It is of paramount importance to quantify the cancer risk associated with endometriosis. This area of study holds significant public health implications for women regarding cancer screening and prevention, as well as for clinicians in the ongoing management of patients diagnosed with endometriosis. Considering the current limited understanding of endometriosis, a thorough exploration and understanding of its correlation with cancer will notably enhance our grasp of endometriosis pathophysiology, thus propelling the advancement of endometriosis treatment. However, whether endometriosis is a risk factor for cancers remains unclear. A recent meta-analysis reported that a higher risk of ovarian and thyroid cancer has been observed in the context of endometriosis, while the association with breast cancer appears to be minimal^10^. Meanwhile, another meta-analysis showed an increased risk of endometrial and thyroid cancer, an inverse association with cervical cancer, and no association with breast cancer^11^. While numerous studies have explored the relationship between measures of endometriosis and cancer risk, the causal role of endometriosis in cancers remains uncertain.

Mendelian randomization (MR) is a method that utilizes genetic variation arising from meiosis to investigate the causal relationship between exposure and complex outcomes. MR analysis relies on integrating summary data from genome-wide association studies (GWAS)^12^. A genetic variable is deemed valid in MR analysis if it satisfies the following three assumptions: (i) the genetic variants are significantly associated with exposure, (ii) the genetic variants are independent of confounders between exposure and outcomes, and (iii) the genetic variants only influence the outcome through exposure^13^. Two-sample MR analysis refers to an MR analysis that includes a pair of exposures and outcomes from two different datasets^14^. For instance, Zheng et al. utilized data from UK Biobank (UKB) and FinnGen to indicate that obesity might increase the risk of diabetic retinopathy^15^. With the rapid development of large-scale GWAS, numerous MR studies have emerged, exploring the potential causal relationship between exposure and cancers. For example, type 2 diabetes mellitus (T2DM) has been associated with several cancers^16^.

Our investigation leveraged extensive GWAS data to explore the impact of endometriosis on women’s cancer risk through a two-sample MR analysis. This study significantly contributes to elucidating the genetic impact of endometriosis on women’s risk of cancer.

## Methods

### Genetic instruments

**Figure 1:**
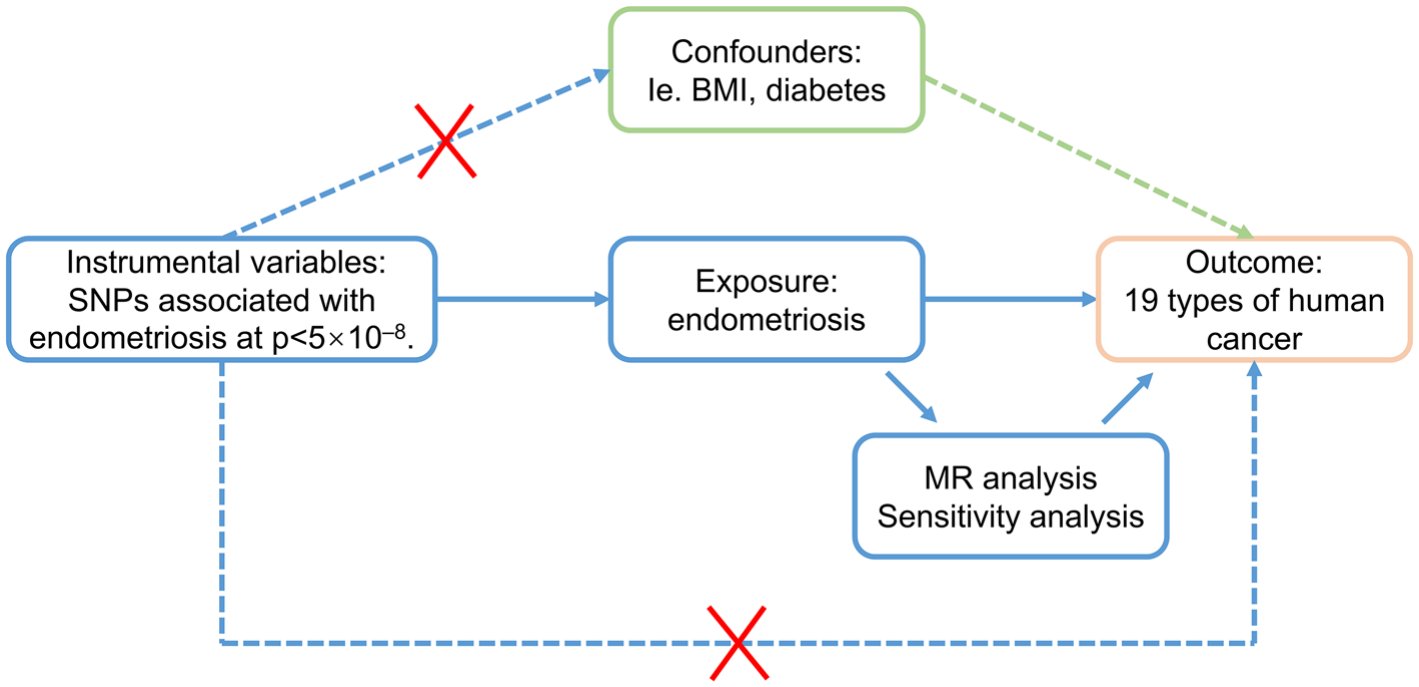
Study design of Mendelian randomization between endometriosis and 19 types of cancer. The solid blue lines depict the relationship between the instrumental variables and exposure, as well as the association between exposure and outcome through MR analysis. Dashed lines with a cross signify that the association satisfies the two fundamental assumptions of Mendelian randomization: (i) the genetic variants are unrelated to confounding factors between exposure and outcomes, and (ii) the genetic variants solely influence the outcome through exposure.

**Figure 2:**
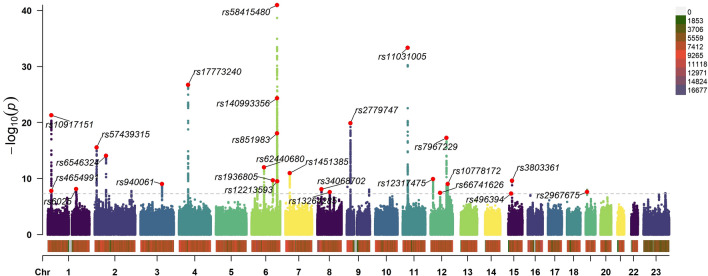
The Manhattan plot illustrates the association of SNPs with endometriosis. The X-axis is sequentially arranged to represent chromosomes 1 through 23, with each SNP’s chromosomal position denoted by dots. The Y-axis delineates the association analysis outcomes, expressed as the negative logarithm of the *p*-values (−log10 [*p*-value]), where elevated positions correspond to SNPs exhibiting more robust associations. A grey dashed line parallel to the X-axis signifies the *p*-value threshold at 5 × 10^–8^, with points surpassing this threshold indicating loci of significant genetic association. Red dots denote all SNPs included as instrumental variables in the analysis.

Figure 1 depicts the workflow of our research. The most recent data for endometriosis was obtained from FinnGen which was released May 11, 2023. The data consists of a large-scale GWAS including 15,088 European endometriosis cases and 107,564 controls, respectively. The threshold for selecting single nucleotide polymorphisms (SNPs) was set at a significant level of *P* < 5 × 10^–8^. To ensure independence between SNPs, r^2^ value was calculated to verify the linkage disequilibrium (LD) among them. LD was defined based on SNPs with an r^2^ value greater than 0.001 and a physical distance within 10,000 kb. Among the LD clusters formed, only those with the most significant *p*-values were retained. 27 independent SNPs were generated after linkage disequilibrium elimination (Supplementary Table 2). Next, we conducted a comprehensive search of all single nucleotide polymorphisms (SNPs) in Phenoscanner, a curated database that houses publicly available results from large-scale GWAS encompassing more than 65 billion associations and over 150 million genetic variants^17^. The purpose of this step was to assess whether these SNPs were related to other risk factors at the significant level (*P* < 5 × 10^–8^) that could be potential confounders. We removed rs635634 because it was significantly related to diabetes and ovarian cancer. Then, rs2483211 was removed because it was associated with Whole body fat mass. Finally, we included the remaining 25 endometriosis-associated SNPs (Supplementary Table 3) as instrumental variables in the MR analysis. A Manhattan plot was depicted to illustrate the association of SNPs with endometriosis (Fig. 2). These included SNPs explained approximately 12.3% of the variability in endometriosis. To evaluate the potential risk of weak instrument bias, we employed F tests to ascertain the magnitude of the association in the initial stage regressions between allele score and exposure. We calculated an F statistic of 663, indicating the robustness of the instrument and minimizing the potential for weak instrument bias.

### The data source of human cancers

The summary statistics from the GWAS for cancers in the publicly available databases were retrieved from the IEU open GWAS project (https://gwas.mrcieu.ac.uk/datasets/). To reduce population stratification bias, we only included European cancer cohorts^18^. The details of cancer data was indicated in Supplementary Table 1. Briefly, the GWAS data for ovarian cancer was obtained from Ovarian Cancer Association Consortium, OCAC^19^. The summary data for endometrial cancer, bladder cancer, kidney cancer, and cervical cancer was derived from MRC-IEU^20^. The estimates for the relationship between the SNPs and risk of breast cancer, lung cancer, head and neck cancer, laryngeal cancer, oral cavity cancer, oral and oropharyngeal cancer, oropharyngeal cancer, colorectal cancer, and Melanoma were obtained from UK Biobank. In our final analysis, we included a cohort totaling approximately six million participants, with the smallest tumor cohort comprising 43,751 individuals and the largest encompassing 463,010 individuals, thereby significantly enhancing the robustness of our results^21^.Though we could not totally rule out the possibility of the same or similar individuals existing in these databases, a small number of individuals who overlap between the exposure and outcome studies might not be severe and the result is still convincing^22^.

### Estimation of a causal association between endometriosis and cancer

After harmonizing the exposure and outcome data to ensure that the SNP effects on the same allele were reconciled, we utilized a series of two-sample MR methods, such as inverse-variance weighted (IVW), MR Egger, and weighted median to estimate the association between endometriosis and different types of cancer. The IVW method is considered the most efficient MR method, as it assumes that all genetic variants are valid instrumental variables (IV). However, the weighted median estimator allows for up to half of the SNPs to not be IVs, and this method can evaluate whether SNPs have pleiotropic effects on the outcome^23^. We employed odds ratios (ORs) along with their corresponding 95% confidence intervals (CIs) to elucidate the impact of endometriosis on cancer risk. Statistical significance was determined by a *p*-value of less than 0.05. To further assess the robustness of the causal association, a range of sensitivity analyses were conducted, including the heterogeneity test using Cochran’s Q test (*p* < 0.05 indicates a significant heterogeneity), the pleiotropy test, and MR presso method to analyze the outlier-corrected *p* value. The SNPs with missing data were deleted. All analyses described were conducted using R software (version 4.3.1). The TwoSampleMR R package (version 0.5.7) was employed for the MR analysis. The online web tool Sangerbox was used to visualize the results of forest plots^24^. The mRnd website (https://shiny.cnsgenomics.com/mRnd/) was used to calculate the statistical power^25^.

## Results

### The causal effect of endometriosis on ovarian cancer

Using the 25 endometriosis-associated SNPs (Supplementary Table 3), we found strong evidence of a significant causal effect of endometriosis on a higher risk of ovarian cancer via inverse-variance weighted method (OR = 1.19, 95% CI 1.11–1.29, *p* < 0.0001). Meanwhile, similar risk estimations were obtained through the utilization of MR-Egger regression (OR = 1.38, 95% CI 1.12–1.70, *p* < 0.01) and weighted median methodologies (OR = 1.18, 95% CI 1.08–1.30, *p* < 0.001). However, a slight heterogeneity was observed with a Cochran Q-test derived *p*-value of 0.045 for IVW and a *p*-value of 0.075 for MR-Egger. No significant evidence of pleiotropy was observed, with a *p*-value of 0.15, indicating that there were no genetic effects beyond the intended exposure of interest.

**Figure 3:**
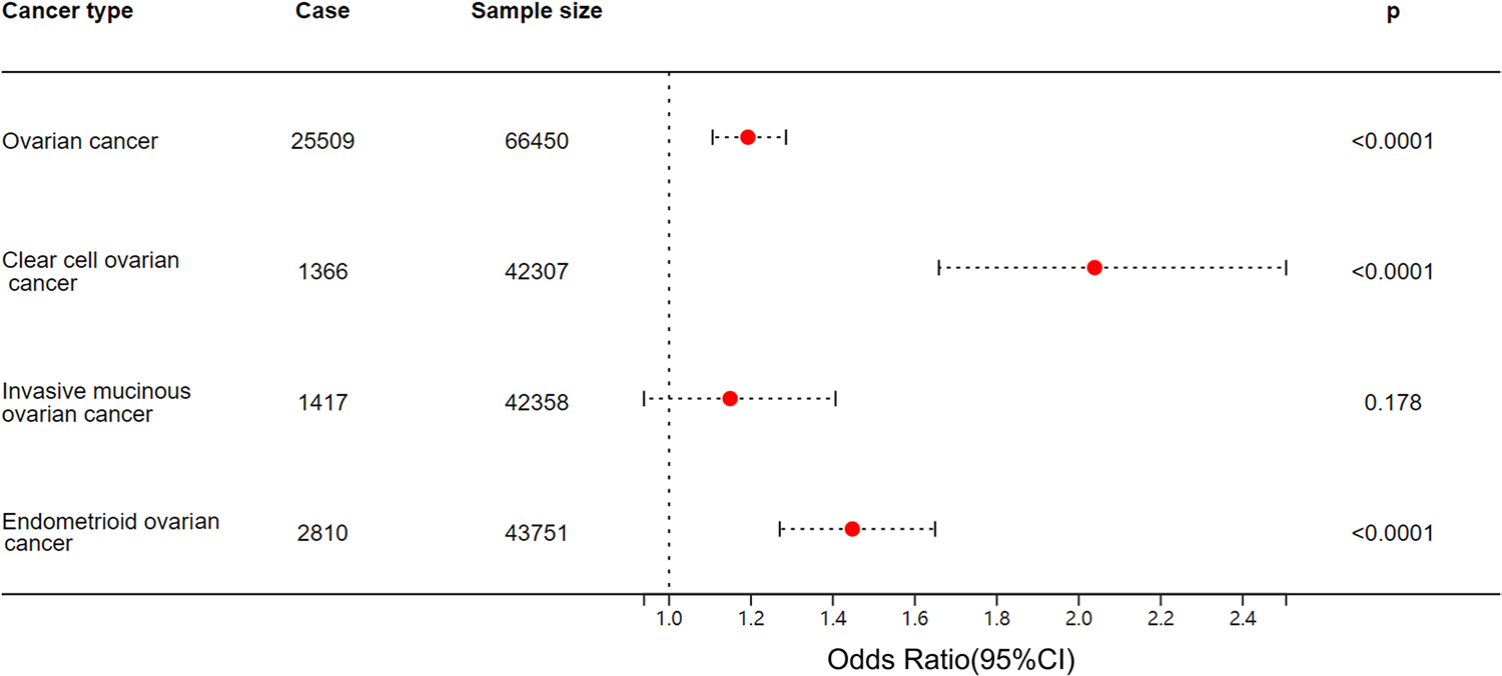
MR analysis revealed the causal influence of endometriosis on different histological subtypes of ovarian cancer. There were significant and robust associations between endometriosis and an increased risk of ovarian cancer, clear cell ovarian cancer, and endometrioid ovarian cancer. Case, the number of patient who has been diagnosed with a specific type of cancer in the cohort; Sample size, the number of individuals involved in the study.

Given the well-established association between endometriosis and an elevated risk of ovarian cancer, we conducted a comprehensive analysis to explore the causal impact of endometriosis on specific histological subtypes of ovarian cancer, namely clear cell ovarian cancer, invasive mucinous ovarian cancer, and endometrioid ovarian cancer via inverse-variance weighted method. Remarkably, our findings revealed a significant association between endometriosis and an increased risk of clear cell ovarian cancer (OR = 2.04, 95% CI 1.66–2.51, *p* < 0.0001). Similarly, a relationship between endometriosis and a higher risk of endometrioid ovarian cancer was found (OR = 1.45, 95% CI 1.27–1.65, *p* < 0.0001). However, no statistically significant association was observed between endometriosis and invasive mucinous ovarian cancer (OR = 1.15, 95% CI 0.94–1.41, *p* = 0.18). Figure 3 presents the forest plot illustrating the causal influence of endometriosis on ovarian cancer.

### The causal effect of endometriosis on other cancers

**Figure 4:**
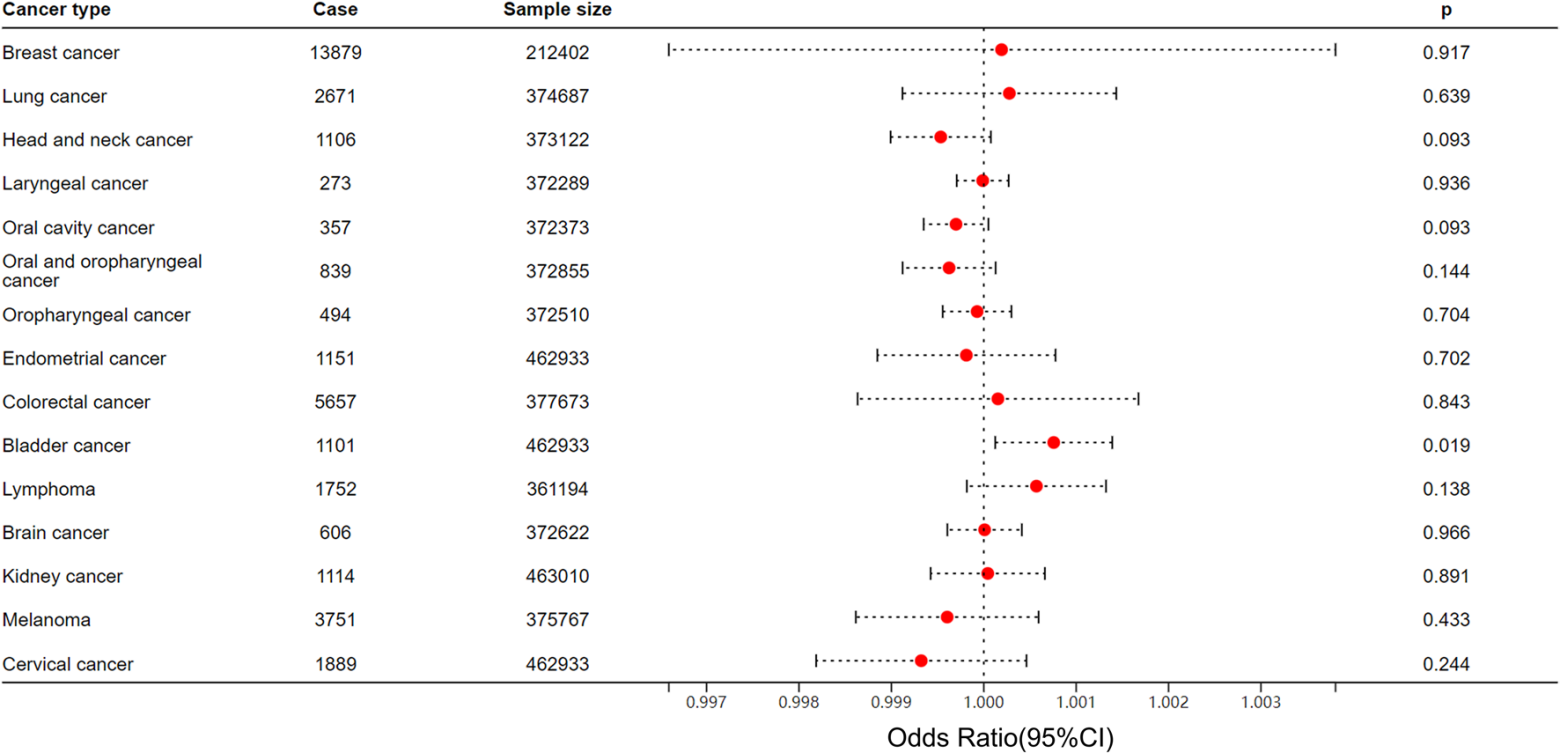
MR analysis unveiled the causal impact of endometriosis on diverse types of cancer. Notably, a significant correlation between endometriosis and a slightly elevated risk of bladder cancer was observed, which should be regarded as a false positive finding. No significant association between endometriosis and other cancer types could be discerned. Case, the number of patient who has been diagnosed with a specific type of cancer in the cohort; Sample size, the number of individuals involved in the study.

We proceeded to examine the causal influence of endometriosis on a wide range of common cancers, encompassing breast cancer, lung cancer, head and neck cancer, laryngeal cancer, oral cavity cancer, oral and oropharyngeal cancer, oropharyngeal cancer, endometrial cancer, colorectal cancer, bladder cancer, lymphoma, brain cancer, kidney cancer, melanoma, and cervical cancer (Supplementary Table 1). Unlike previous observational studies and meta-analyses, our findings did not reveal any significant association between endometriosis and breast cancer, or endometrial cancer^10,26,27^. However, we did identify a solitary significant causal impact of endometriosis on a slightly increased risk of bladder cancer (OR = 1.0008, 95% CI 1.0001–1.0014, *p* = 0.02). No heterogeneity was observed, as indicated by a Cochran Q-test derived *p*-value of 0.47 for IVW and a *p*-value of 0.51 for MR-Egger. Furthermore, there was no evidence of pleiotropy, with a *p*-value of 0.15. Nevertheless, in light of the OR = 1.0008 and *p* = 0.02, indicating a probable false positive result, we applied the Bonferroni correction method, adjusting the *p*-value to 0.0026, obtained by dividing 0.05 by 19. As a result, the relationship between endometriosis and the risk of bladder cancer should be regarded as a false positive finding. The forest plot depicting the causal impact of endometriosis on cancers is presented in Fig. 4. The scatter plots and funnel plots illustrating each pair of associations for casualty were presented in the supplementary materials (supplementary Figs. 1–19). The outcome of the Leave-one-out analysis suggested that there was no significant impact on the results (supplementary Figs. 20–38).

Finally, we have conducted statistical power analyses for each specified outcome, ascertaining that the statistical power for ovarian cancer, clear cell ovarian cancer, invasive mucinous ovarian cancer and endometrioid ovarian cancer exceeded 0.8, thereby further substantiating the credibility of our conclusions (Supplementary Table 4).

## Discussion

To the best of our knowledge, this is the first MR analysis examining the potential causal relationship between endometriosis and a wide range of cancers. Ultimately, we have discovered compelling evidence suggesting a significant causal effect of endometriosis on an increased risk of ovarian cancer, especially clear cell ovarian cancer, and endometrioid ovarian cancer. However, we did not find any significant association between endometriosis and other types of cancer.

The relationship between endometriosis and the risk of cancer has been controversial for tens of years. In 2002, Olson et al. reported that endometriosis was not associated with an elevated risk of cancers in a cohort study including 37,434 participants^28^. However, in 2007 Melin et al. found that endometriosis was associated with elevated risks for endocrine tumors, ovarian cancer, renal cancer, thyroid cancer, brain tumours, malignant melanoma, and breast cancer, as well as a reduced risk for cervical cancer after excluding cancers already present at the time of endometriosis diagnosis^9^. Kok et al. reported increased risks of all cancers, ovarian cancer, and endometrial cancer^7^. In 2018, Saraswat et al. conducted a cohort study including 281 937 women with almost 5 million person-years of follow-up and found that women with surgically diagnosed endometriosis would face an increased risk of ovarian cancer^5^. Additionally, the majority of previous epidemiological studies were designed as case–control studies and were unable to establish a clear causal relationship due to a lack of precise temporal sequencing. Even in prospective cohort studies, where cancer patients at the time of endometriosis diagnosis were excluded, there still remained a reverse effect on undiagnosed subclinical cancer that potentially contribute to the development of endometriosis. Therefore, it is plausible that endometriosis may not serve as a predictive factor for cancer development, but rather be an outcome resulting from the presence of cancer. Furthermore, previous observational studies faced challenges in avoiding confounding risk factors. However, in the current study, we were able to overcome these limitations by employing Mendelian randomization methods, which allowed us to establish a more robust causal relationship and mitigate the bias inherent in the study design. In a recent investigation concerning the relationship between endometriosis and epithelial ovarian cancer, a correlation was identified between endometriosis and the risk of developing epithelial ovarian cancer, endometrioid carcinoma, clear cell carcinoma, and low malignant potential tumors^29^. This finding aligns with our conclusions but was derived from a distinct database utilizing Mendelian randomization analysis, further substantiating the reliability of the findings presented in our manuscript.

The pathogenesis of cancer is exceedingly intricate^30–35^. Endometriosis and cancer share many similarities, particularly their ability to proliferate cells in oxygen-deprived environments, their invasive nature, and their capacity to induce tissue remodeling, vascularization, and innervation^36^. Endometriotic tissue often contains cancer-associated mutations that are frequently observed in ovarian cancers associated with endometriosis^37^. In recent years, there has been a growing body of evidence indicating the presence of somatic mutations in cancer-associated genes including ARID1A, PIK3CA, KRAS, or PPP2R1A in endometriotic lesions^38–40^. These well-known genes are also commonly subject to mutations in ovarian cancers associated with endometriosis^41^. These findings provide additional validation for our discoveries, highlighting a causal relationship between endometriosis and elevated susceptibility to ovarian cancer. This revelation stimulates novel pathways of inquiry into the underlying mechanisms of endometriosis, potentially paving the way for the development of a biologically-driven classification system that enhances prognostication and enables precise therapeutic approaches.

Our findings unequivocally validate a causal association between endometriosis and an elevated risk of ovarian cancer, aligning with the conclusions drawn from a recent comprehensive meta-analysis^10^. Furthermore, our investigation delved deeper into the correlation between endometriosis and histological subtypes of ovarian cancer, unearthing a more pronounced link between endometriosis and an increased risk of clear cell ovarian cancer and endometrioid ovarian cancer. In contrast to prior observational studies and meta-analyses, we discovered a significant causal influence of endometriosis on a modestly heightened risk of bladder cancer, while no association between endometriosis and other types of cancer was observed. However, result of Bonferroni correction method indicated that the link between endometriosis and the risk of bladder cancer should be regarded as a false positive finding. Notebaly, a recent study showed a causal relationship between endometriosis and a decreased risk of breast cancer (odds ratio [OR] 0.95; 95% CI 0.90–0.99, *p* = 0.02)^42^. Given the application of the Bonferroni correction method, we advocate caution in interpreting the association between endometriosis and a reduced risk of overall breast cancer. More studies should be performed to unveil the underlying pathways between endometriosis and cancers, both at biological and behavioral levels.

Our study possesses several notable strengths. Firstly, employing the MR design allows us to emulate randomized controlled trials within observational settings. Randomized controlled trials are widely recognized as the gold standard for establishing causality, but they are often prohibitively expensive and logistically challenging to conduct. However, MR studies effectively mitigate confounding biases by depositing the random assignment of SNPs at conception. In comparison to other observational studies, MR also circumvents the issue of reverse causality. Secondly, our findings have the potential to significantly impact healthcare strategies for both endometriosis and cancer. Given the high prevalence of endometriosis in the general population, establishing a causal link between endometriosis and cancer has important implications for public health policies, particularly in terms of early prevention and timely intervention. Our findings suggest that intensifying ovarian cancer screening for patients with endometriosis may yield benefits. Further investigations are warranted to elucidate the underlying pathways connecting endometriosis and various human cancers.

However, it is crucial to acknowledge several limitations in our study. Firstly, all GWAS data utilized in our analysis were derived from European populations. Therefore, the generalizability of our findings to other populations remains to be explored. Secondly, it is important to recognize that we were unable to eliminate all potential confounding factors associated with cancers, which may have implications for the accuracy of our conclusions. Finally, our primary focus was on specific cancer types, notably ovarian and breast cancer, owing to their well-established associations with endometriosis. Nevertheless, we did not analyze the potential association between endometriosis and other cancer types, such as bone tumors.

In conclusion, we have uncovered compelling evidence indicating a causal relationship between endometriosis and an elevated risk of ovarian cancer, particularly clear cell ovarian cancer and endometrioid ovarian cancer. However, our investigation did not reveal any significant associations between endometriosis and other types of cancer. To comprehensively elucidate the underlying mechanisms connecting endometriosis and various cancers, further studies are warranted, encompassing both biological and behavioral aspects.

### Supplementary Information

Supplementary Table 1.

Supplementary Table 2.

Supplementary Table 3.

Supplementary Table 4.

Supplementary Information 1.

## Data Availability

Some databases supporting this study’s findings are openly available and described in ‘Methods’. The exposure data was obtained from the FinnGen database and can be accessed via https://www.finngen.fi/en. The outcome data was obtained from IEU open GWAS project (https://gwas.mrcieu.ac.uk/datasets/) and the GWAS ID was listed in Supplementary Table 1. Other data are available from the corresponding author.
