# Supplementary material for: Genetically identification of endometriosis and cancers risk in women through a two-sample Mendelian randomization study
Source: Sci Rep. 2024 Apr 10;14:8382. doi: 10.1038/s41598-024-58950-7 (PMC11006903; doi:10.1038/s41598-024-58950-7)
Supplement: Supplementary file 5 — Supplementary Information 1. [file 41598_2024_58950_MOESM5_ESM.pdf]

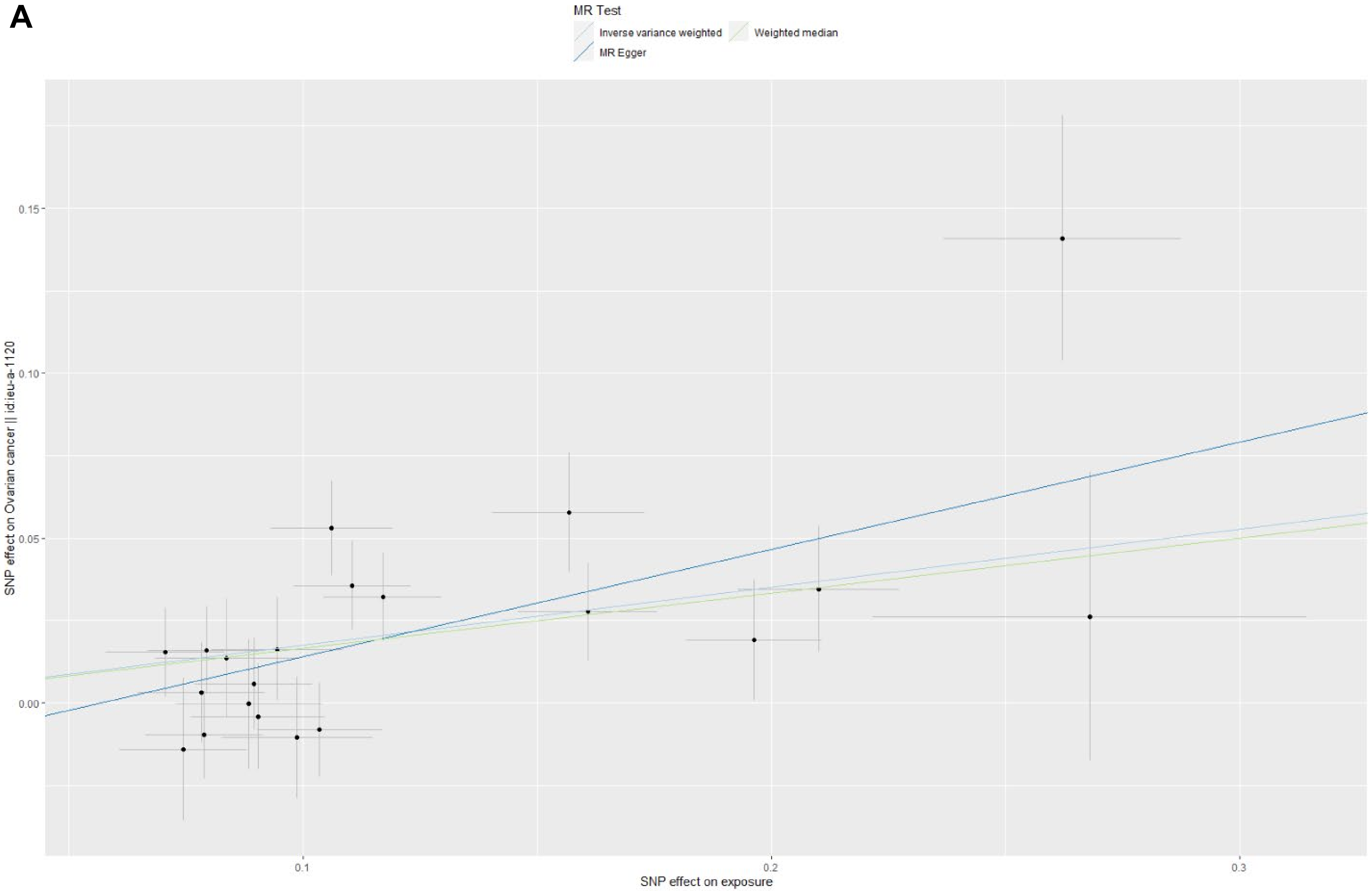

Supplementary Figure 1. Scatter plot (A) and funnel plot (B) of the causal efect of endometriosis on ovarian cancer.

B

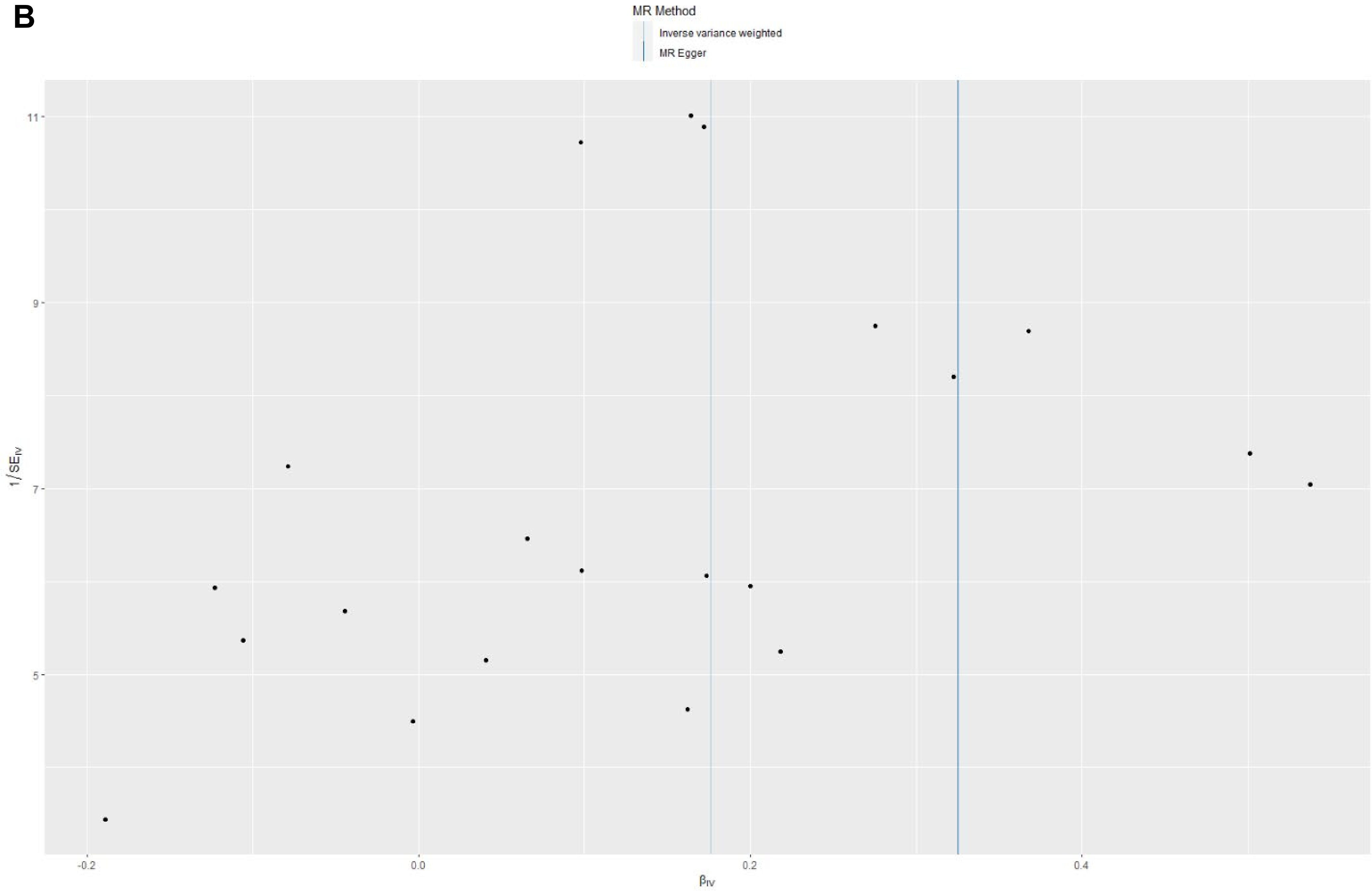

Supplementary Figure 1. Scatter plot (A) and funnel plot (B) of the causal efect of endometriosis on ovarian cancer.

A

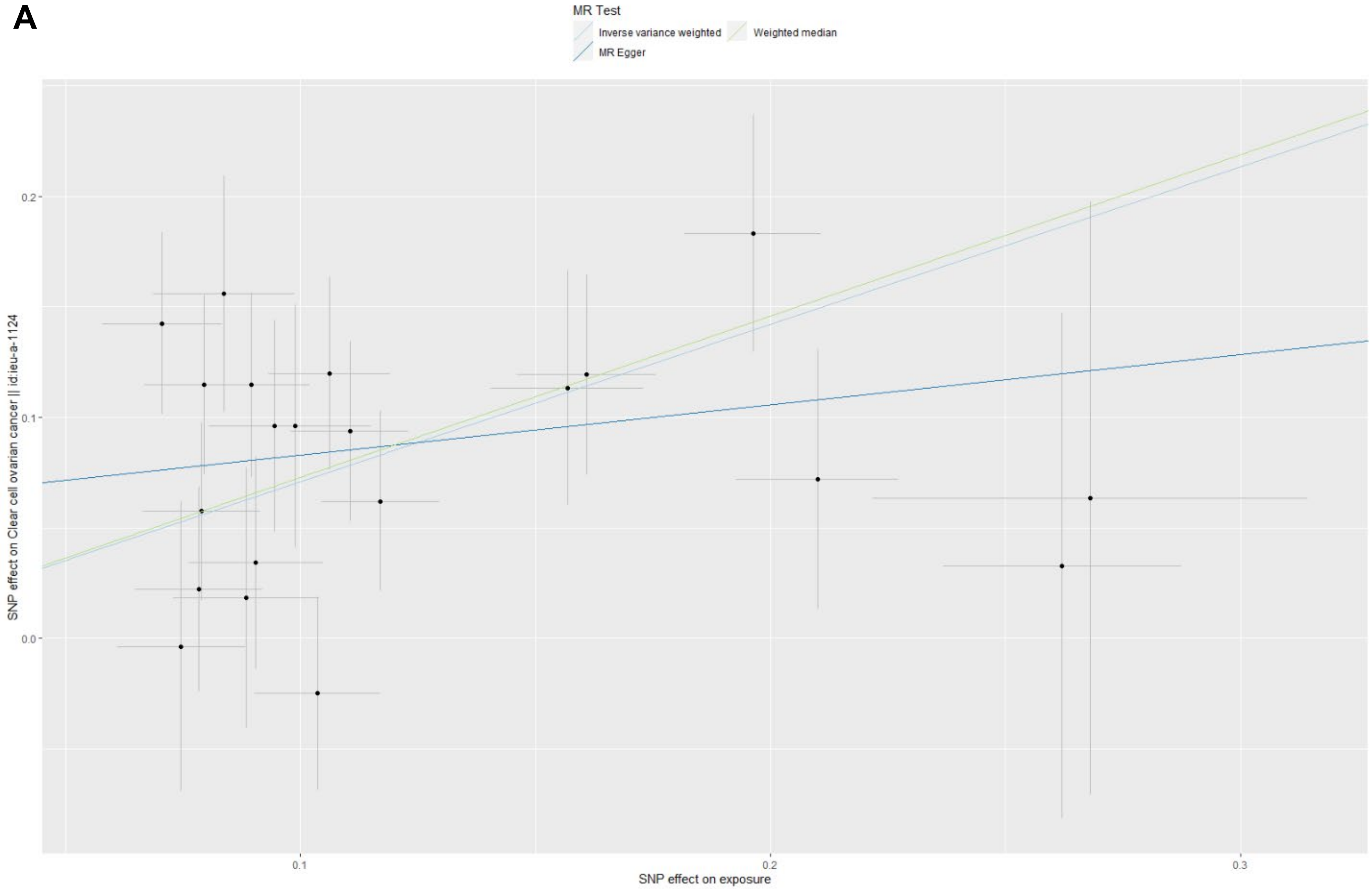

Supplementary Figure 2. Scatter plot (A) and funnel plot (B) of the causal efect of endometriosis on clear cell ovarian cancer.

B

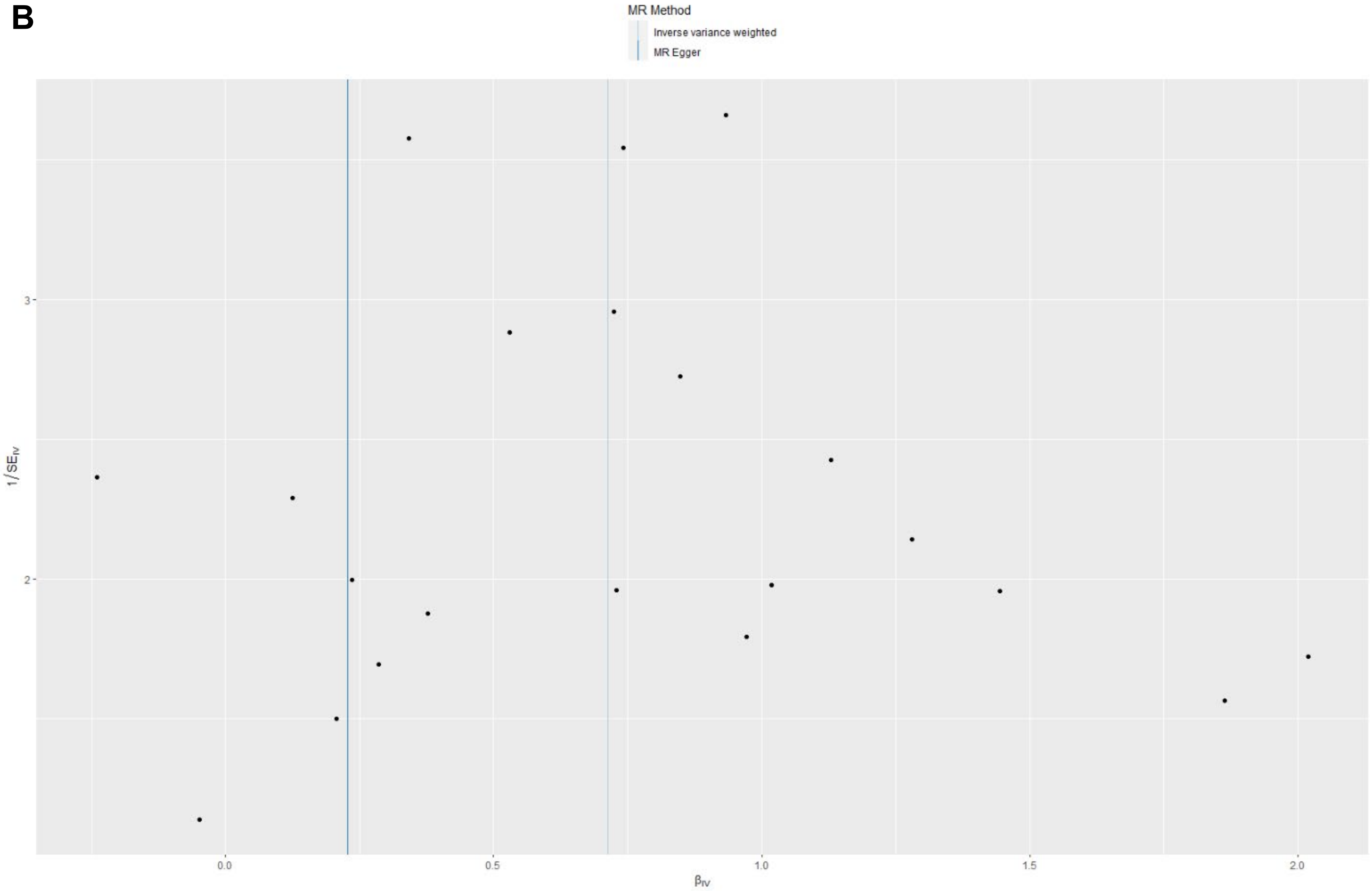

Supplementary Figure 2. Scatter plot (A) and funnel plot (B) of the causal efect of endometriosis on clear cell ovarian cancer.

A

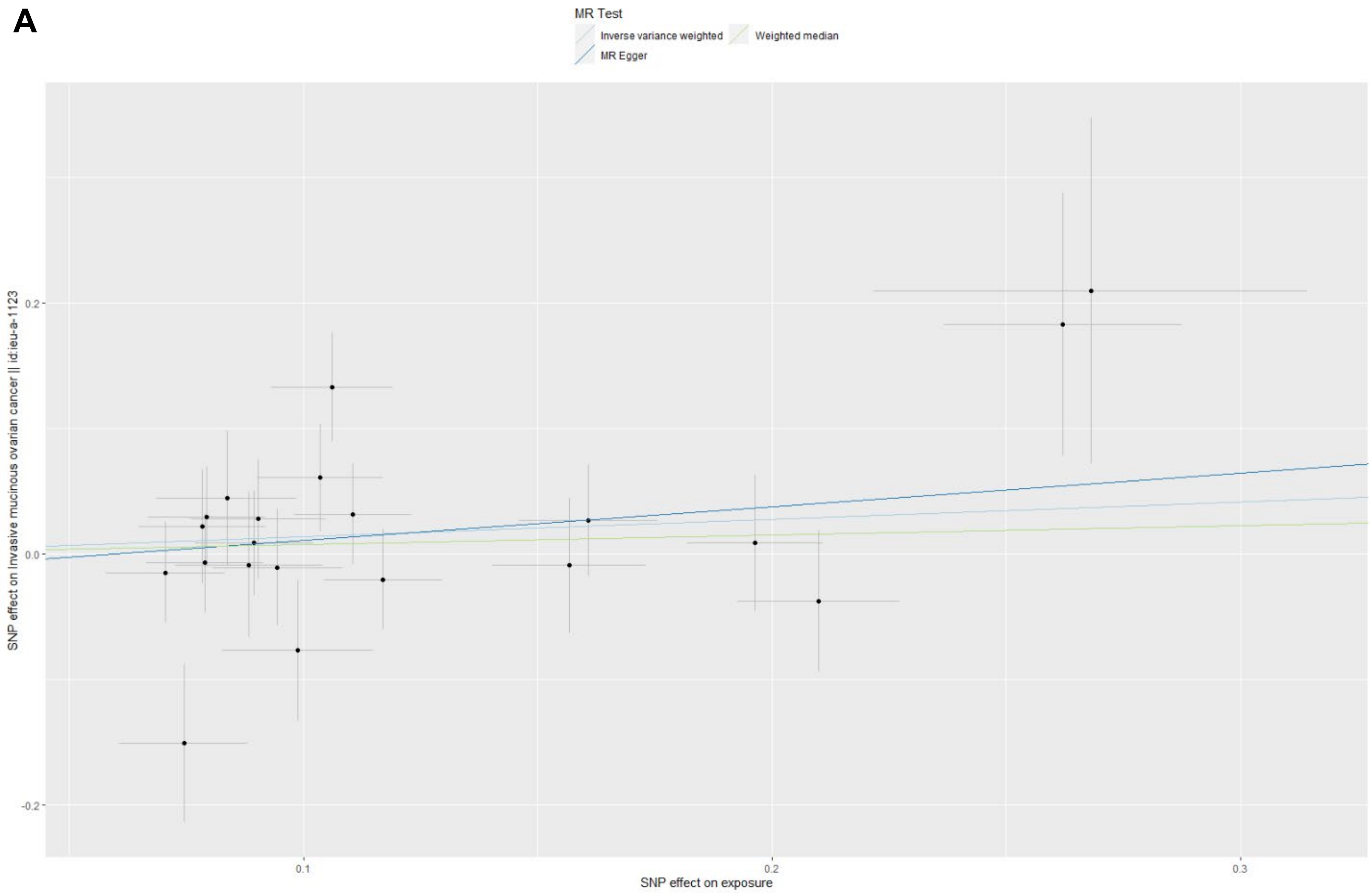

Supplementary Figure 3. Scatter plot (A) and funnel plot (B) of the causal efect of endometriosis on invasive mucinous ovarian cancer.

B

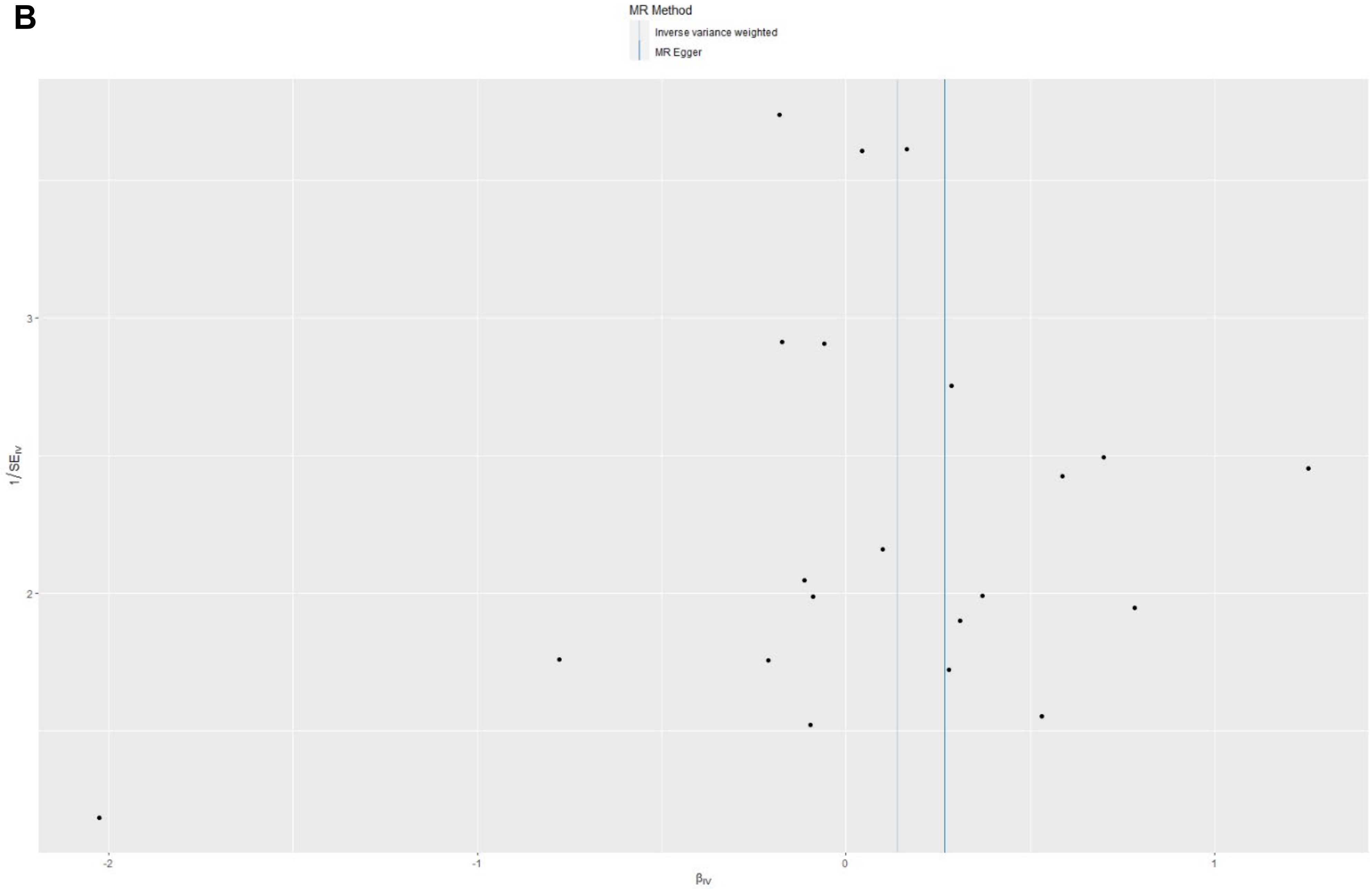

Supplementary Figure 3. Scatter plot (A) and funnel plot (B) of the causal efect of endometriosis on invasive mucinous ovarian cancer.

A

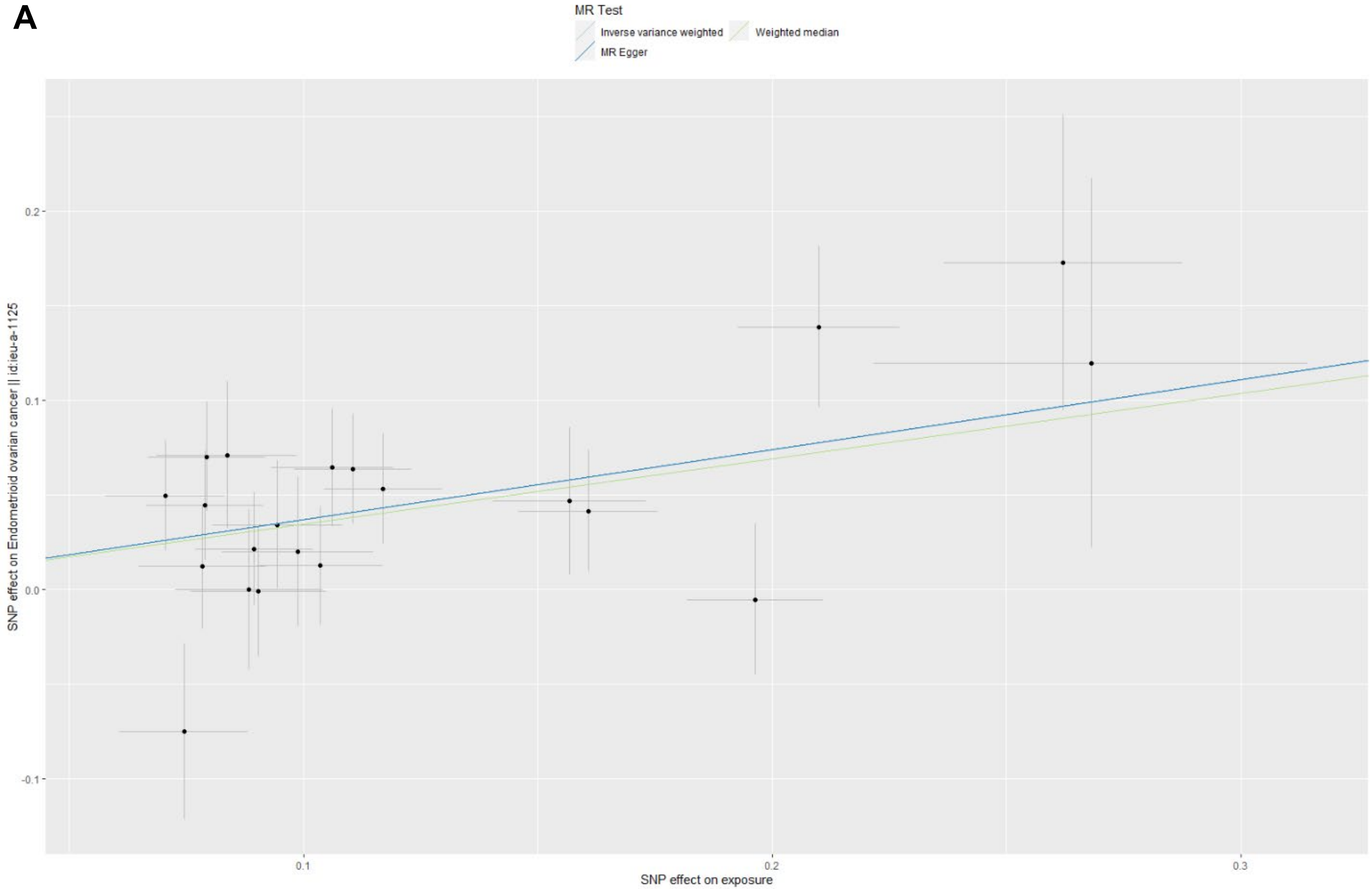

Supplementary Figure 4. Scatter plot (A) and funnel plot (B) of the causal efect of endometriosis on endometrioid ovarian cancer.

B

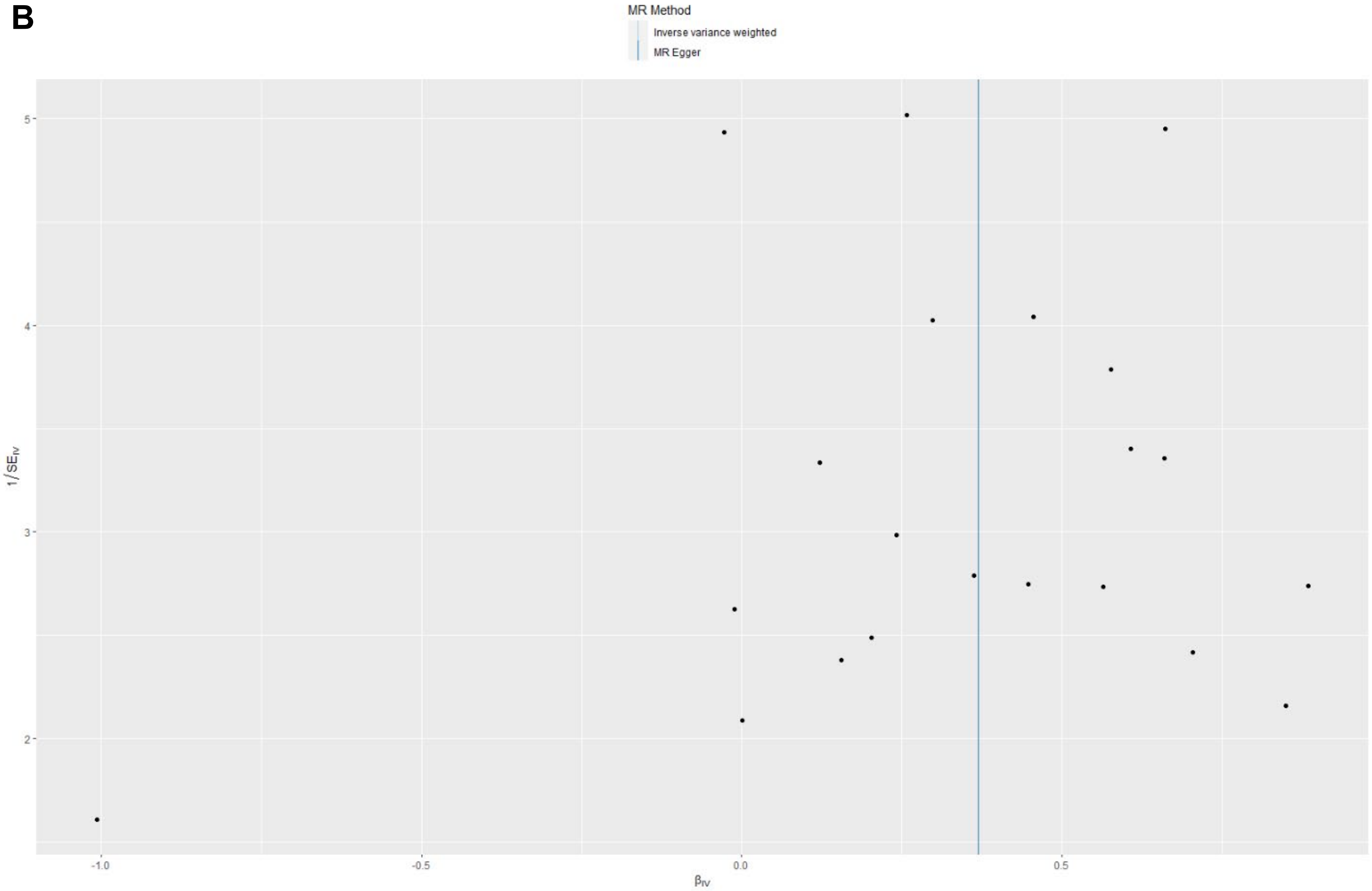

Supplementary Figure 4. Scatter plot (A) and funnel plot (B) of the causal efect of endometriosis on endometrioid ovarian cancer.

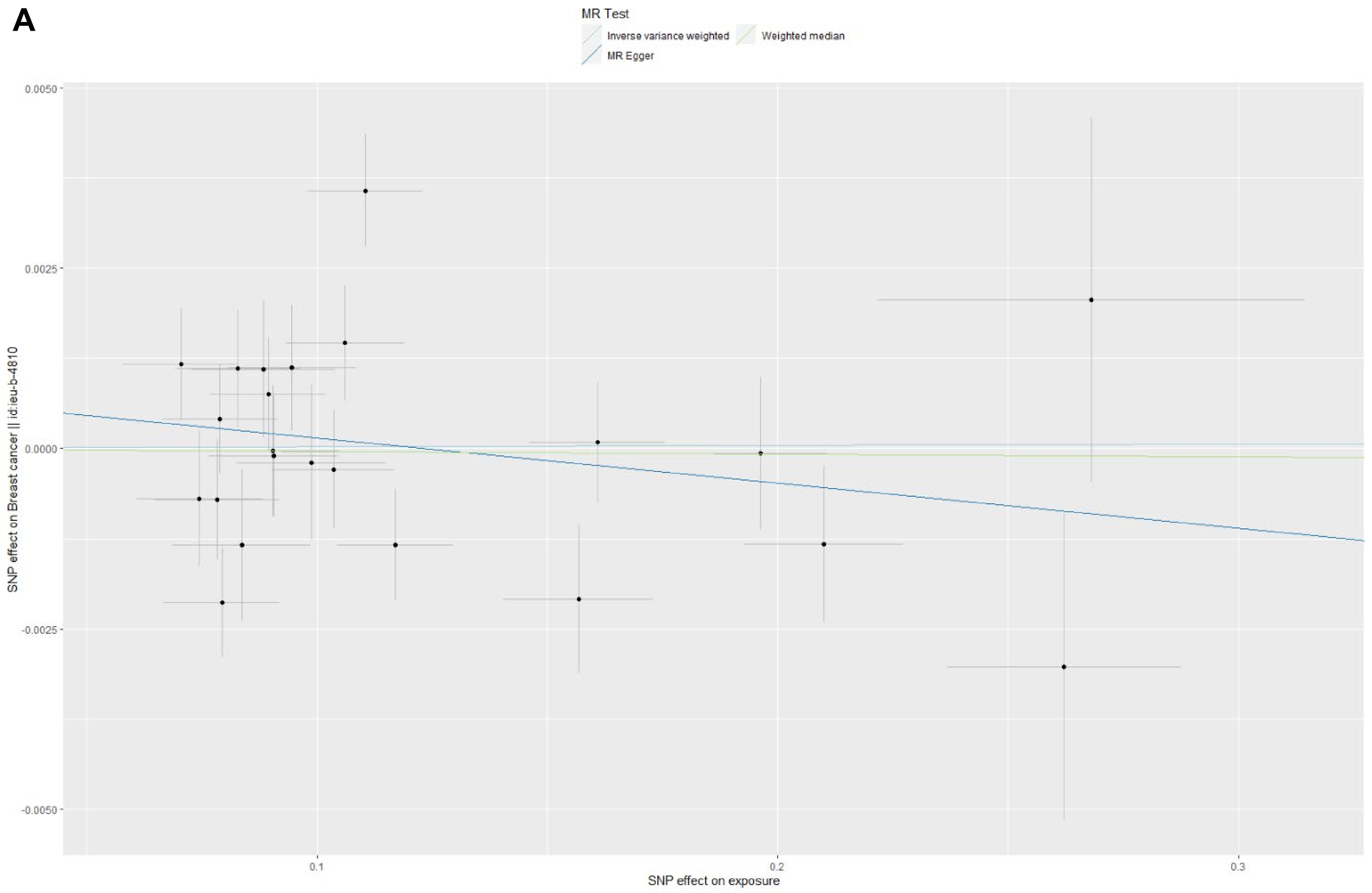

Supplementary Figure 5. Scatter plot (A) and funnel plot (B) of the causal efect of endometriosis on breast cancer.

B

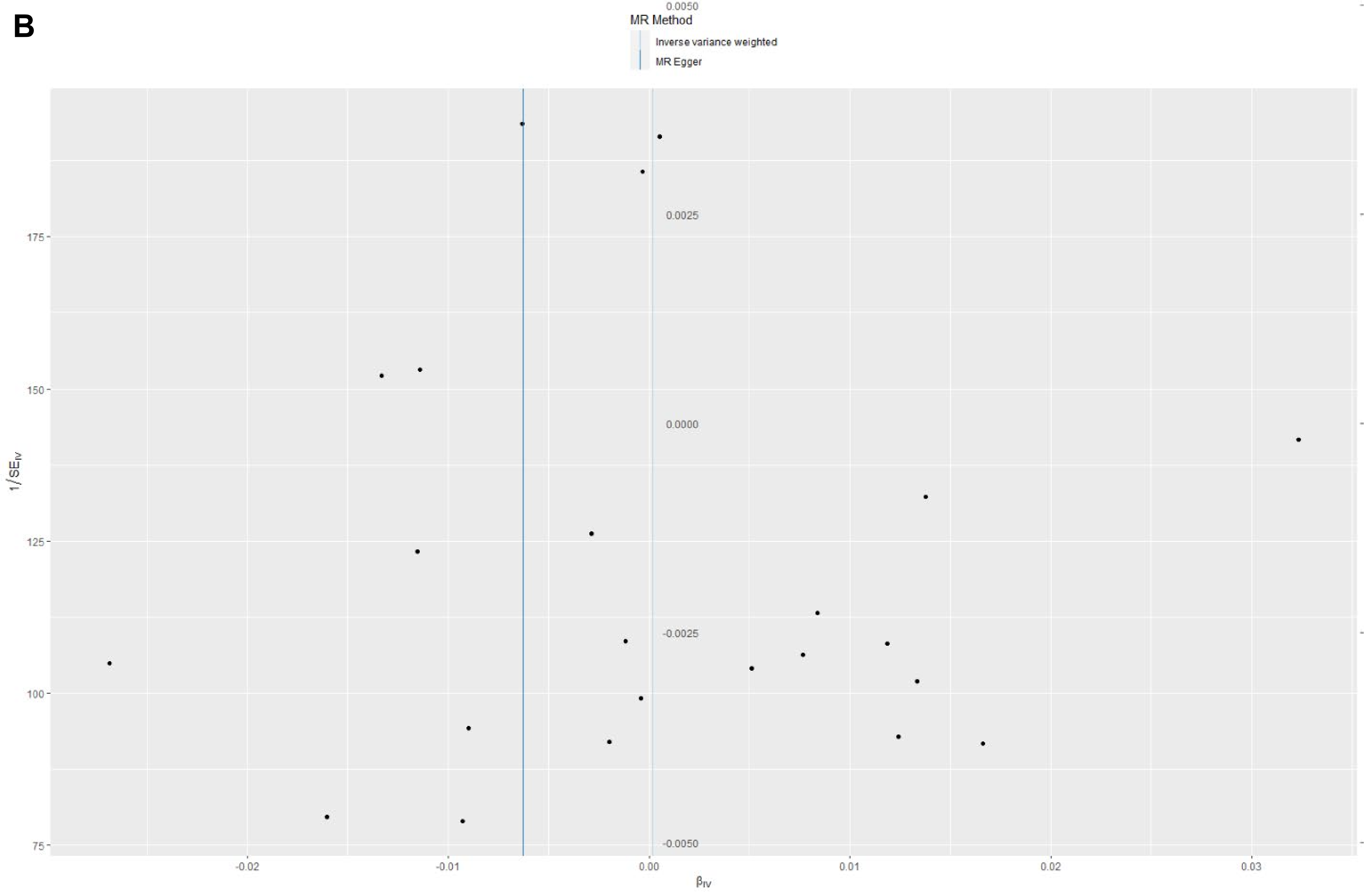

Supplementary Figure 5. Scatter plot (A) and funnel plot (B) of the causal efect of endometriosis on breast cancer.

A

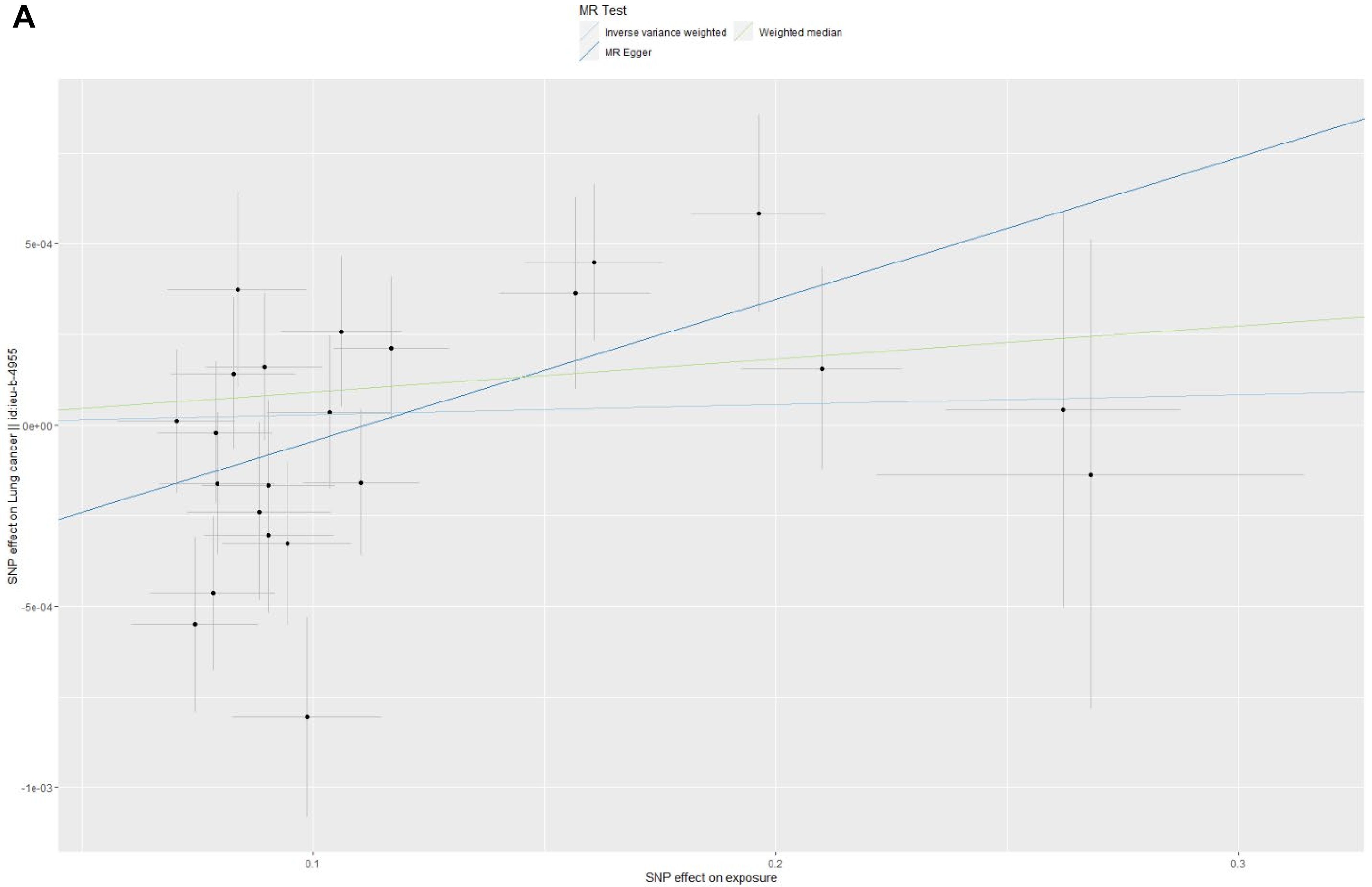

Supplementary Figure 6. Scatter plot (A) and funnel plot (B) of the causal efect of endometriosis on lung cancer.

B

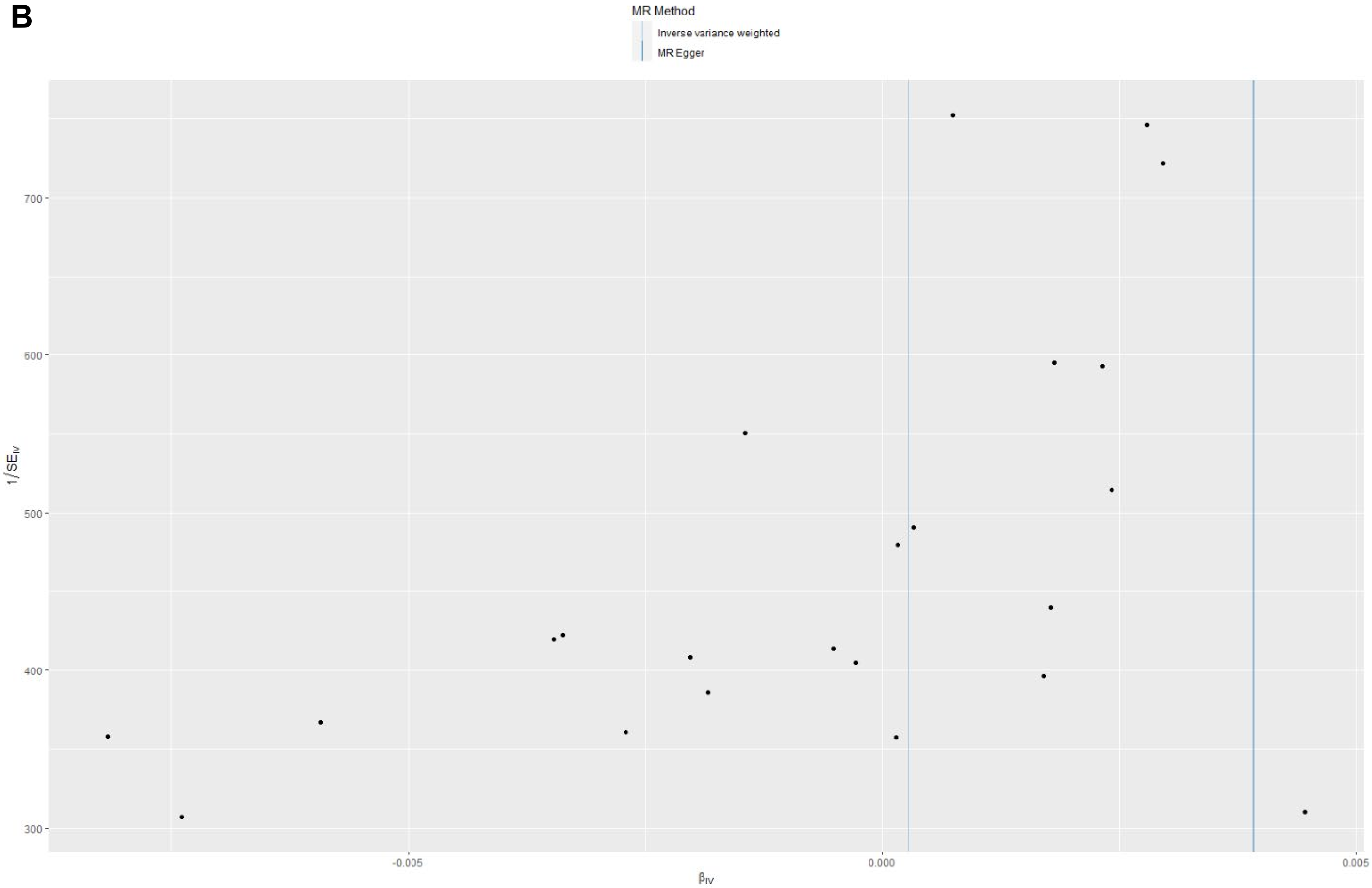

Supplementary Figure 6. Scatter plot (A) and funnel plot (B) of the causal efect of endometriosis on lung cancer.

A

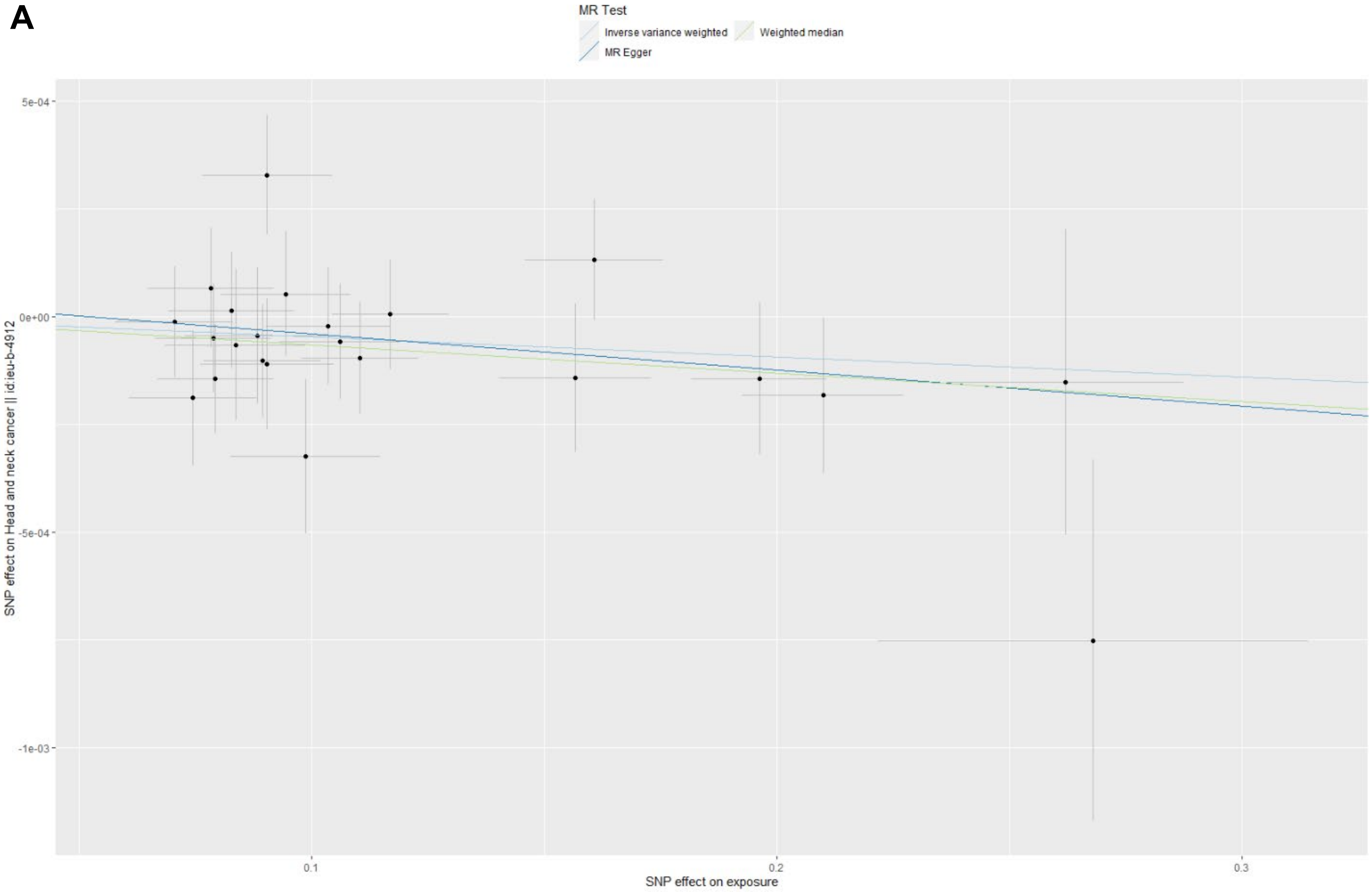

Supplementary Figure 7. Scatter plot (A) and funnel plot (B) of the causal efect of endometriosis on head and neck cancer.

B

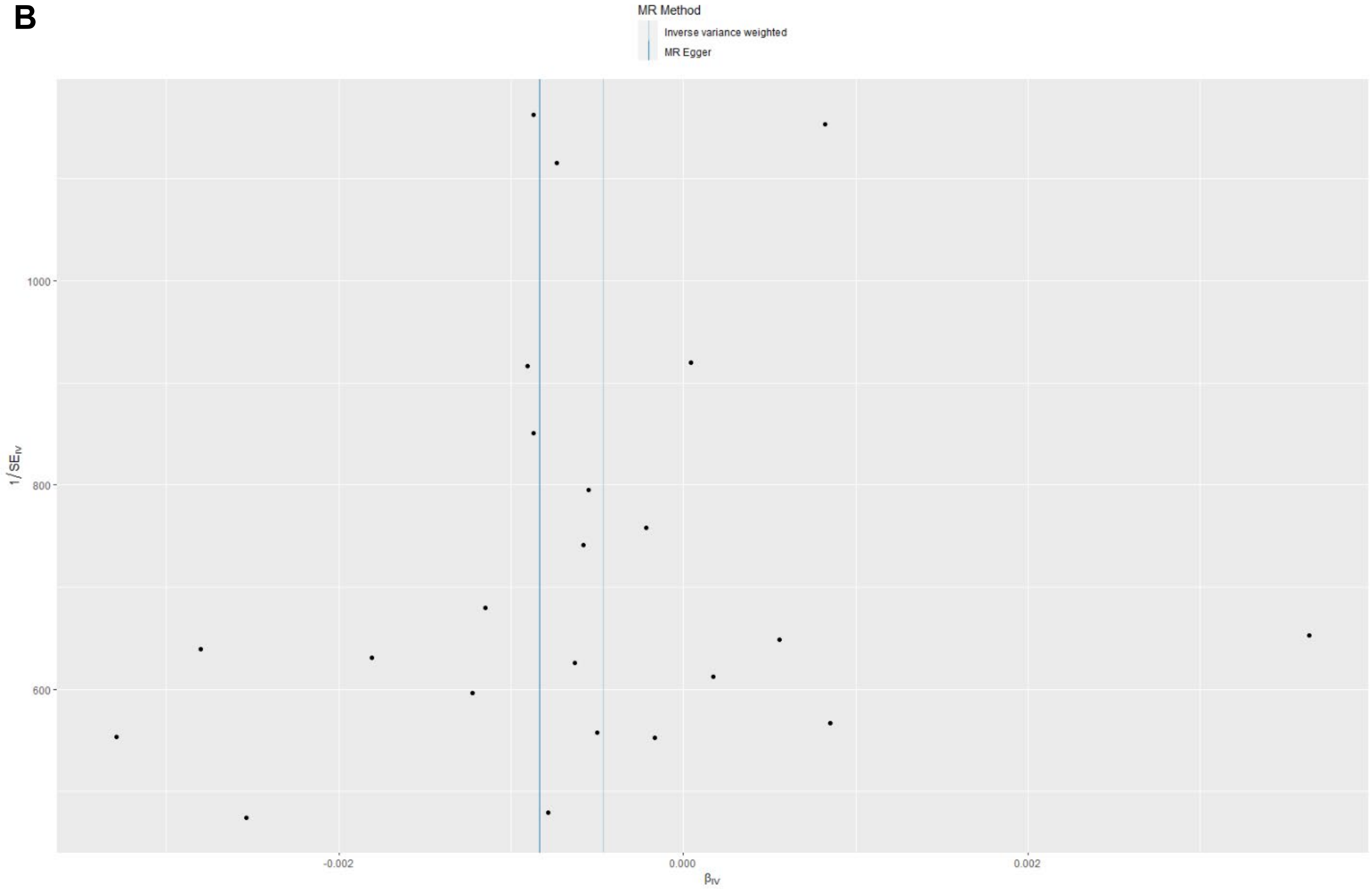

Supplementary Figure 7. Scatter plot (A) and funnel plot (B) of the causal efect of endometriosis on head and neck cancer.

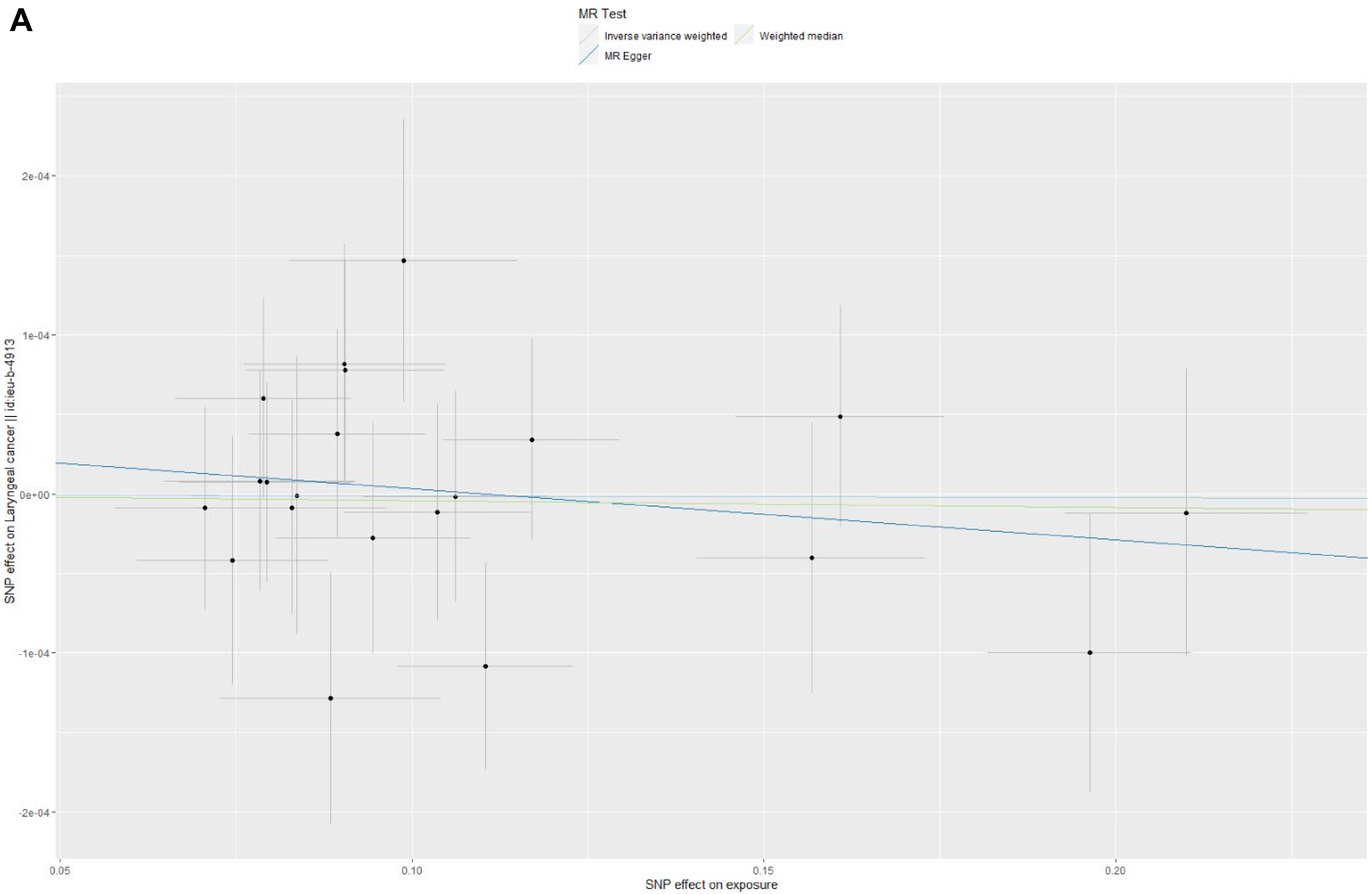

Supplementary Figure 8. Scatter plot (A) and funnel plot (B) of the causal efect of endometriosis on laryngeal cancer.

B

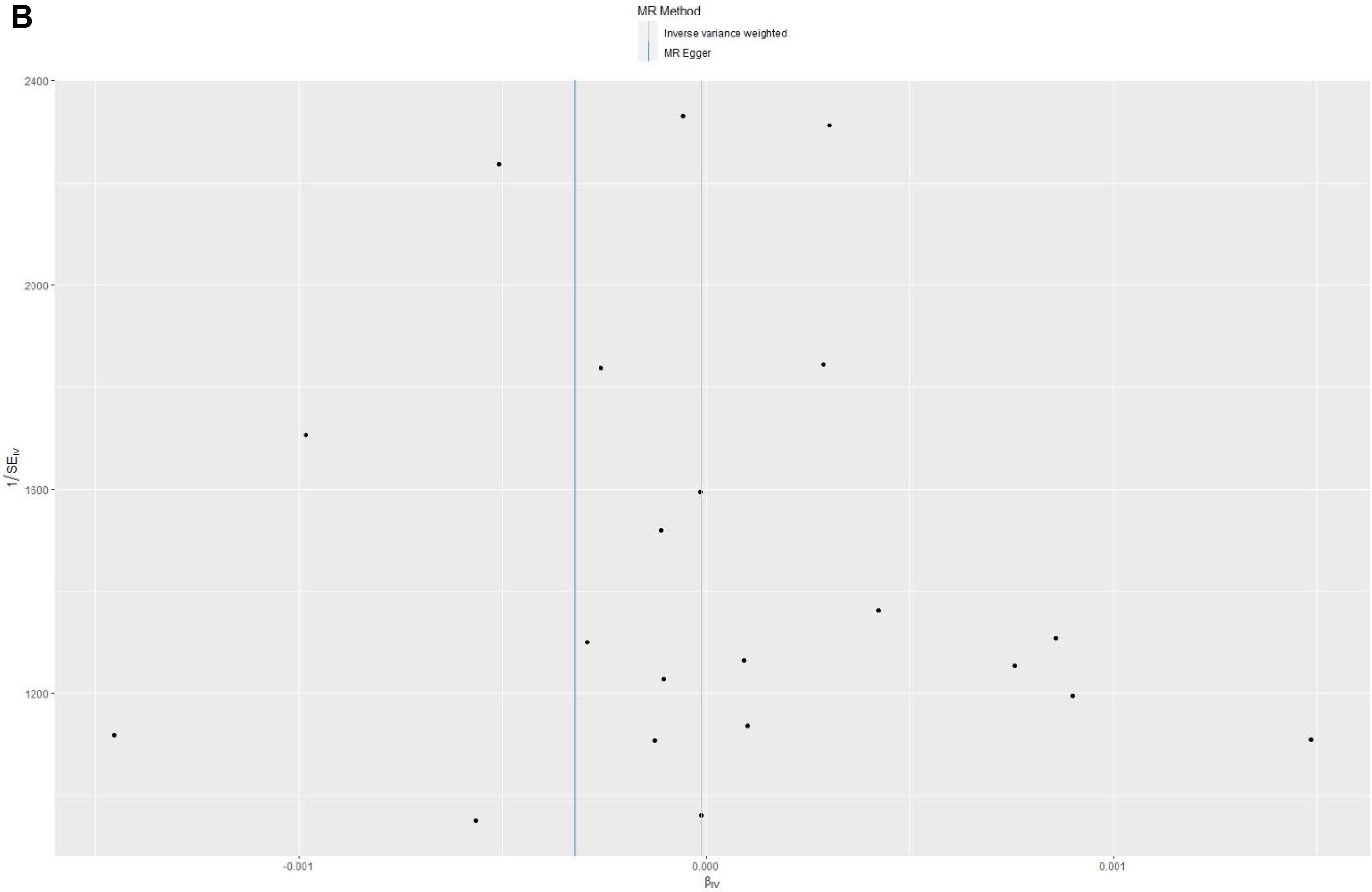

Supplementary Figure 8. Scatter plot (A) and funnel plot (B) of the causal efect of endometriosis on laryngeal cancer.

A

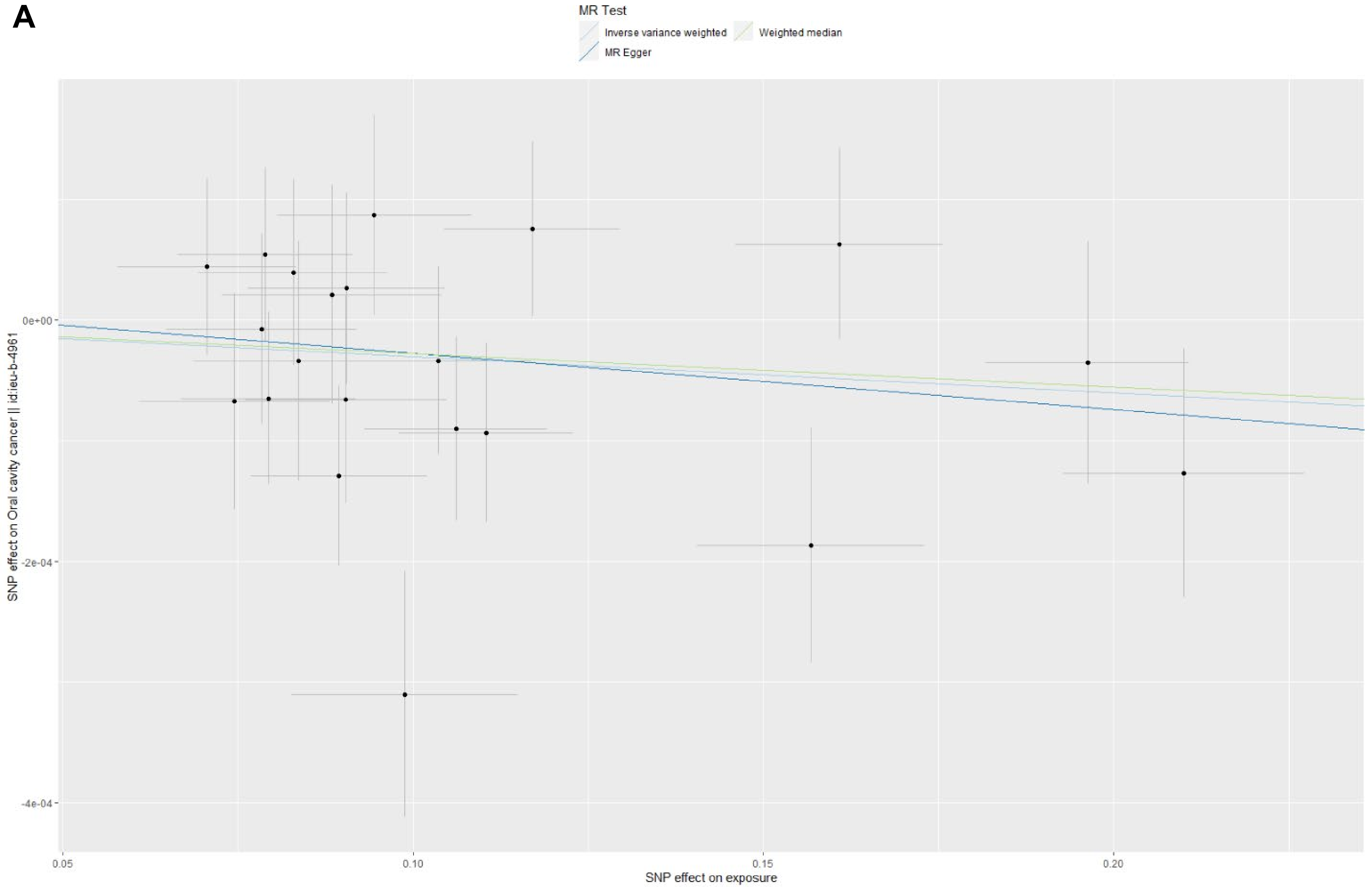

Supplementary Figure 9. Scatter plot (A) and funnel plot (B) of the causal efect of endometriosis on oral cavity cancer.

B

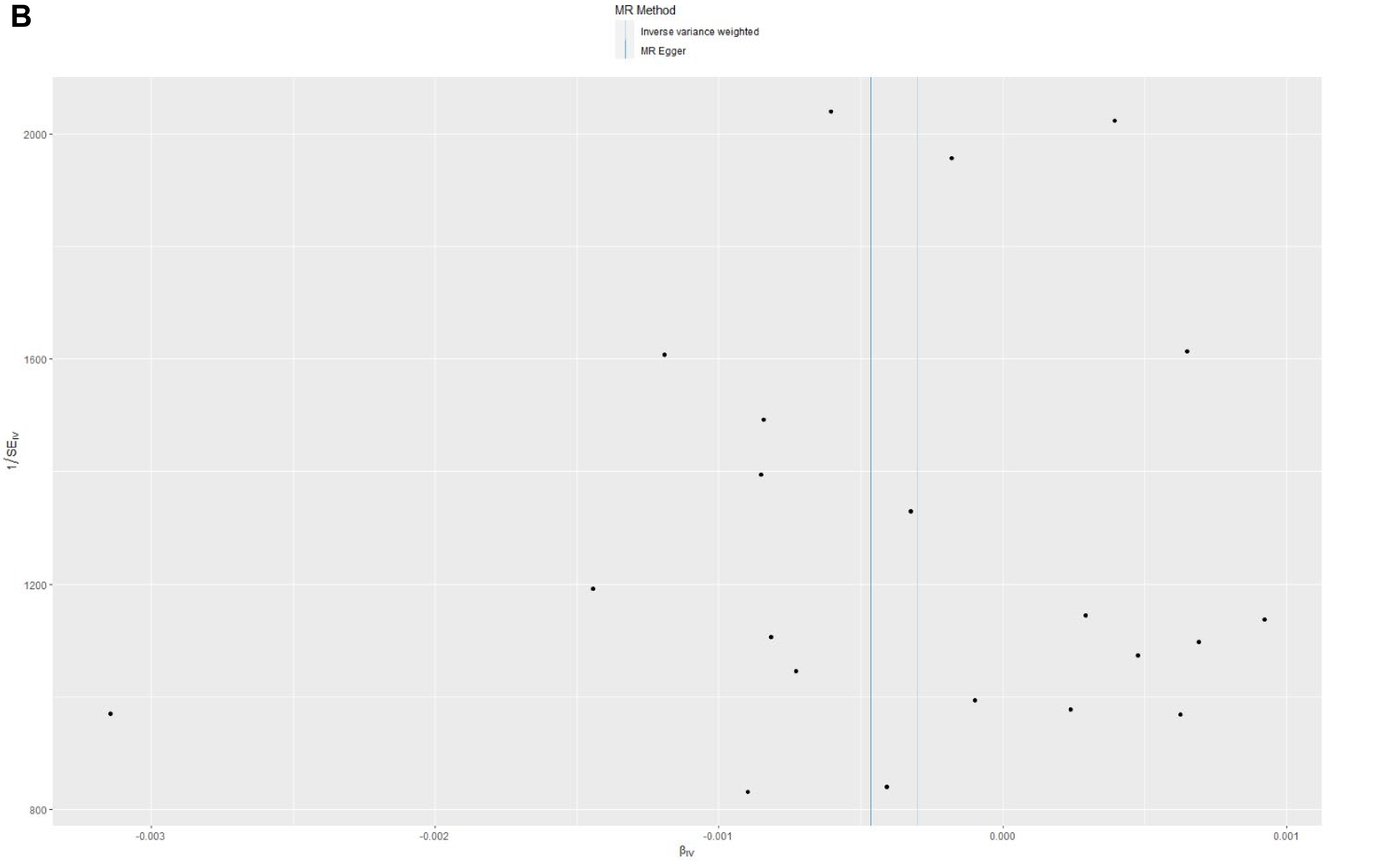

Supplementary Figure 9. Scatter plot (A) and funnel plot (B) of the causal efect of endometriosis on oral cavity cancer.

A

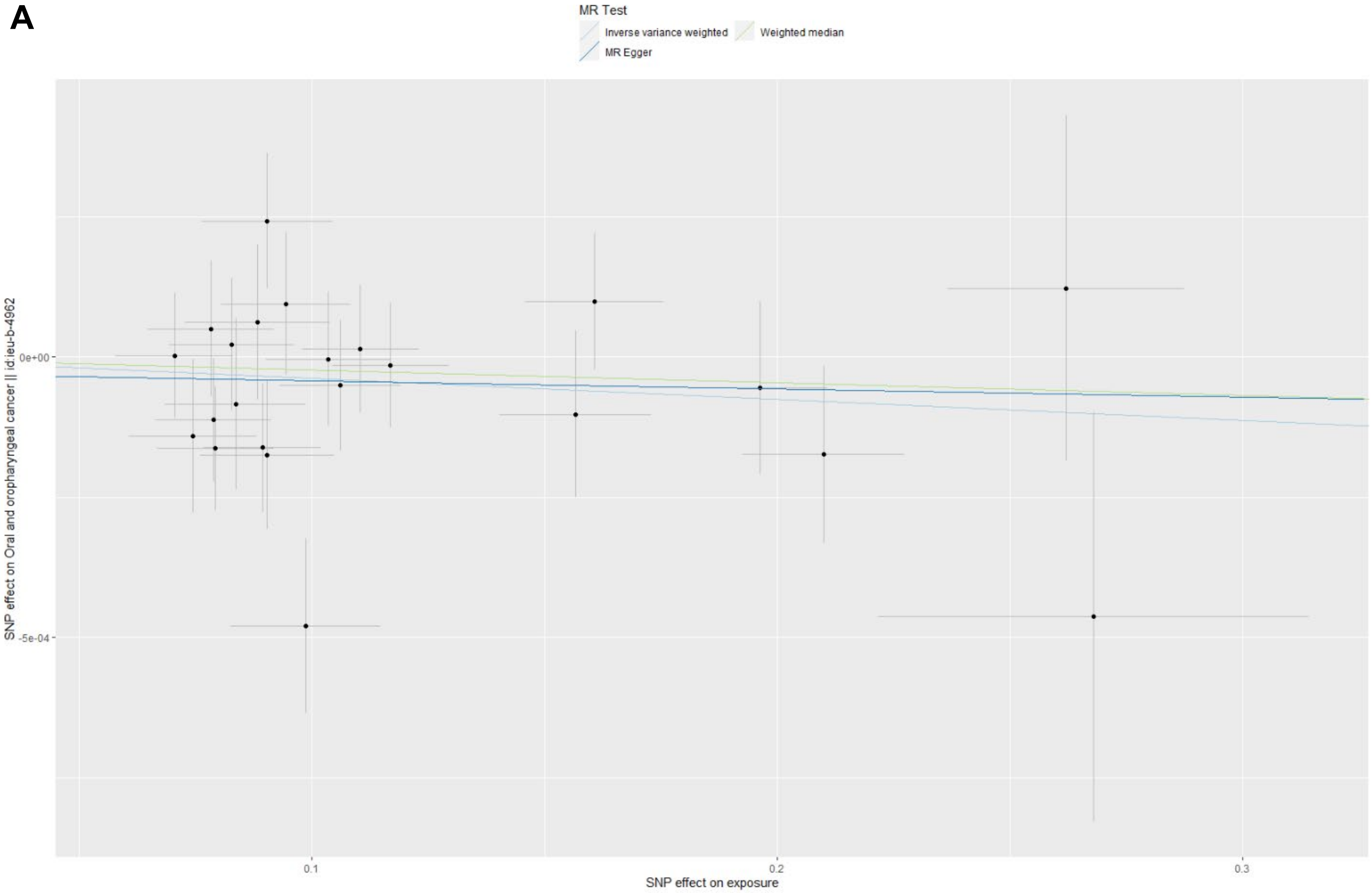

Supplementary Figure 10. Scatter plot (A) and funnel plot (B) of the causal efect of endometriosis on oral and oropharyngeal cancer .

B

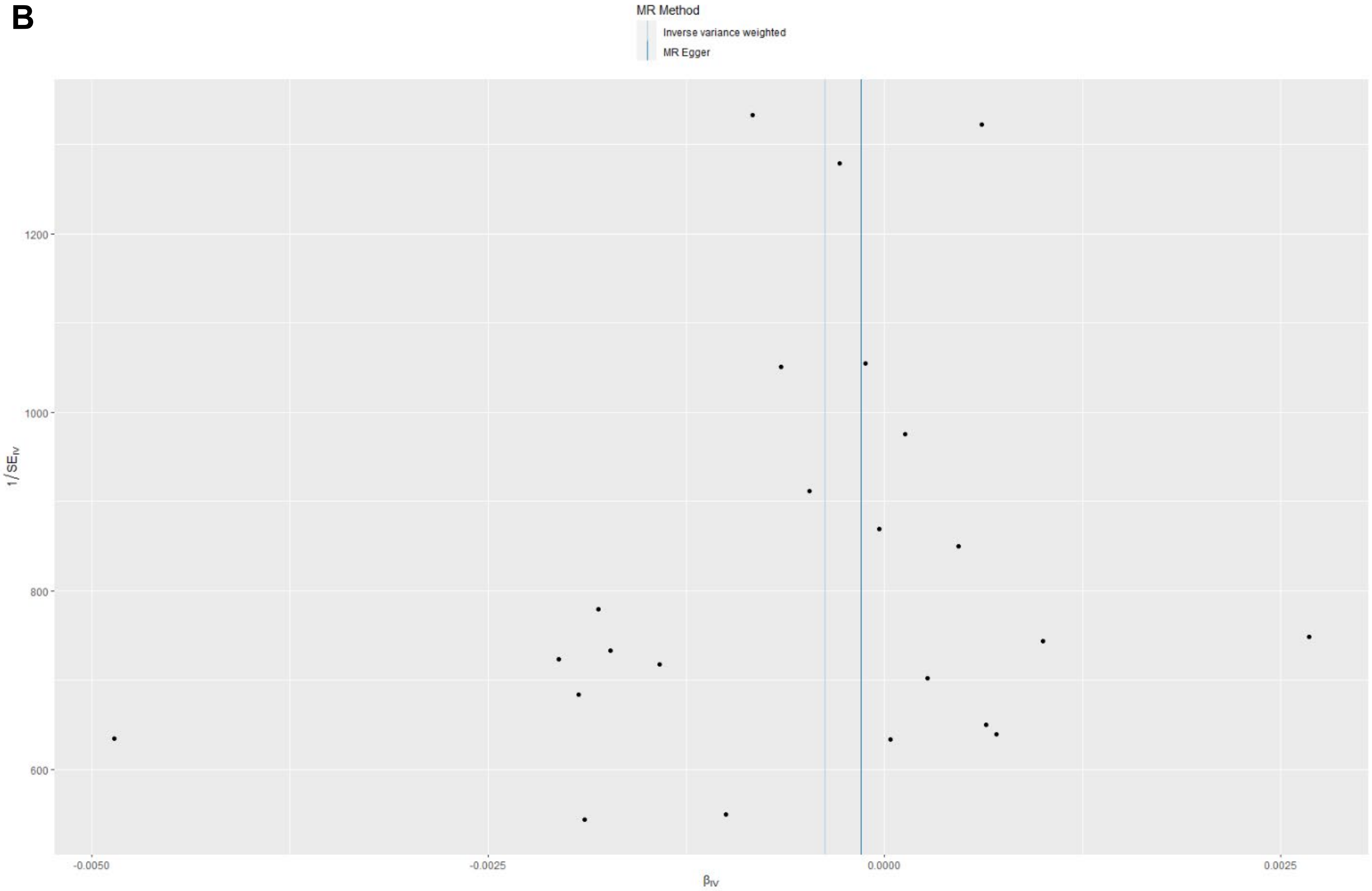

Supplementary Figure 10. Scatter plot (A) and funnel plot (B) of the causal efect of endometriosis on oral and oropharyngeal cancer .

A

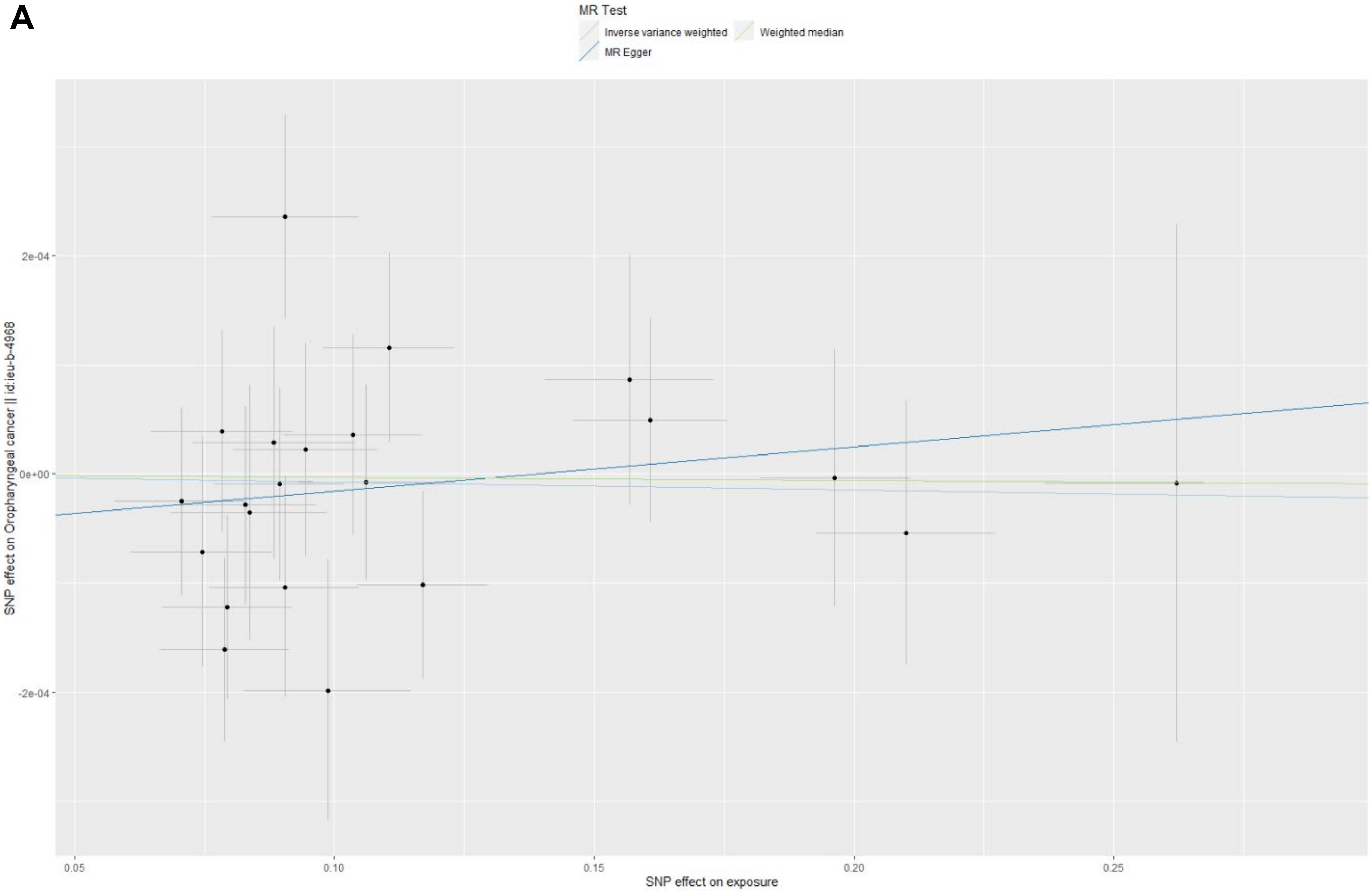

Supplementary Figure 11. Scatter plot (A) and funnel plot (B) of the causal efect of endometriosis on oropharyngeal cancer.

B

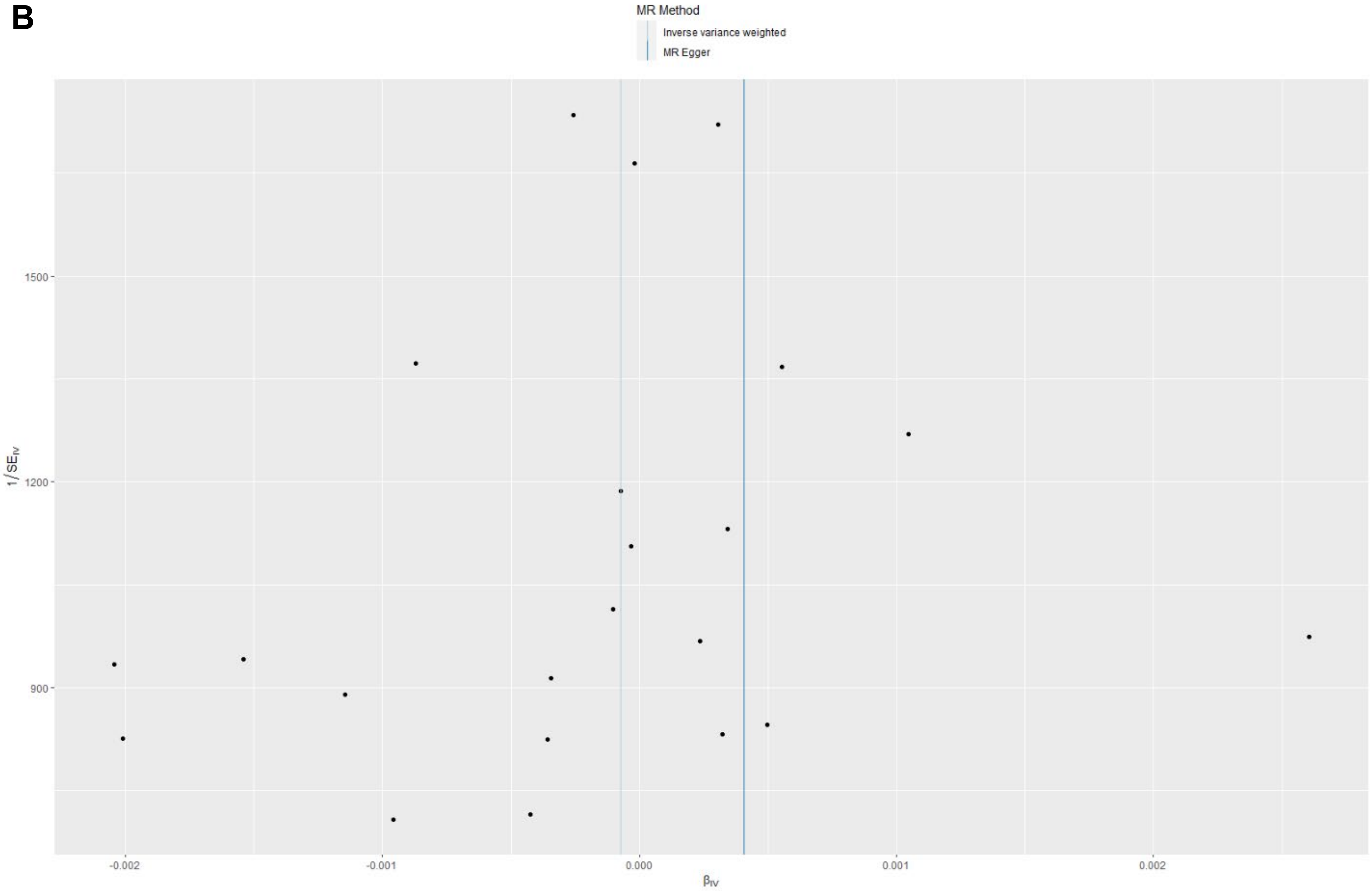

Supplementary Figure 11. Scatter plot (A) and funnel plot (B) of the causal efect of endometriosis on oropharyngeal cancer.

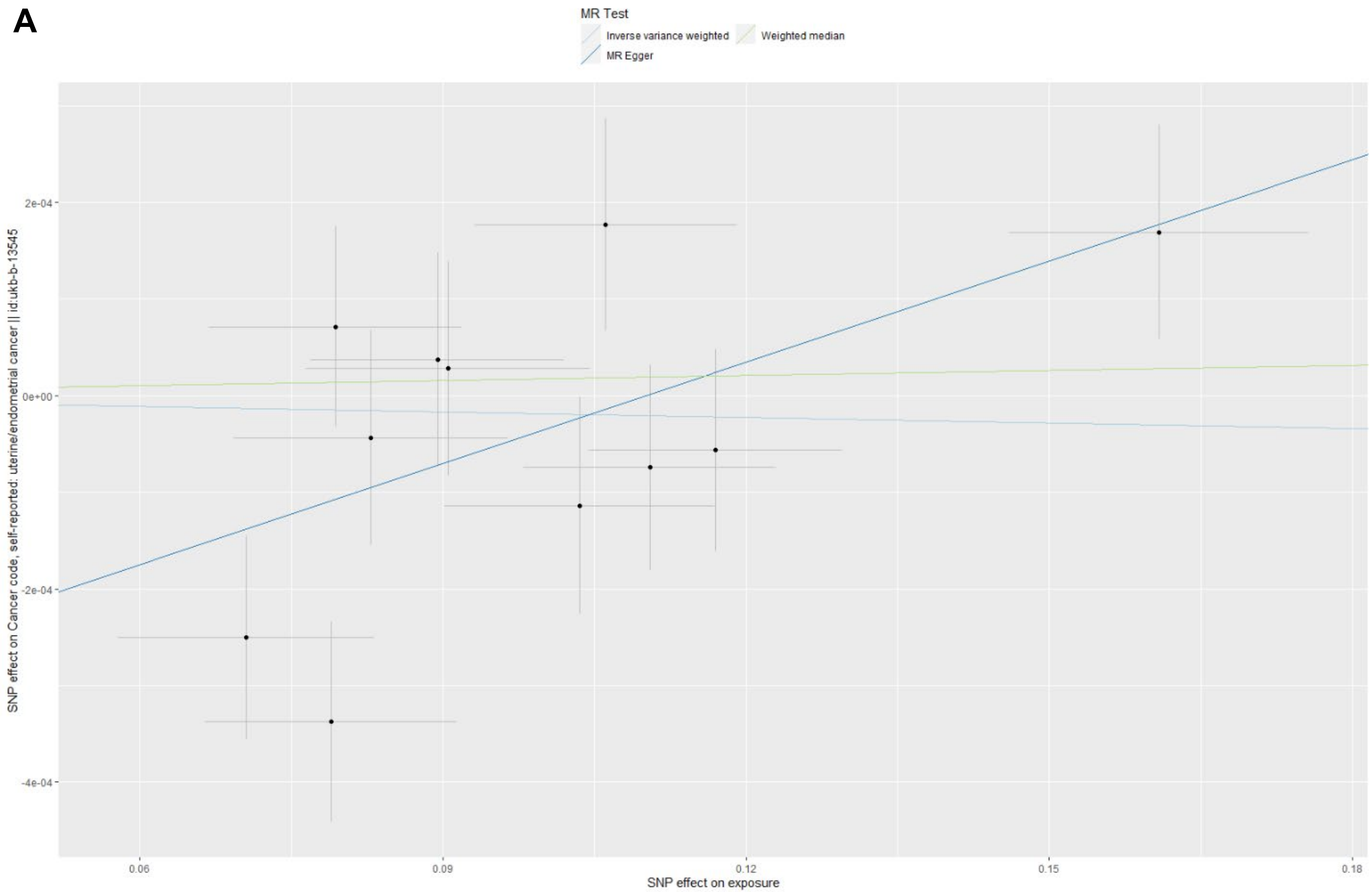

Supplementary Figure 12. Scatter plot (A) and funnel plot (B) of the causal efect of endometriosis on endometrial cancer.

B

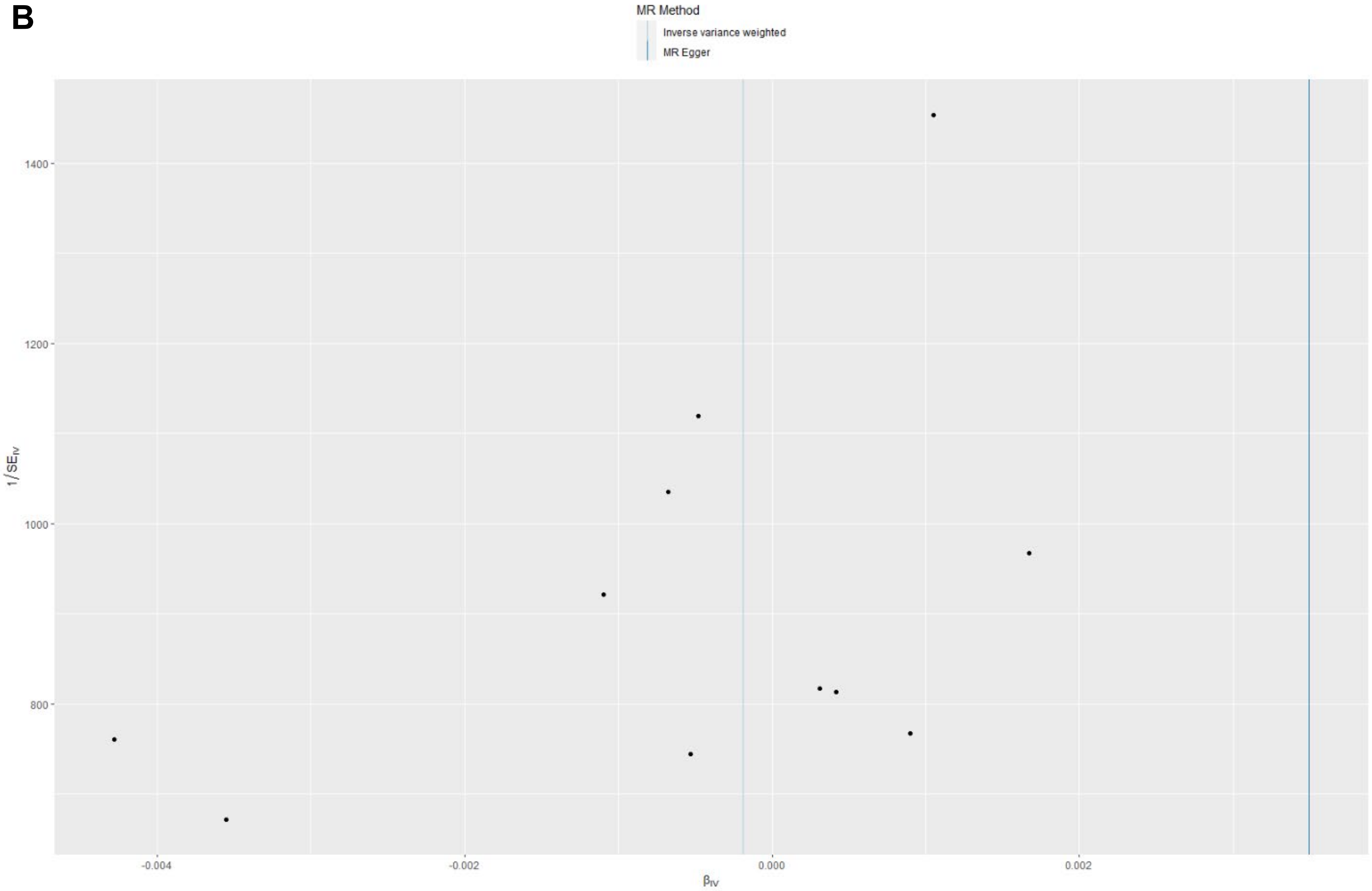

Supplementary Figure 12. Scatter plot (A) and funnel plot (B) of the causal efect of endometriosis on endometrial cancer.

A

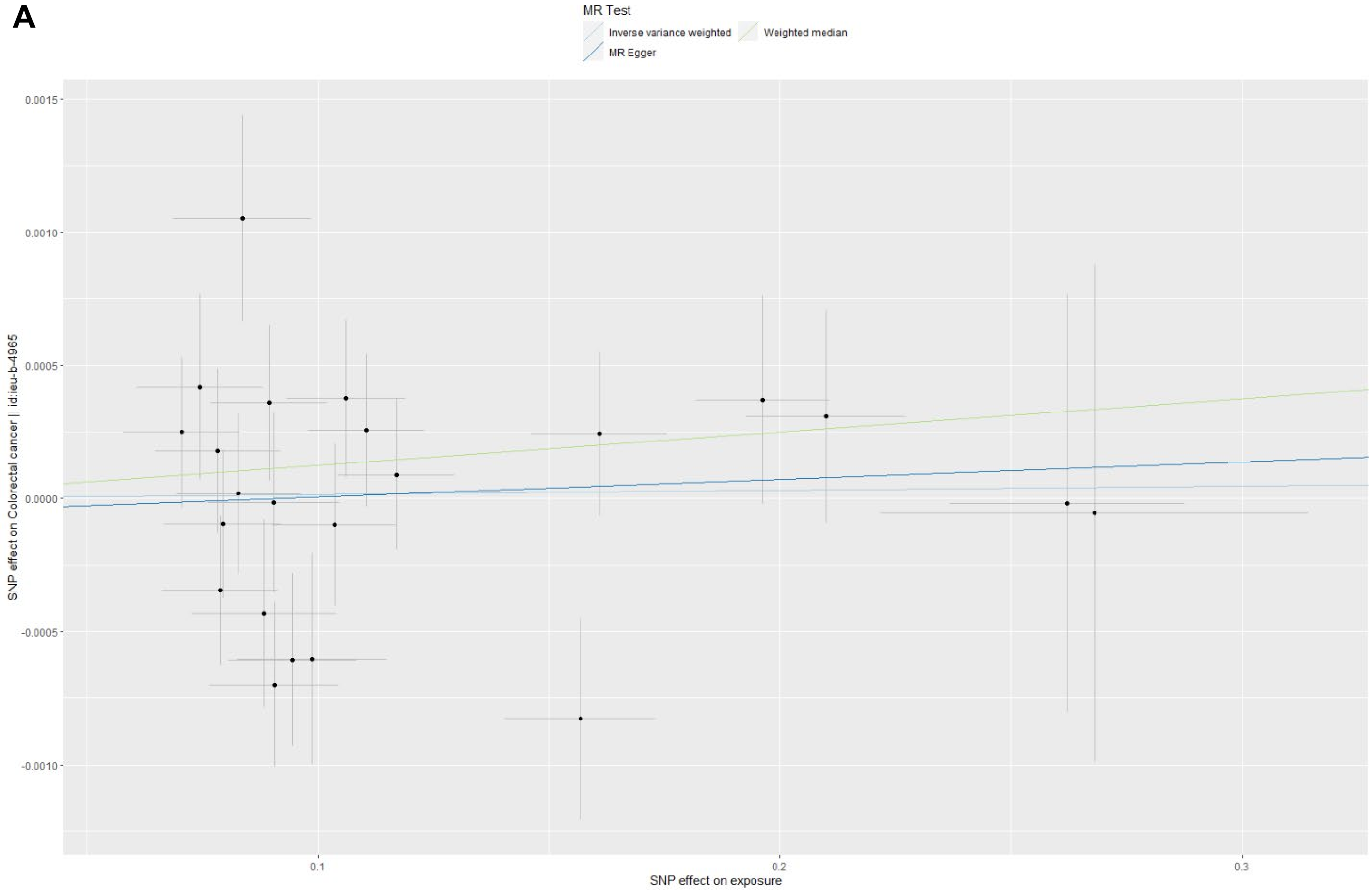

Supplementary Figure 13. Scatter plot (A) and funnel plot (B) of the causal efect of endometriosis on colorectal cancer.

B

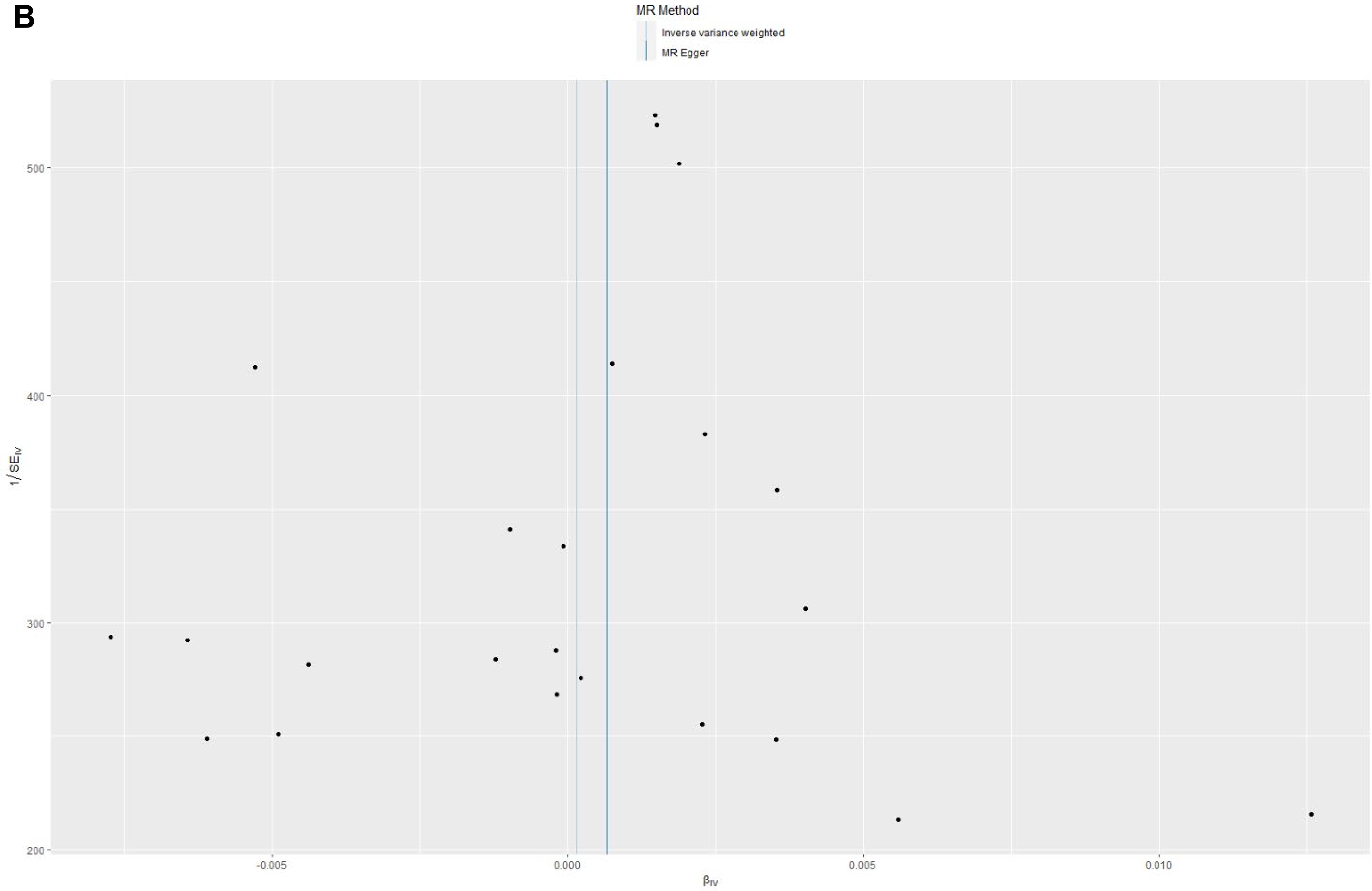

Supplementary Figure 13. Scatter plot (A) and funnel plot (B) of the causal efect of endometriosis on colorectal cancer.

A

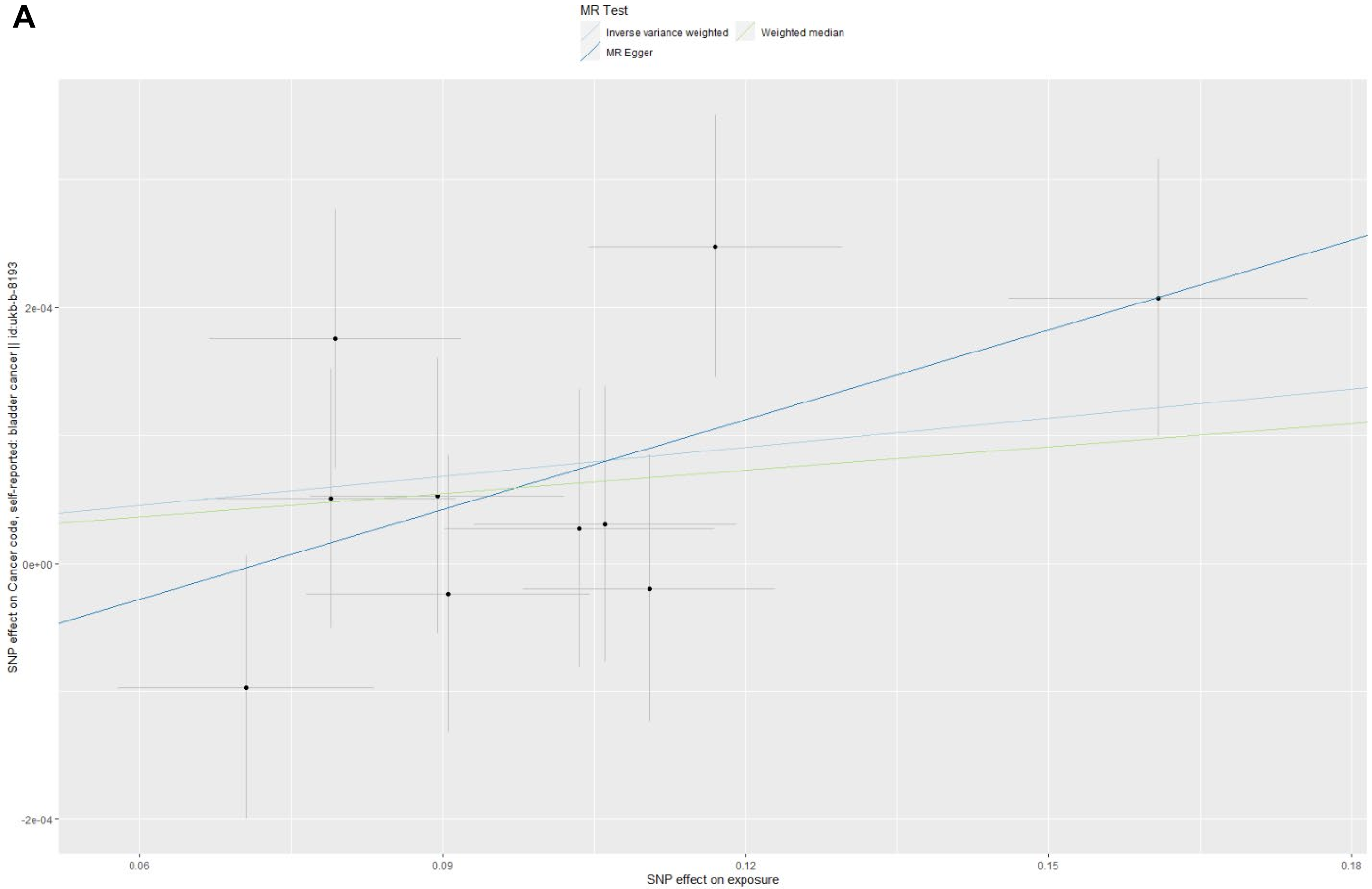

Supplementary Figure 14. Scatter plot (A) and funnel plot (B) of the causal efect of endometriosis on bladder cancer.

B

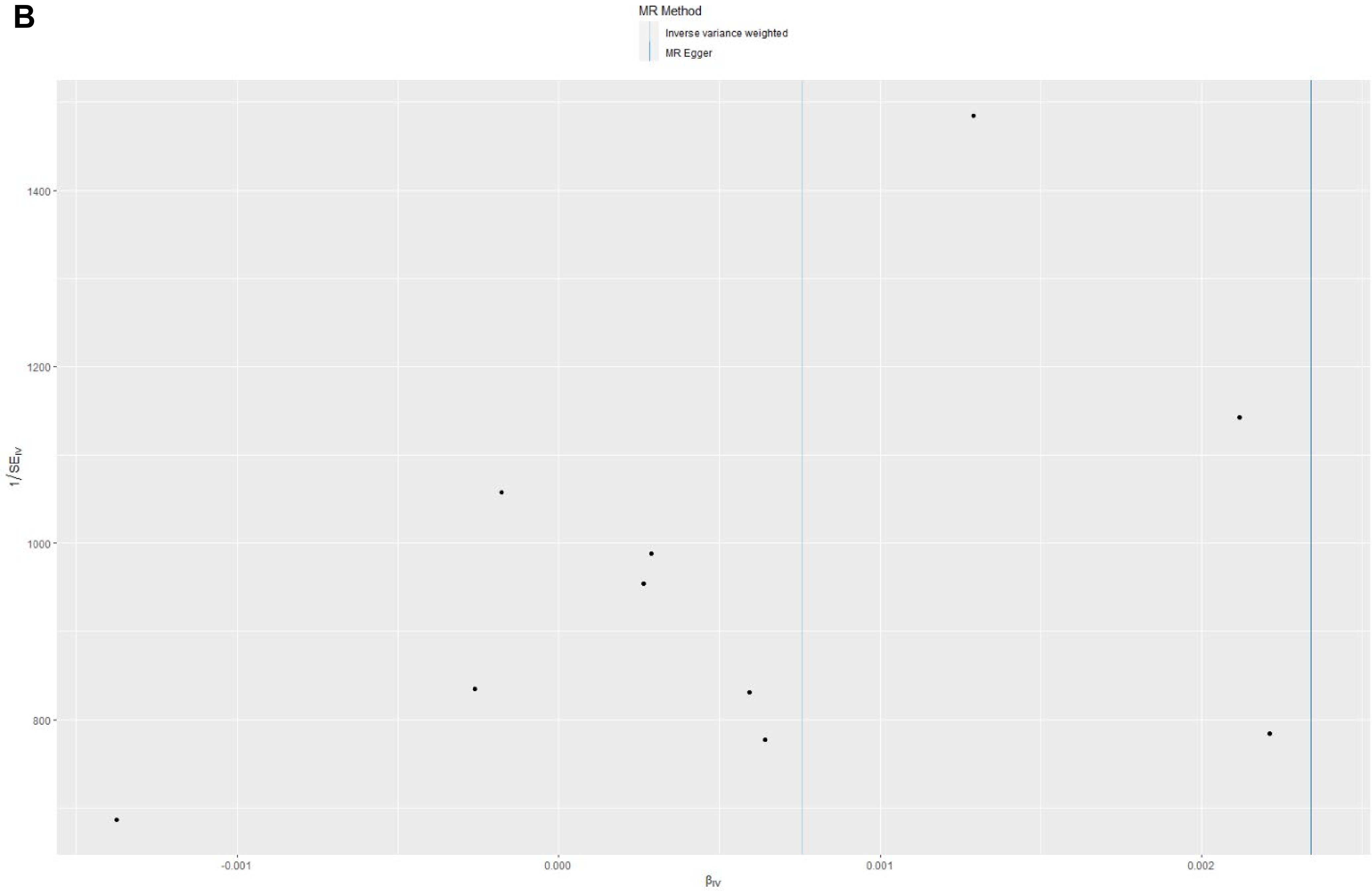

Supplementary Figure 14. Scatter plot (A) and funnel plot (B) of the causal efect of endometriosis on bladder cancer.

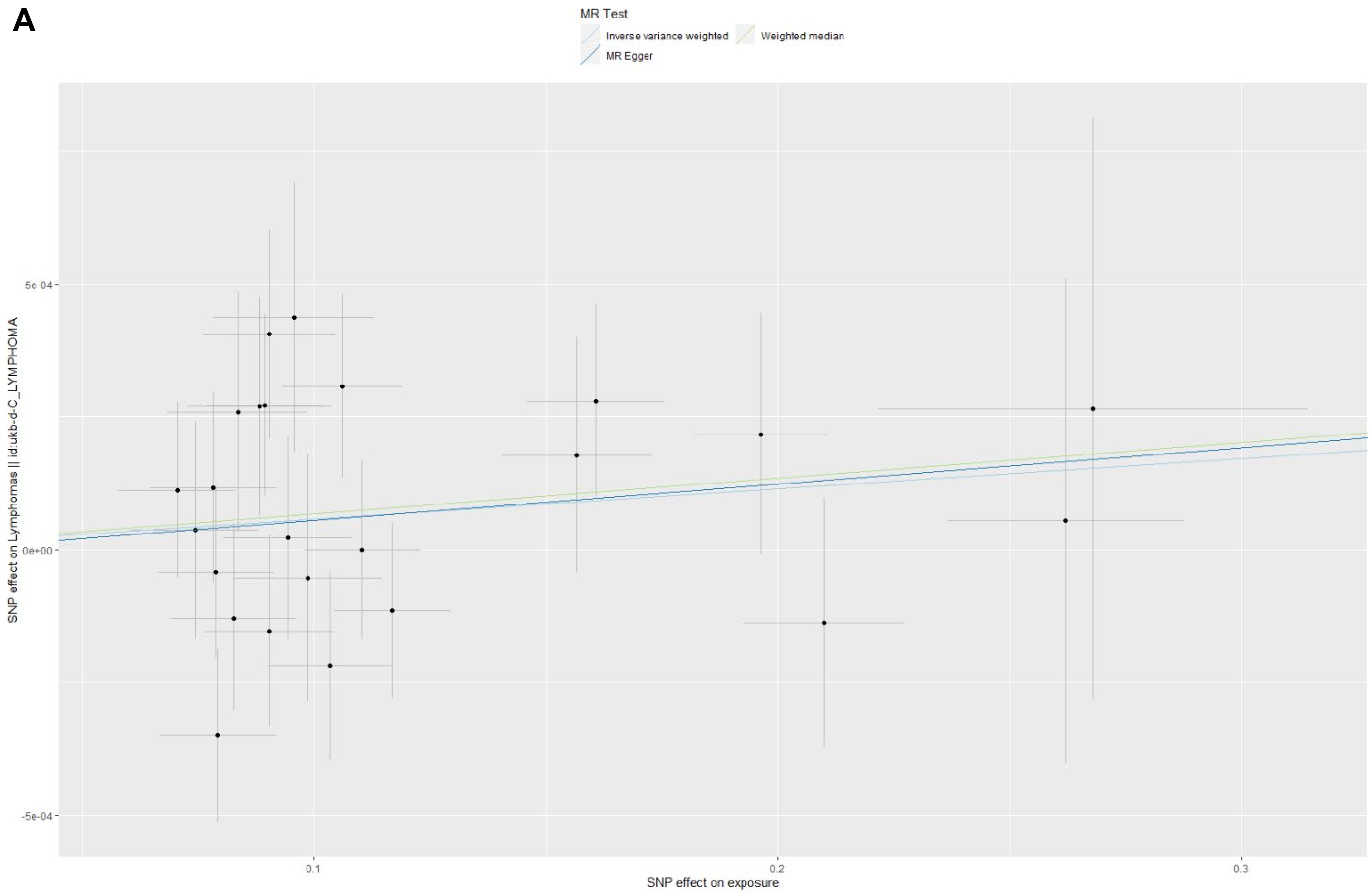

Supplementary Figure 15. Scatter plot (A) and funnel plot (B) of the causal efect of endometriosis on lymphoma.

B

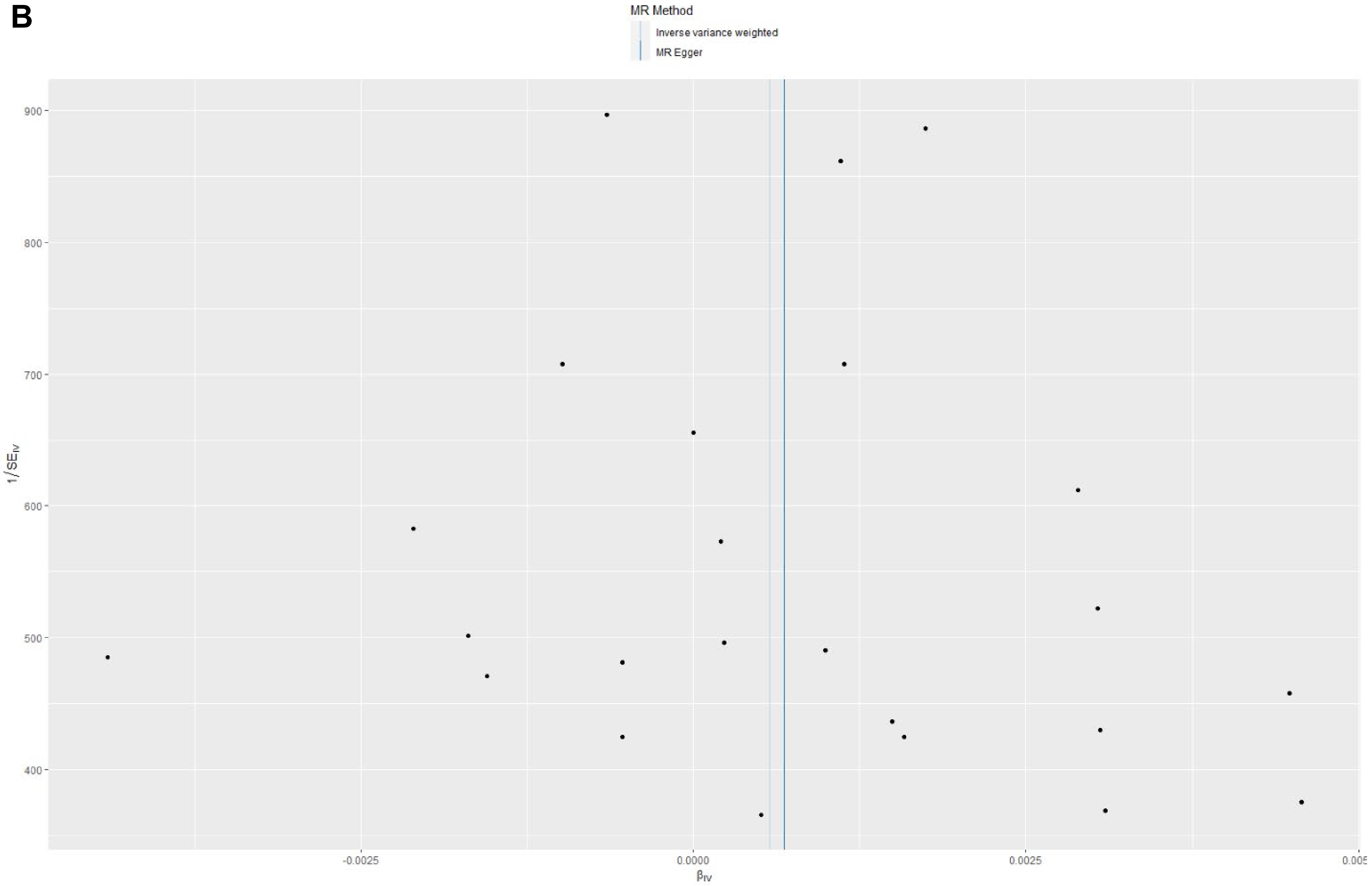

Supplementary Figure 15. Scatter plot (A) and funnel plot (B) of the causal efect of endometriosis on lymphoma.

A

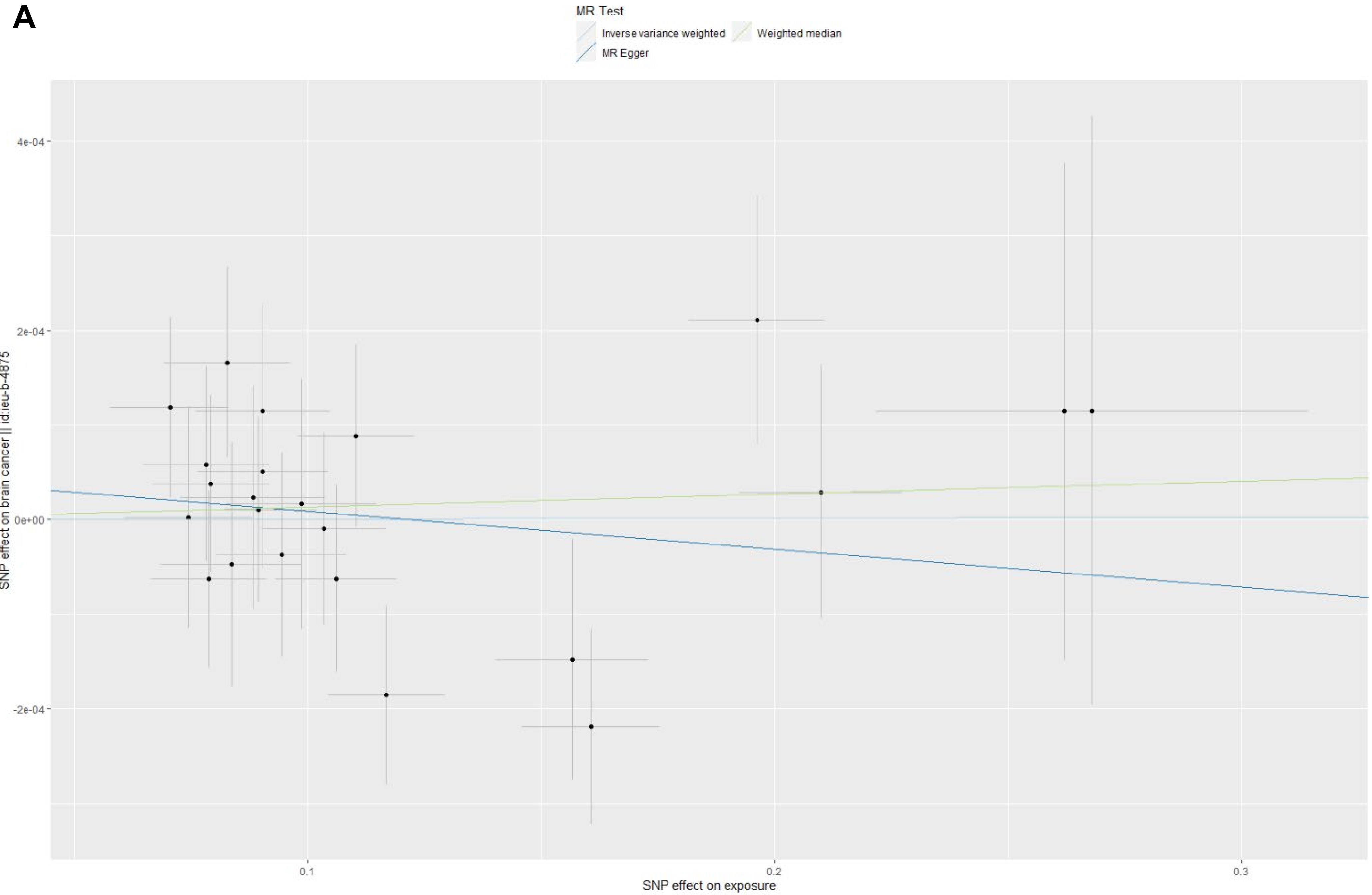

Supplementary Figure 16. Scatter plot (A) and funnel plot (B) of the causal efect of endometriosis on brain cancer.

B

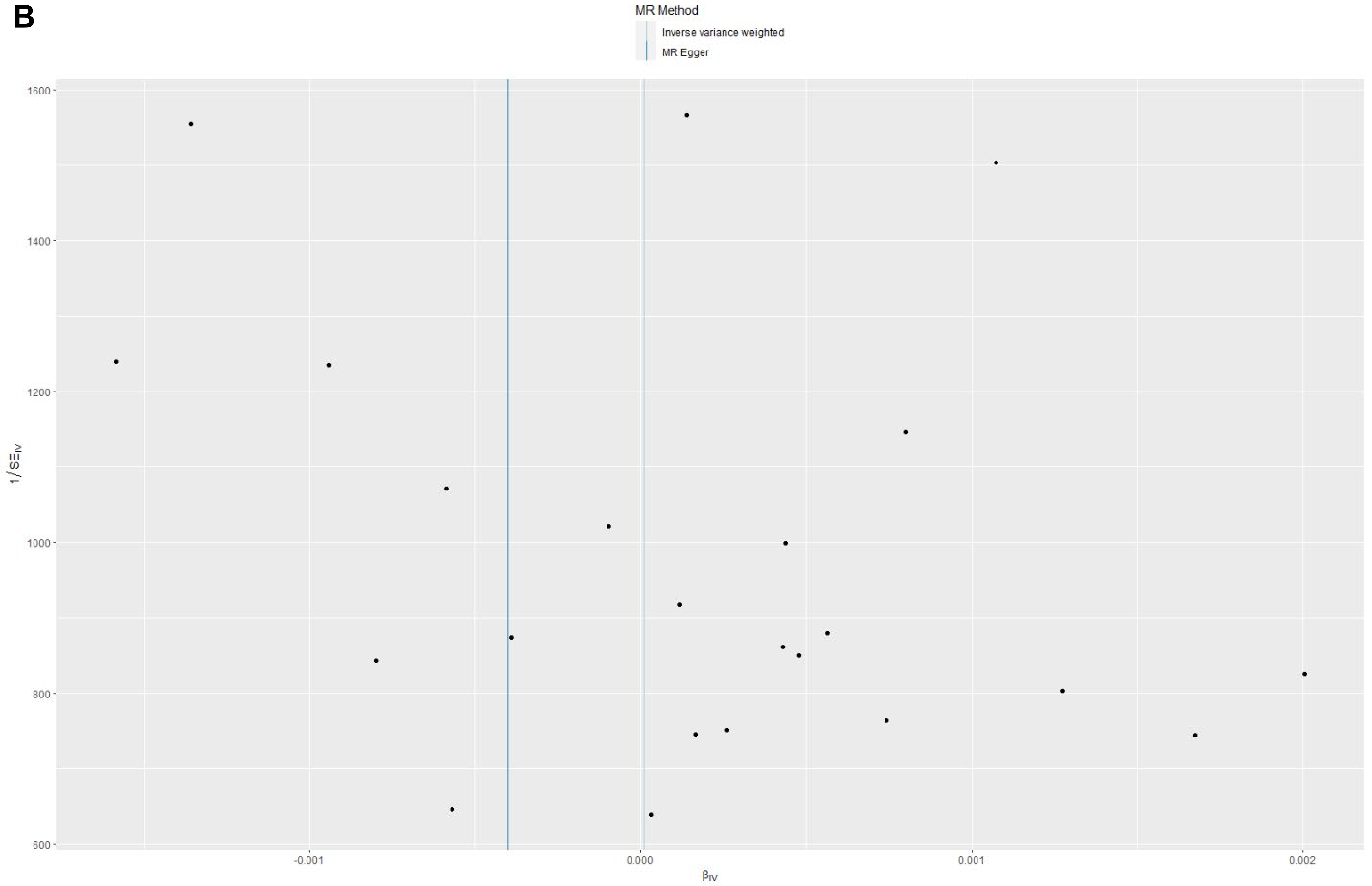

Supplementary Figure 16. Scatter plot (A) and funnel plot (B) of the causal efect of endometriosis on brain cancer.

A

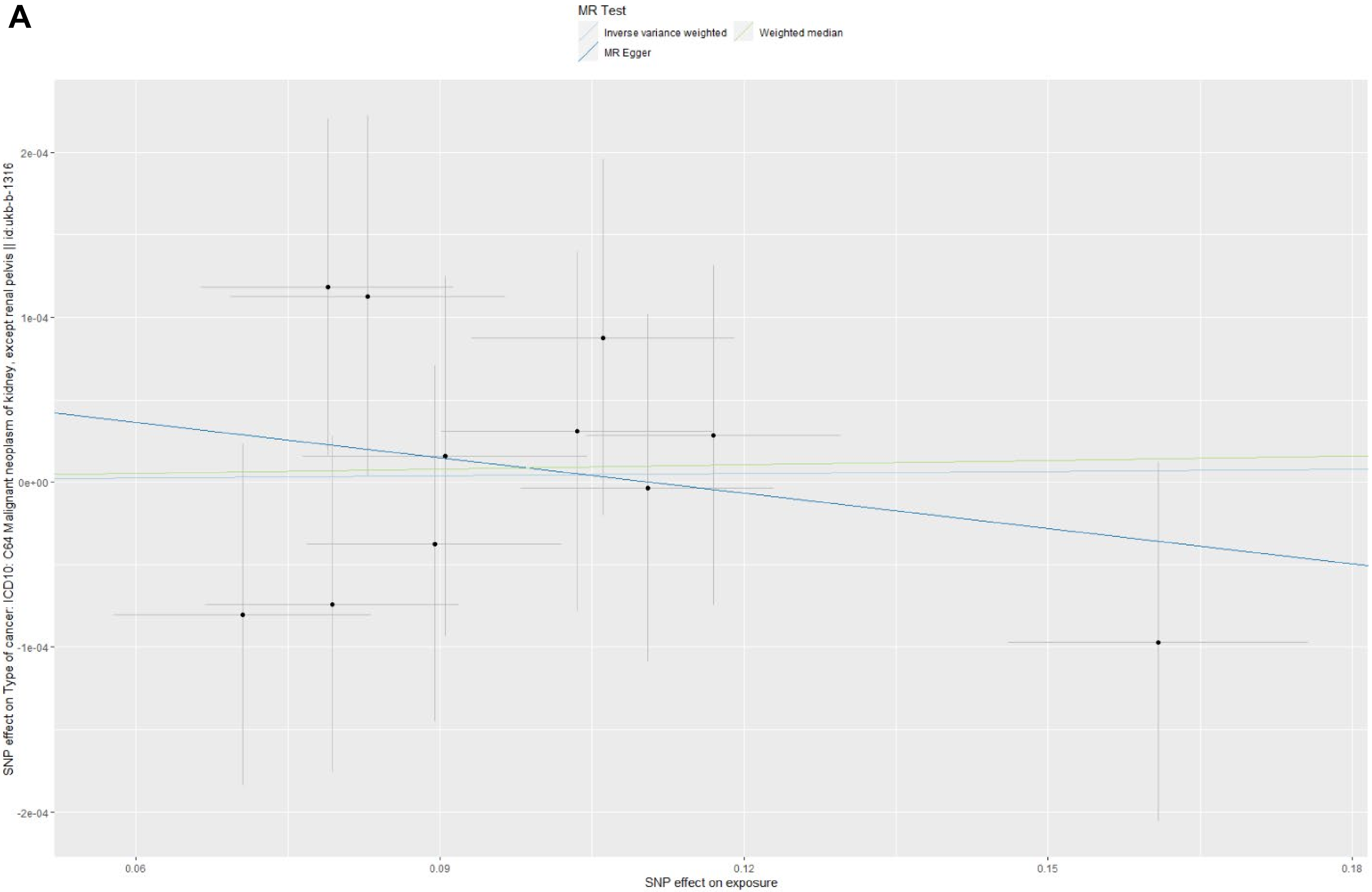

Supplementary Figure 17. Scatter plot (A) and funnel plot (B) of the causal efect of endometriosis on kidney cancer.

B

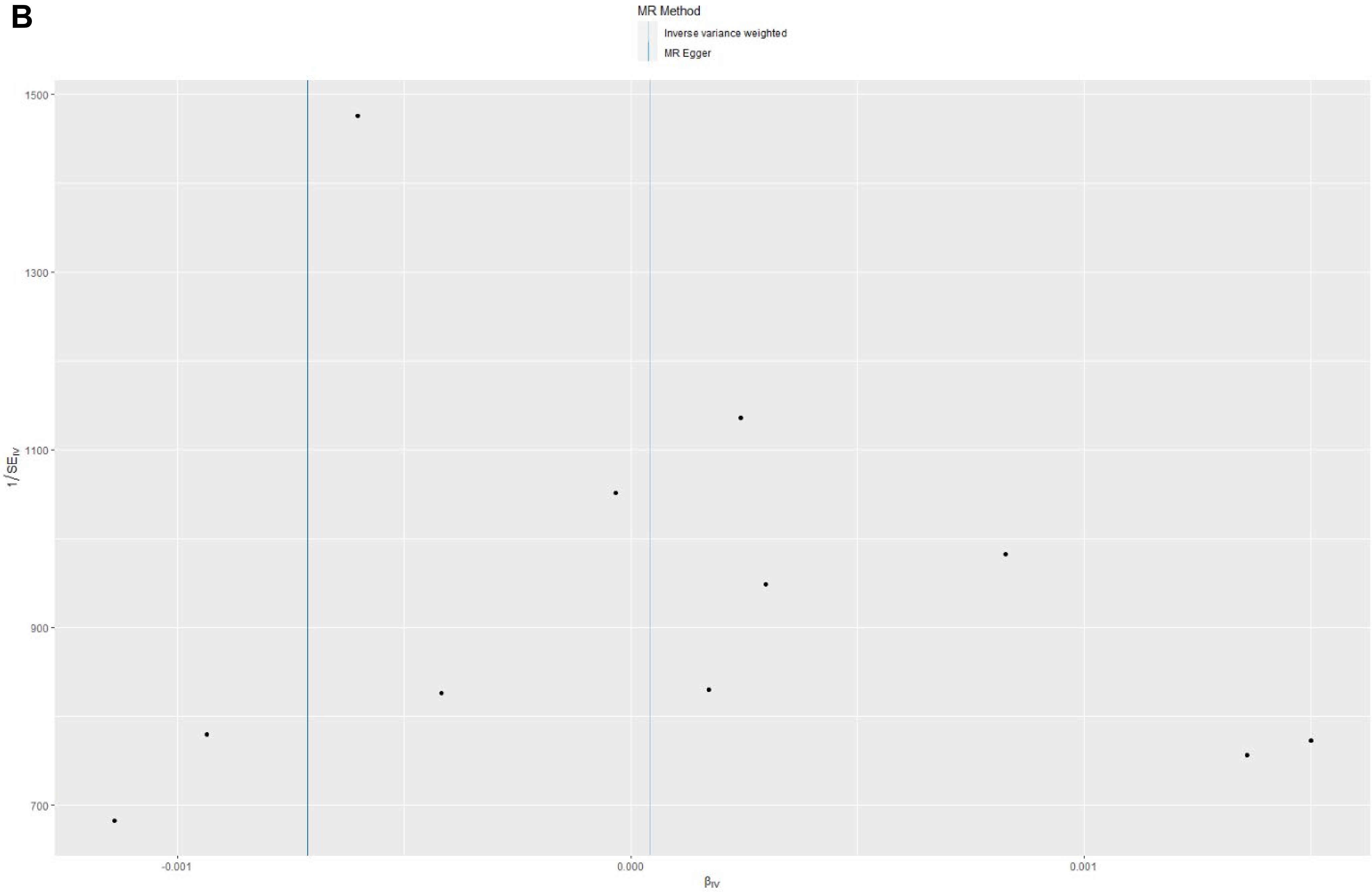

Supplementary Figure 17. Scatter plot (A) and funnel plot (B) of the causal efect of endometriosis on kidney cancer.

A

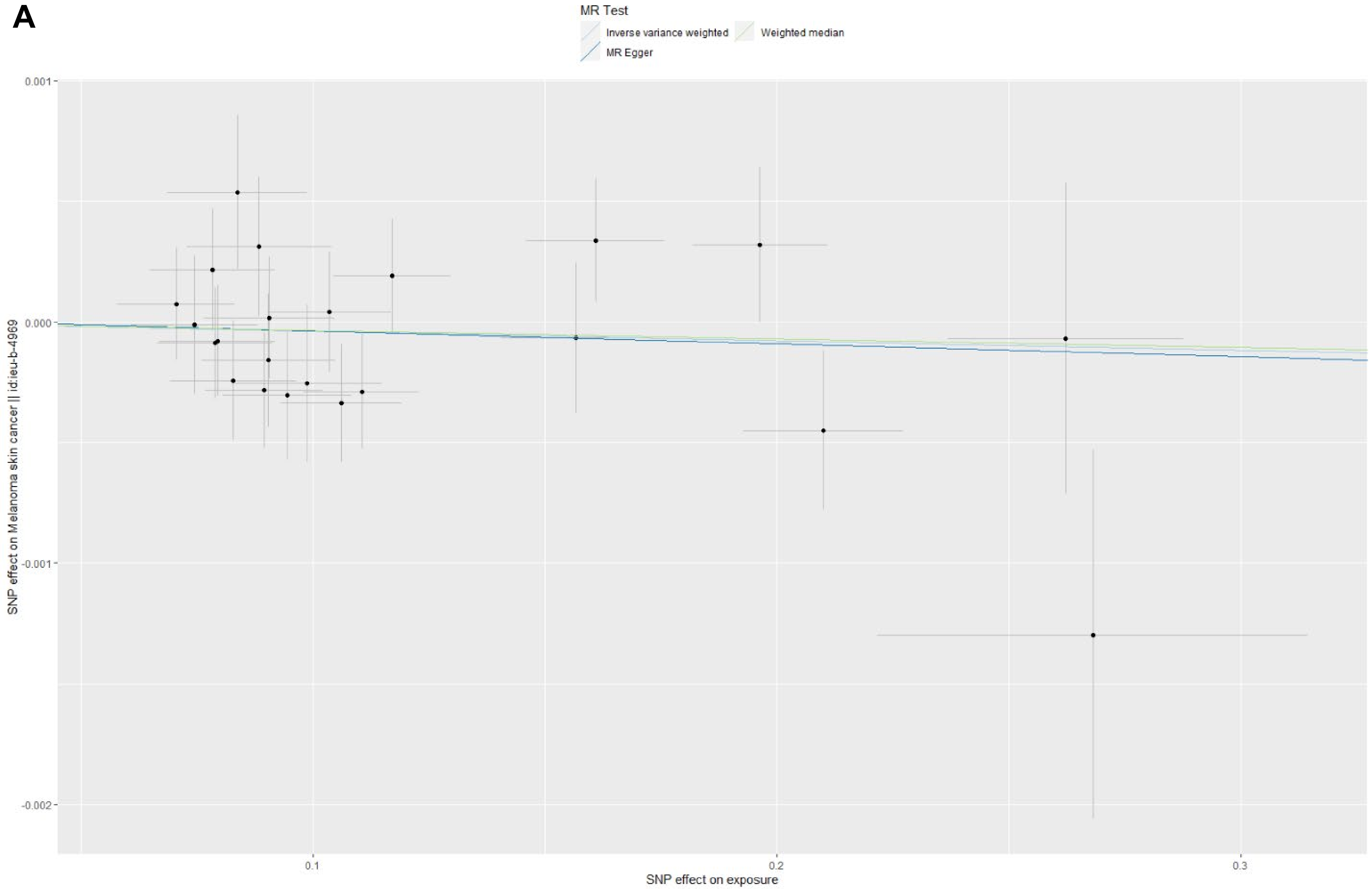

Supplementary Figure 18. Scatter plot (A) and funnel plot (B) of the causal efect of endometriosis on melanoma.

B

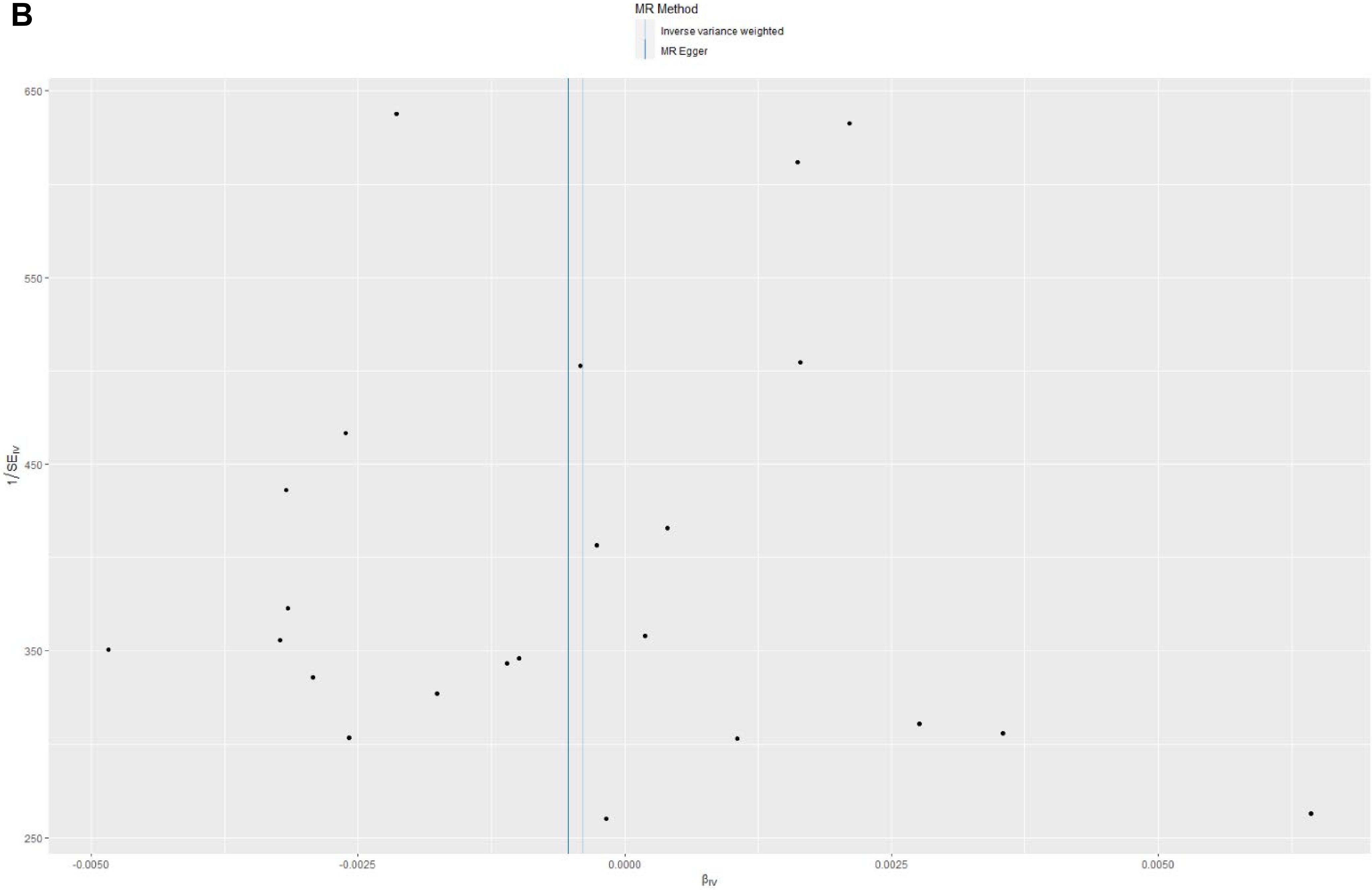

Supplementary Figure 18. Scatter plot (A) and funnel plot (B) of the causal efect of endometriosis on melanoma.

A

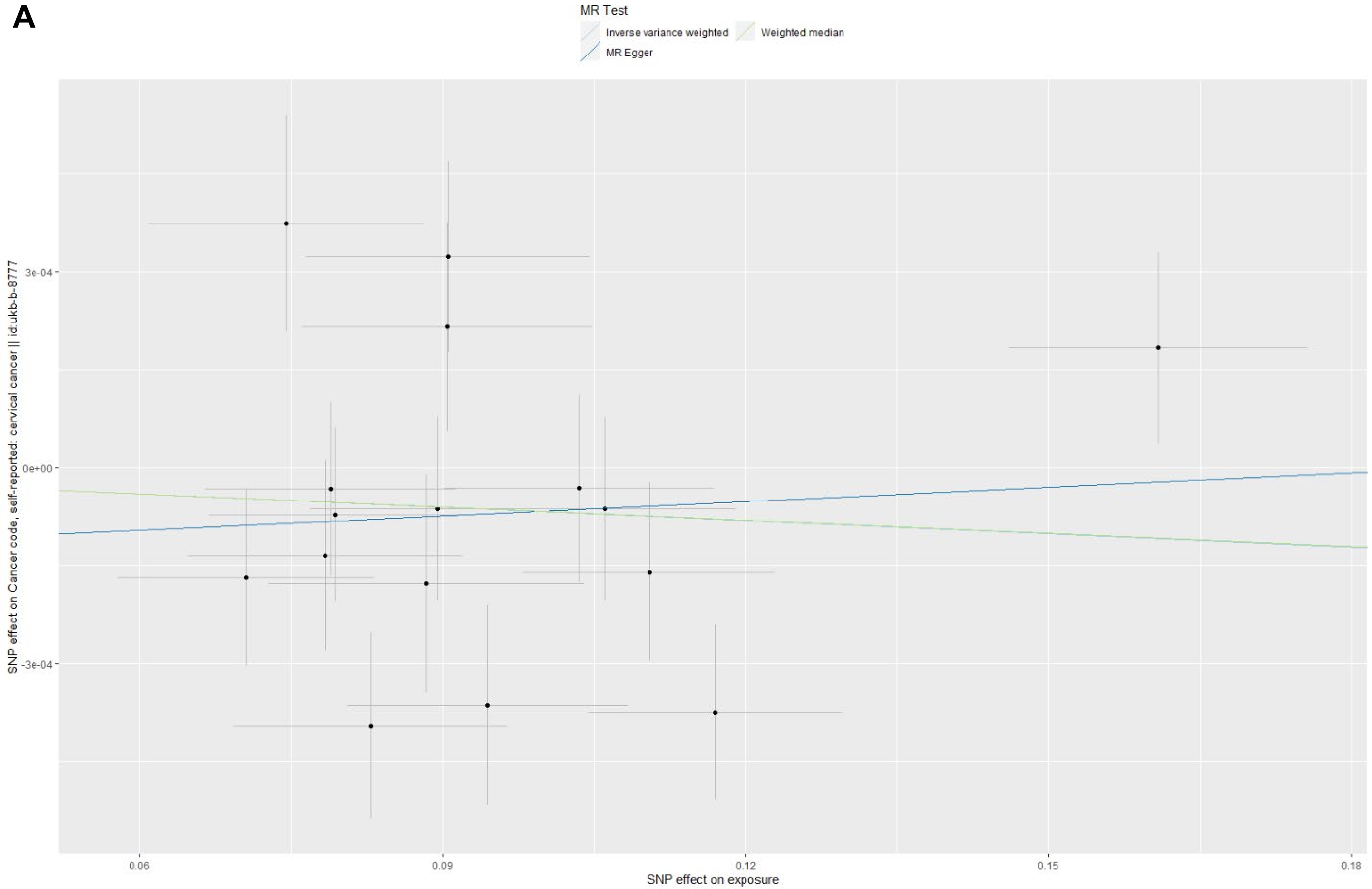

Supplementary Figure 19. Scatter plot (A) and funnel plot (B) of the causal efect of endometriosis on cervical cancer.

B

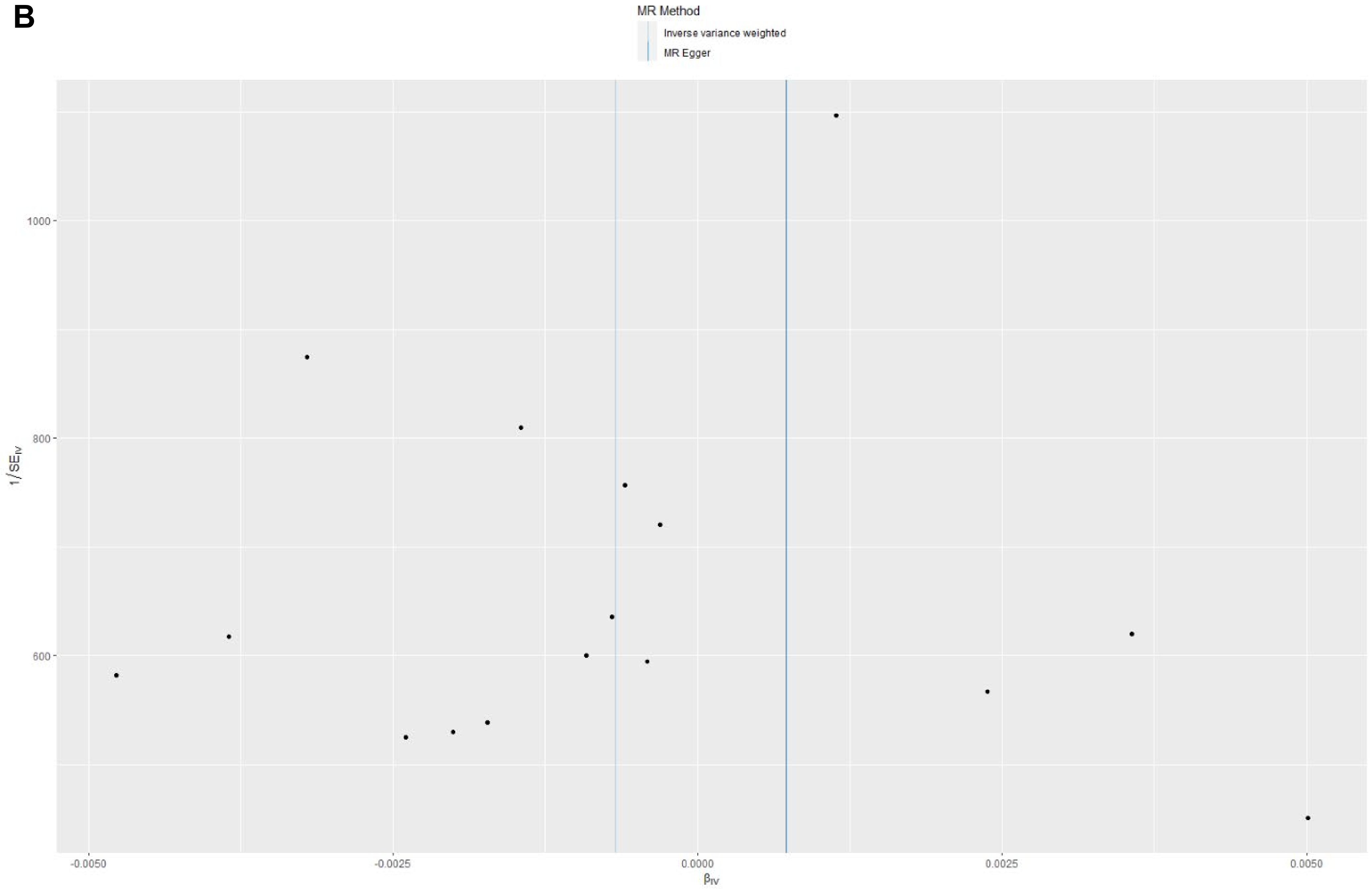

Supplementary Figure 19. Scatter plot (A) and funnel plot (B) of the causal efect of endometriosis on cervical cancer.

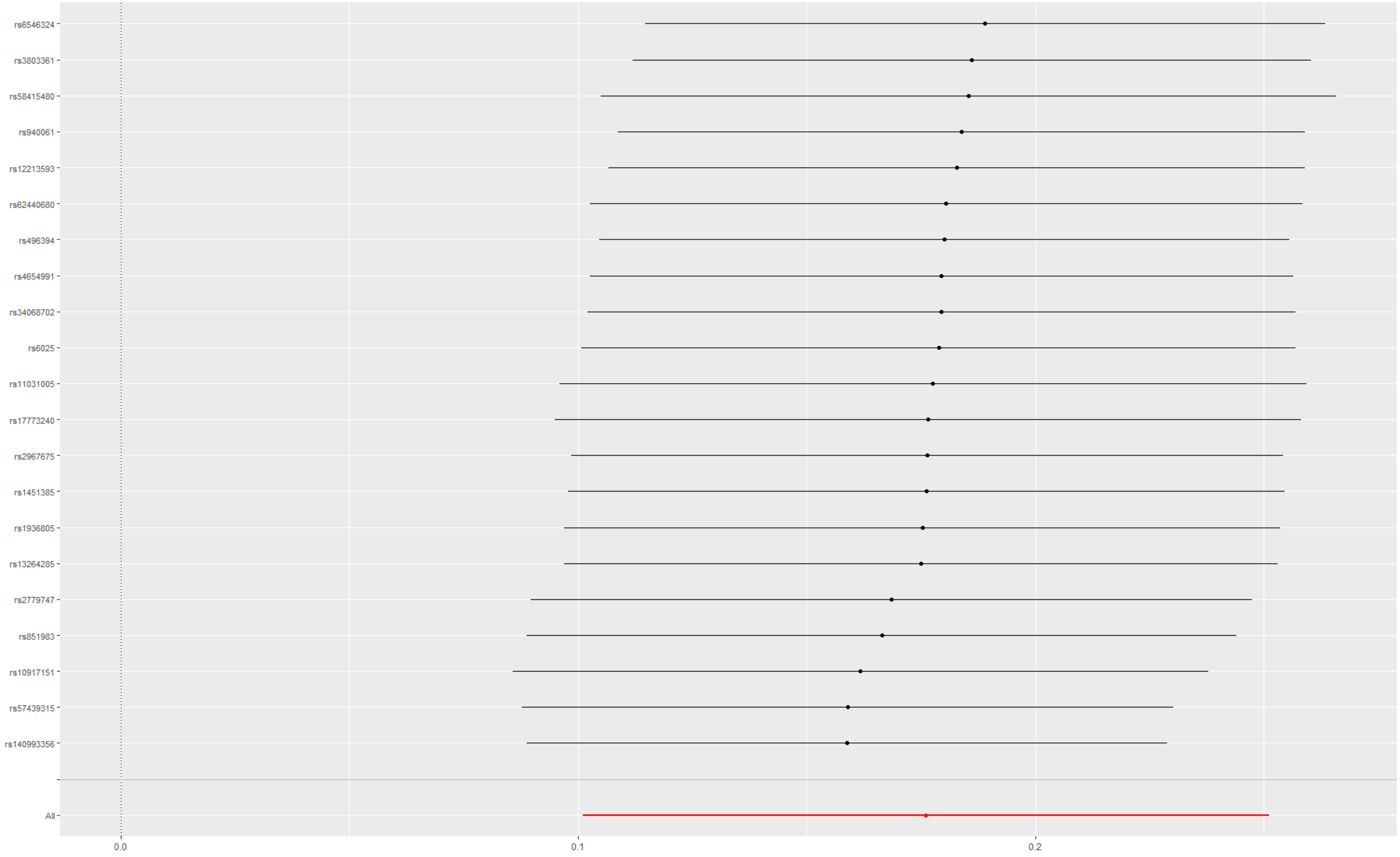

Supplementary Figure 20. Leave-one-out inverse-variance weighted mendelian randomization analyses of endometriosis on ovarian cancer

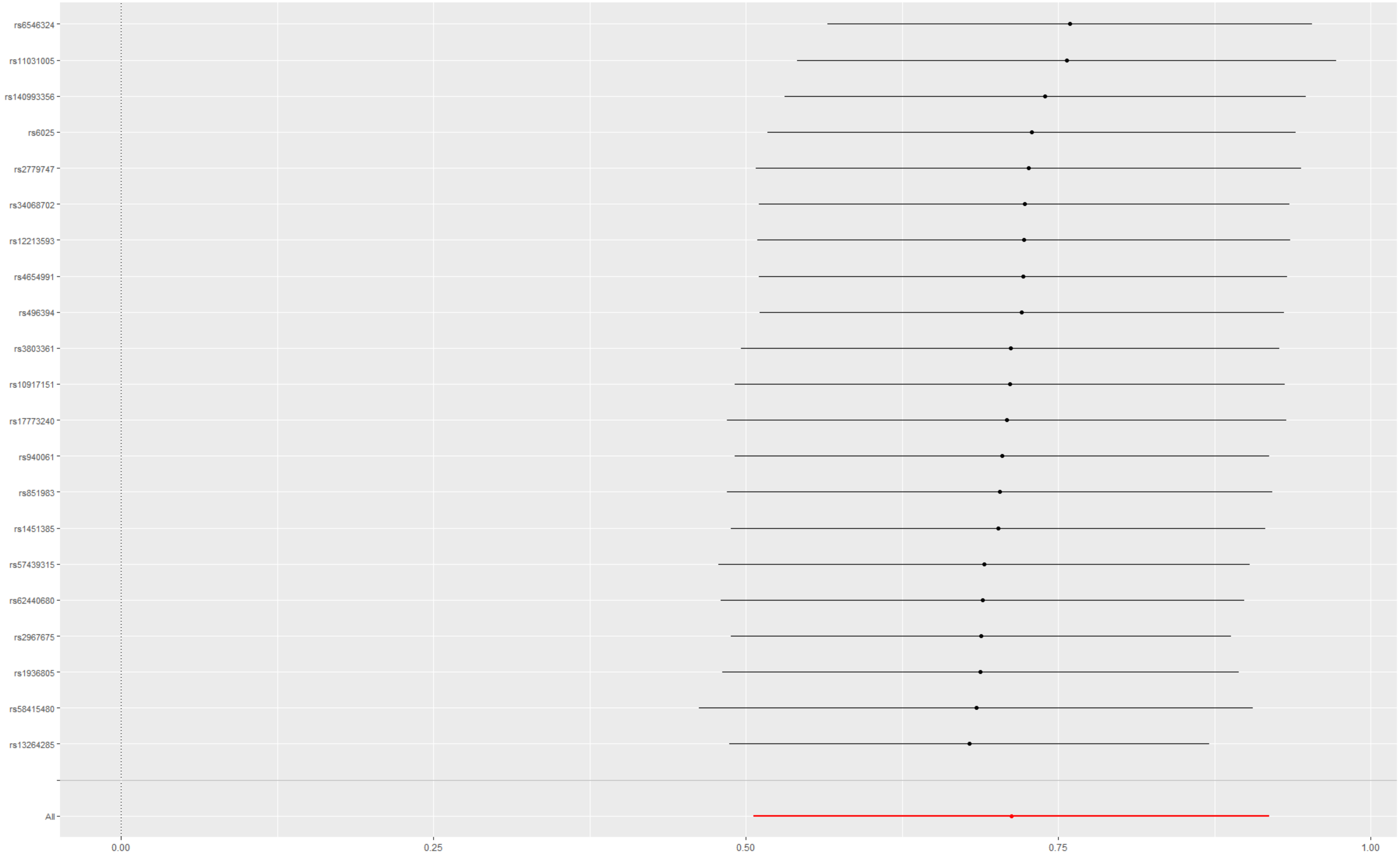

Supplementary Figure 21. Leave-one-out inverse-variance weighted mendelian randomization analyses of endometriosis on clear cell ovarian cancer

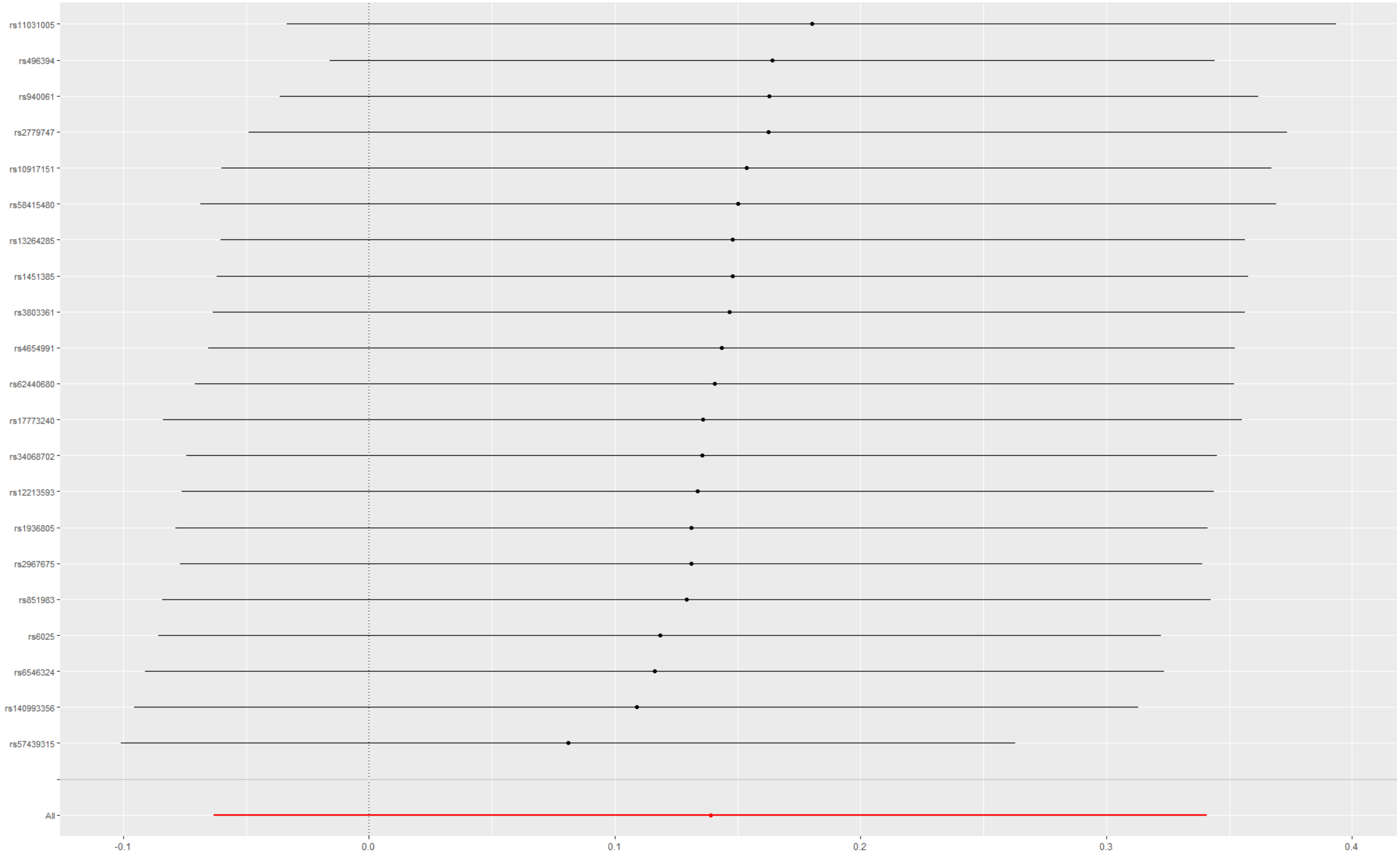

Supplementary Figure 22. Leave-one-out inverse-variance weighted mendelian randomization analyses of endometriosis on invasive mucinous ovarian cancer

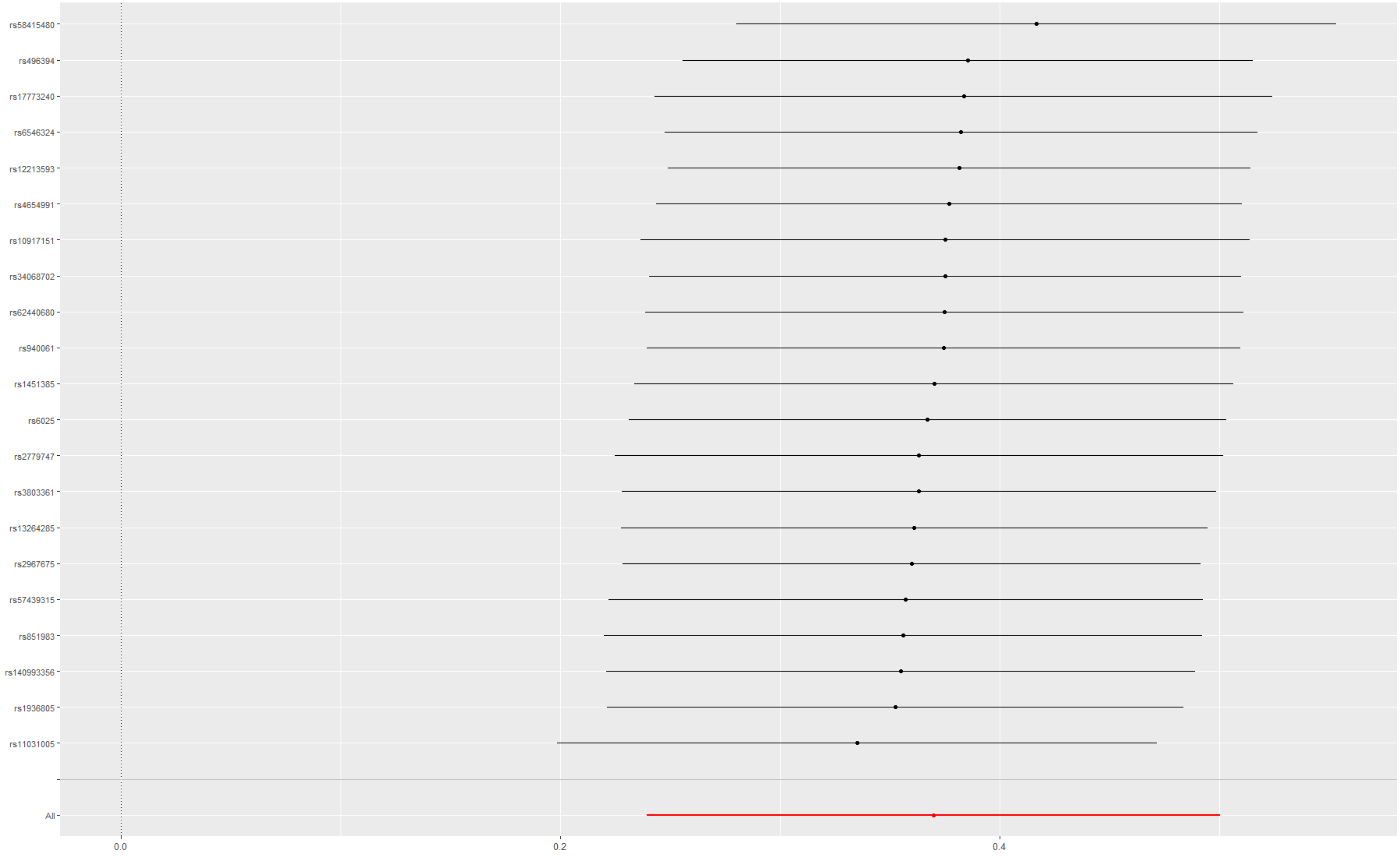

Supplementary Figure 23. Leave-one-out inverse-variance weighted mendelian randomization analyses of endometriosis on endometrioid ovarian cancer

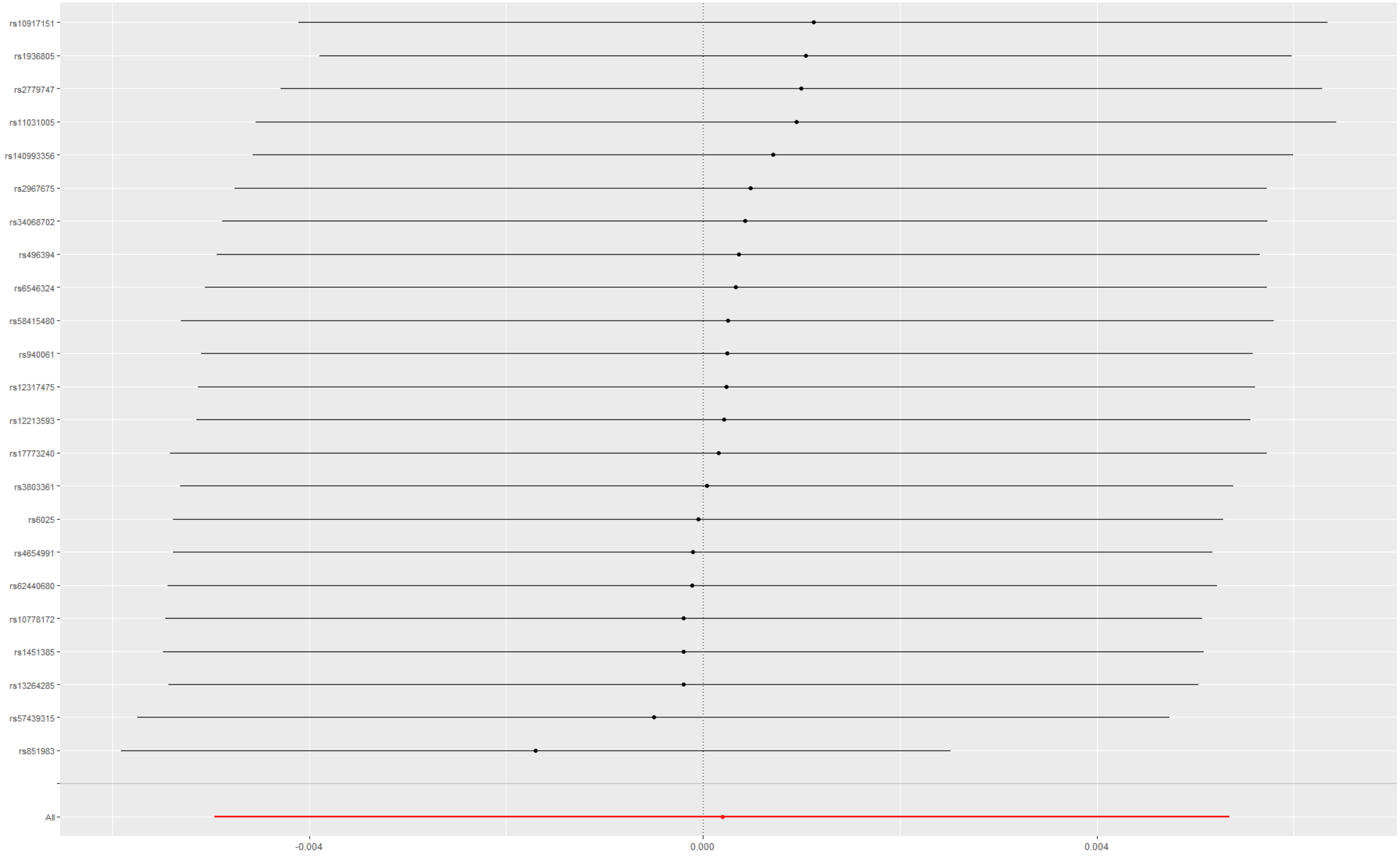

Supplementary Figure 24. Leave-one-out inverse-variance weighted mendelian randomization analyses of endometriosis on breast cancer

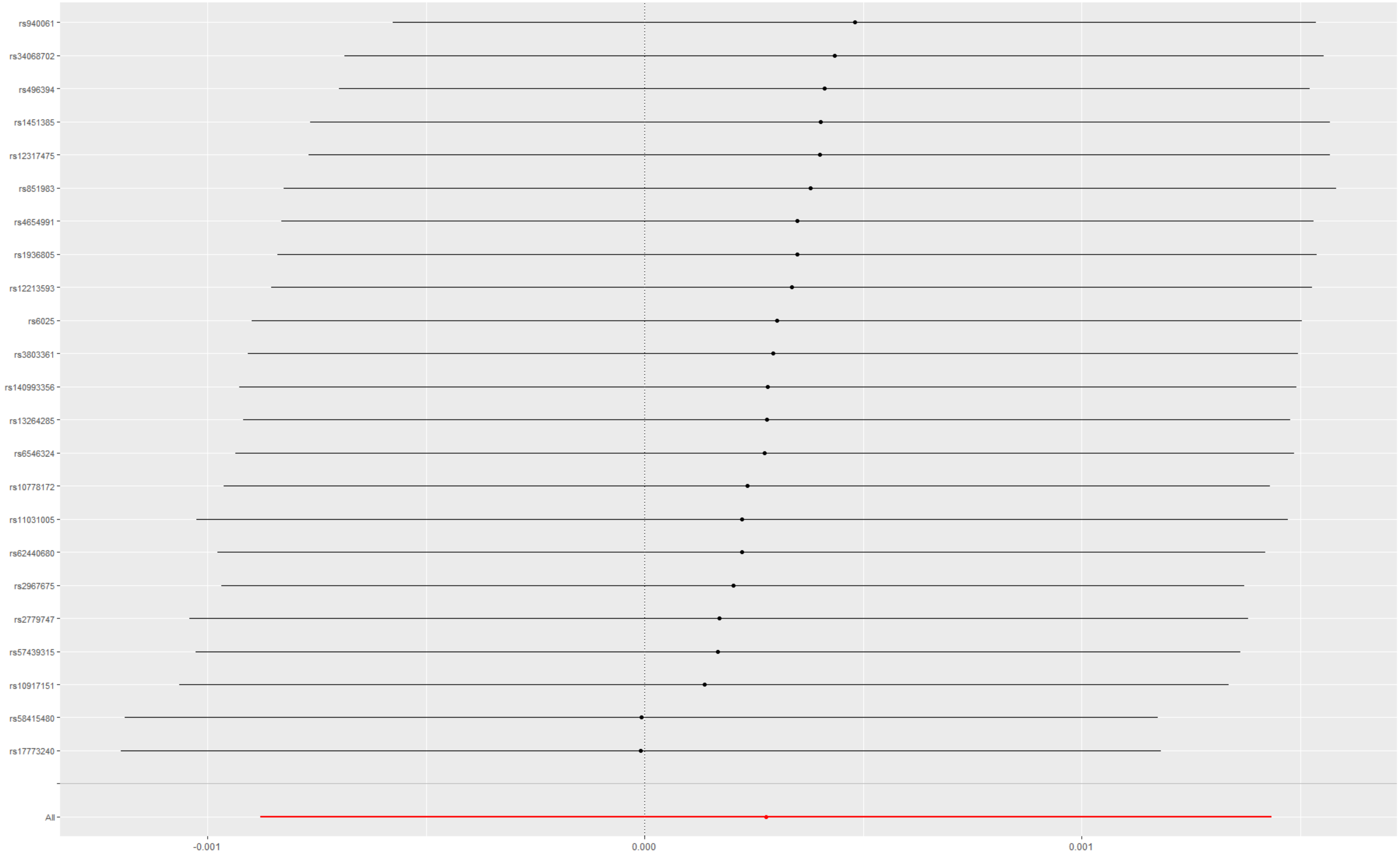

Supplementary Figure 25. Leave-one-out inverse-variance weighted mendelian randomization analyses of endometriosis on lung cancer

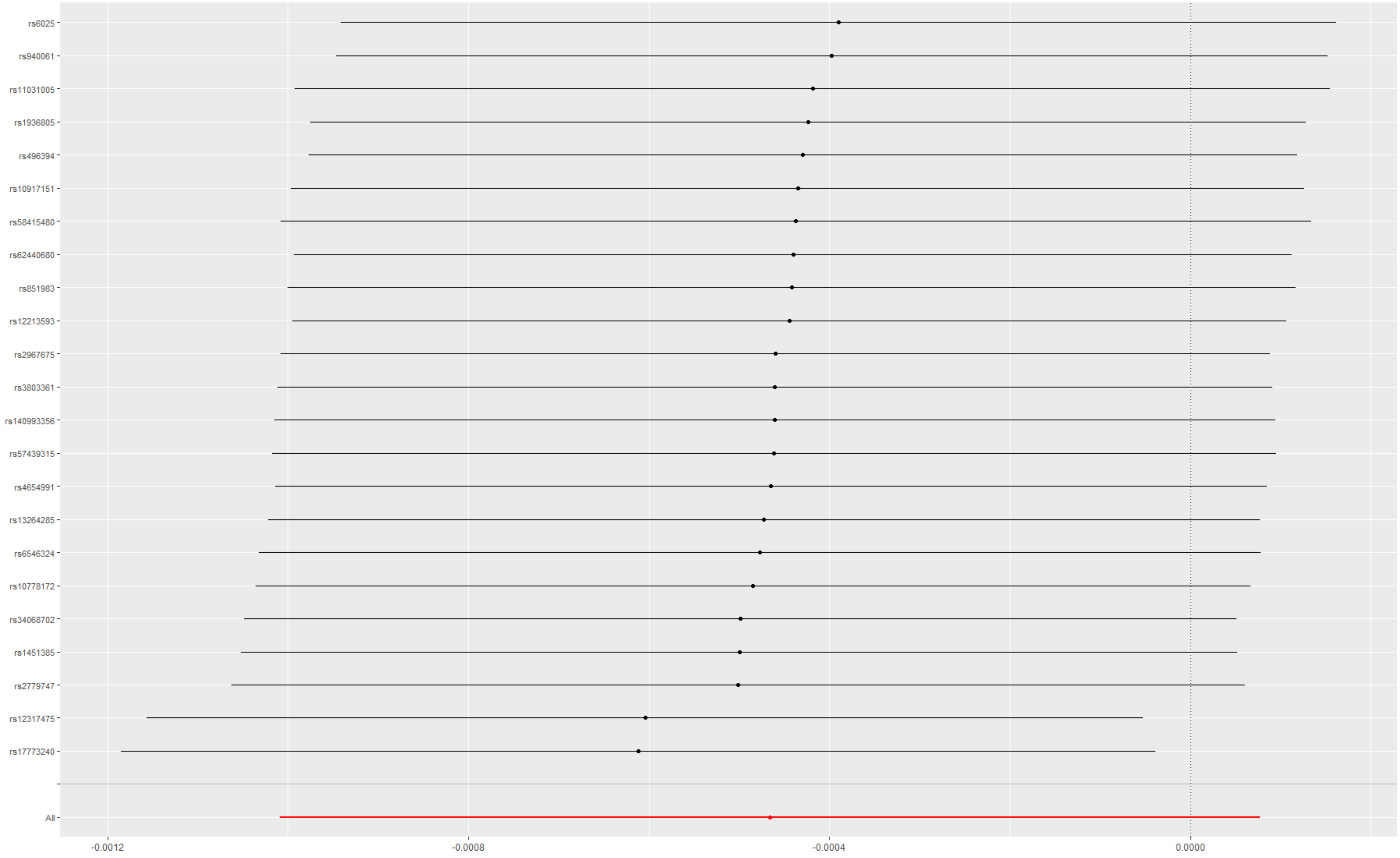

Supplementary Figure 26. Leave-one-out inverse-variance weighted mendelian randomization analyses of endometriosis on head and neck cancer

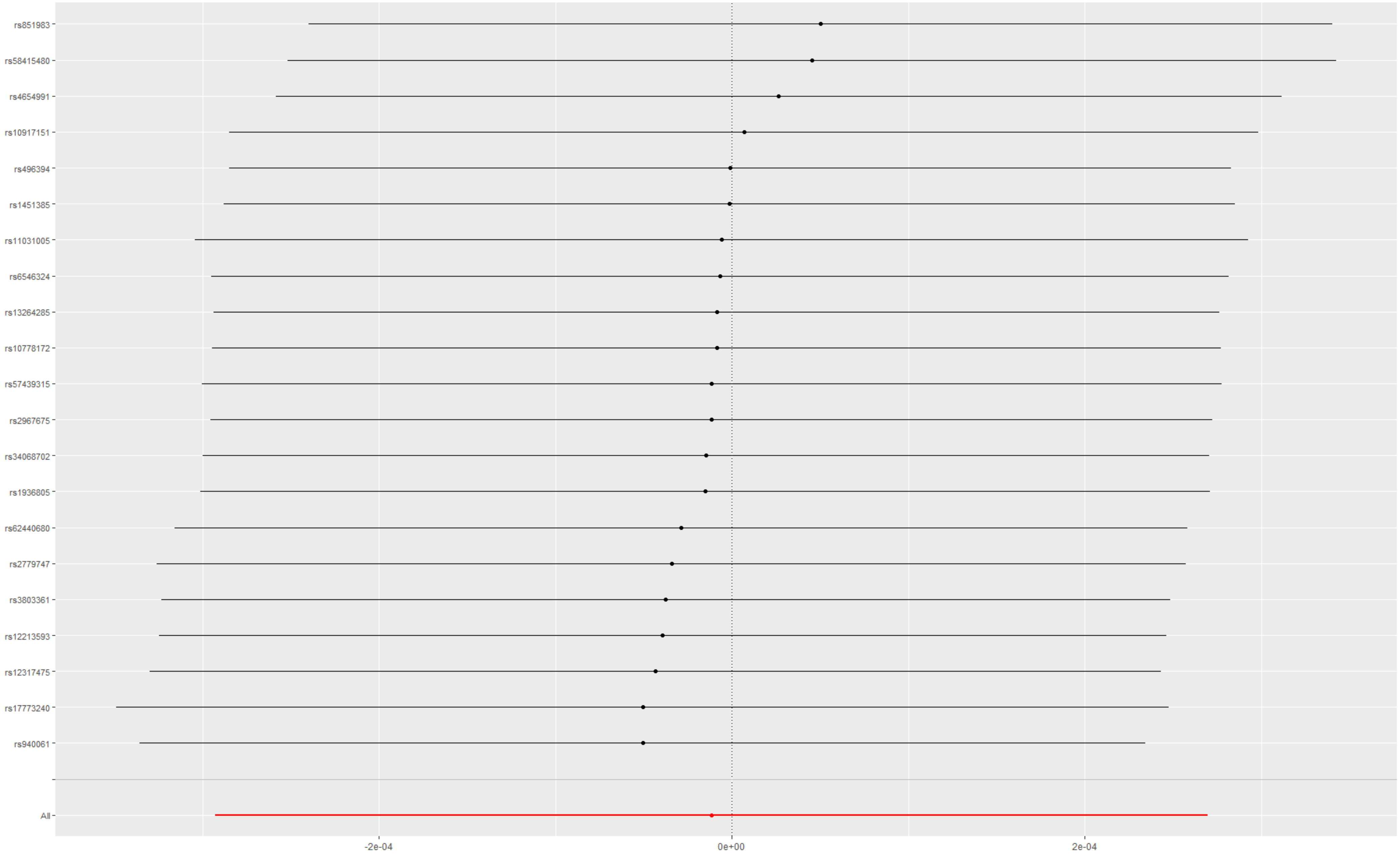

Supplementary Figure 27. Leave-one-out inverse-variance weighted mendelian randomization analyses of endometriosis on laryngeal cancer

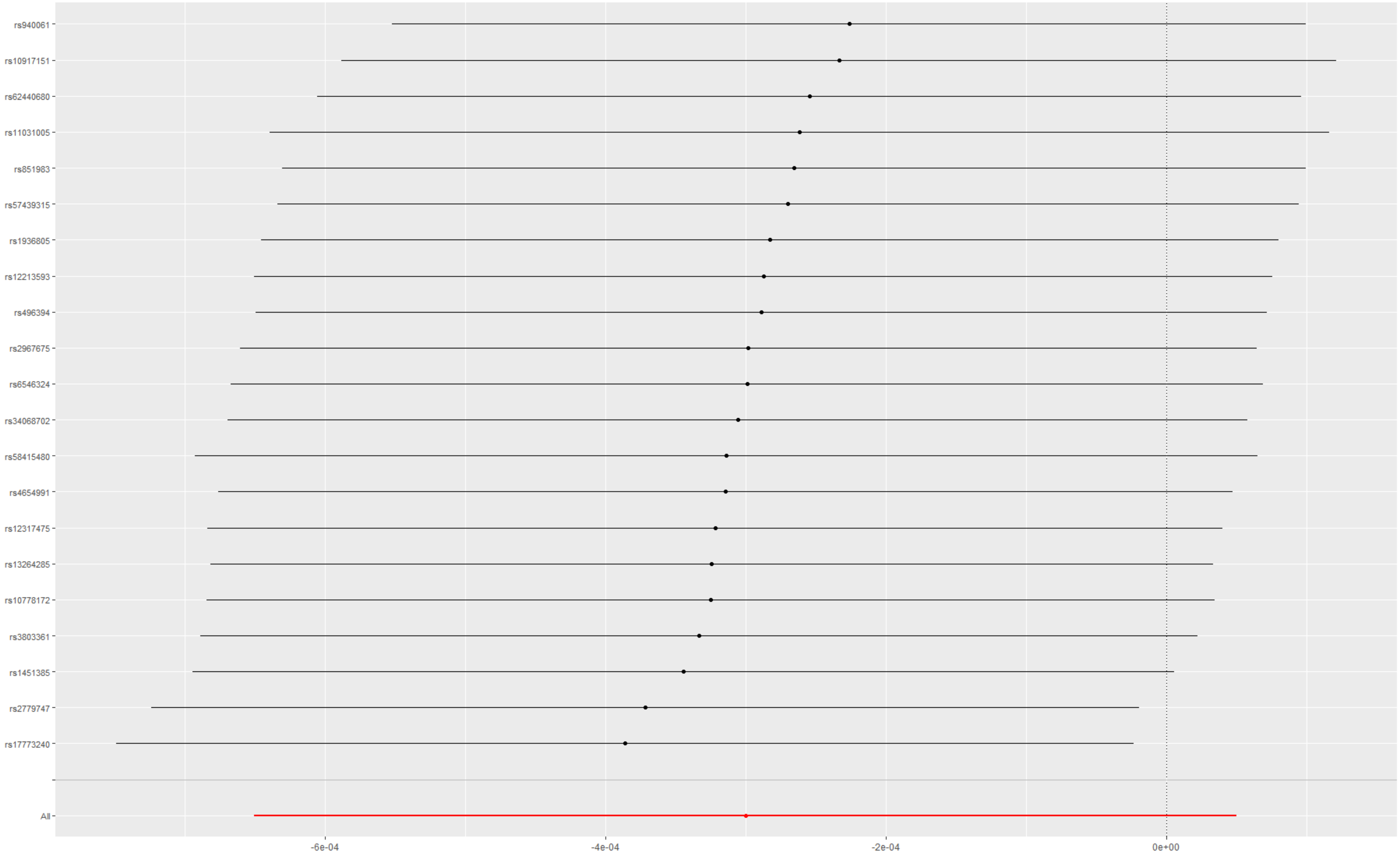

Supplementary Figure 28. Leave-one-out inverse-variance weighted mendelian randomization analyses of endometriosis on oral cavity cancer

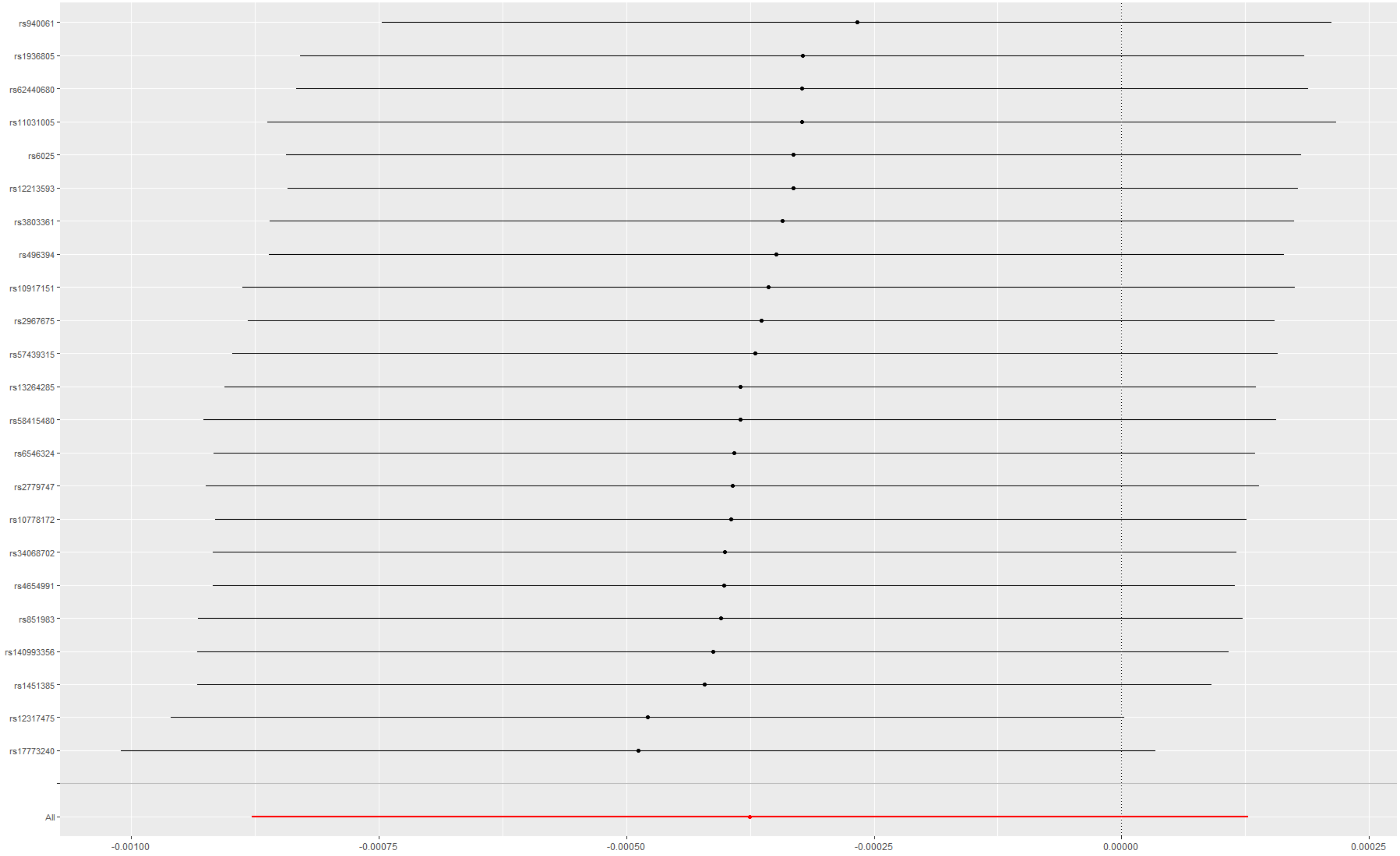

Supplementary Figure 29. Leave-one-out inverse-variance weighted mendelian randomization analyses of endometriosis on oral and oropharyngeal cancer

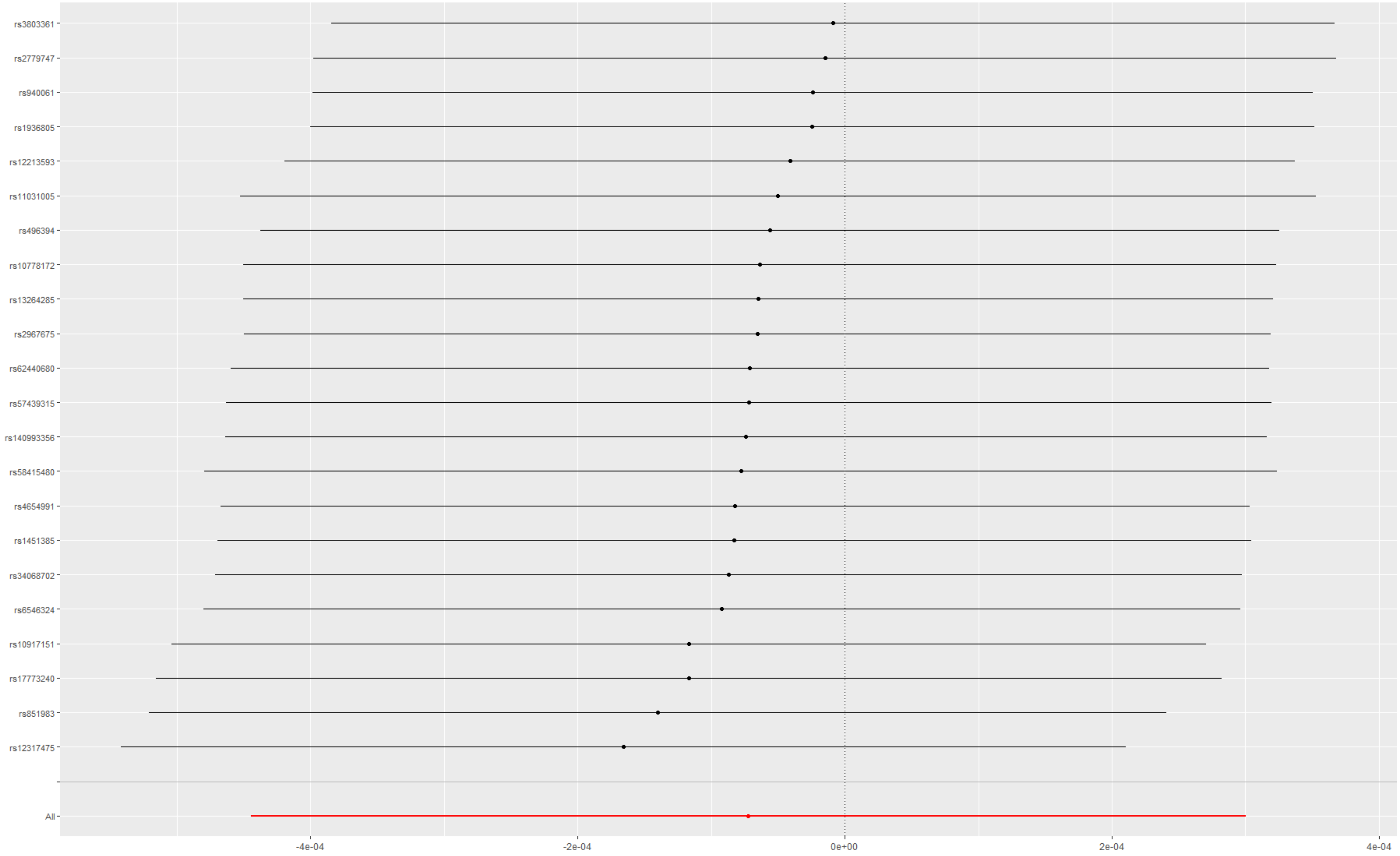

Supplementary Figure 30. Leave-one-out inverse-variance weighted mendelian randomization analyses of endometriosis on oropharyngeal cancer

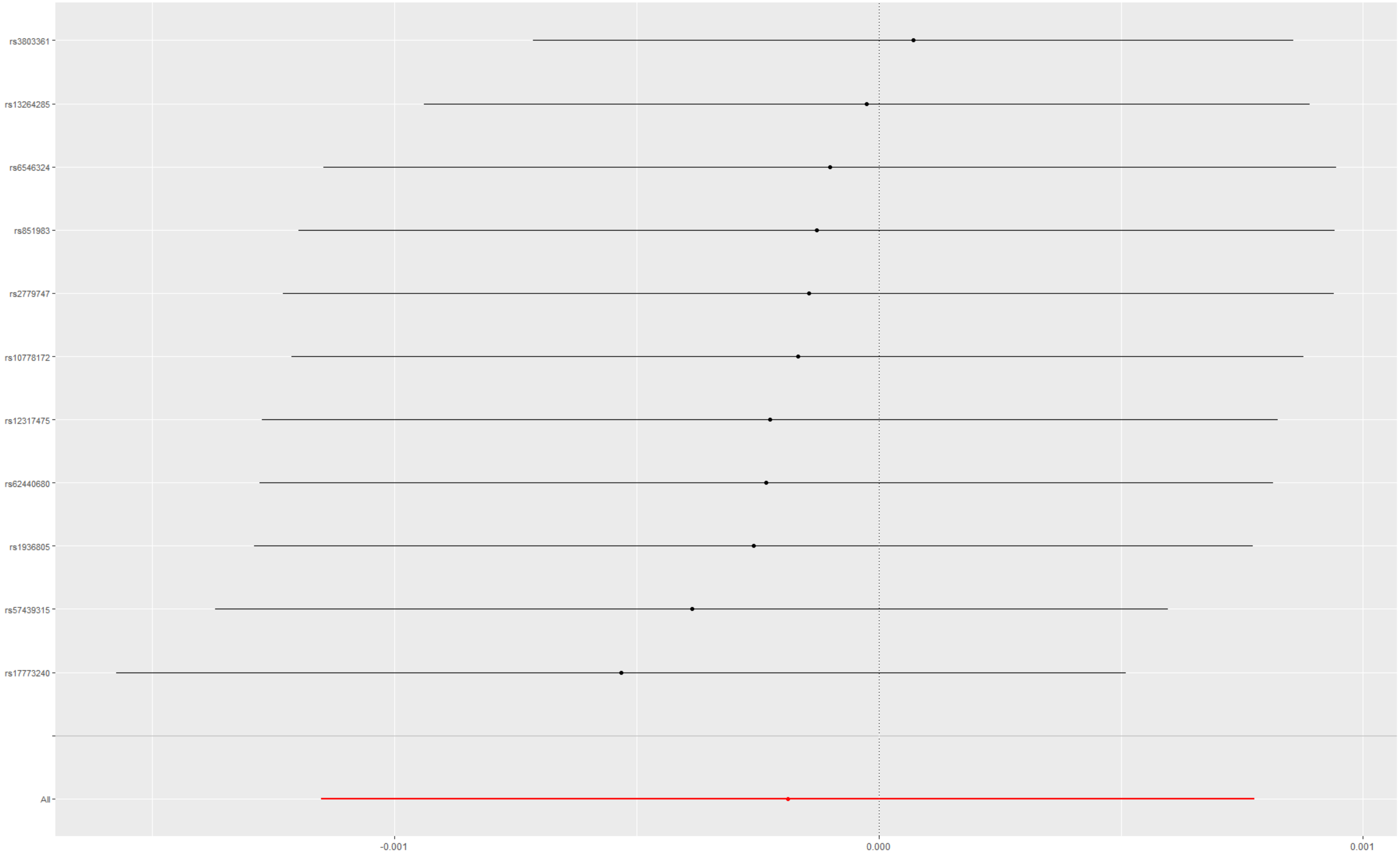

Supplementary Figure 31. Leave-one-out inverse-variance weighted mendelian randomization analyses of endometriosis on endometrial cancer

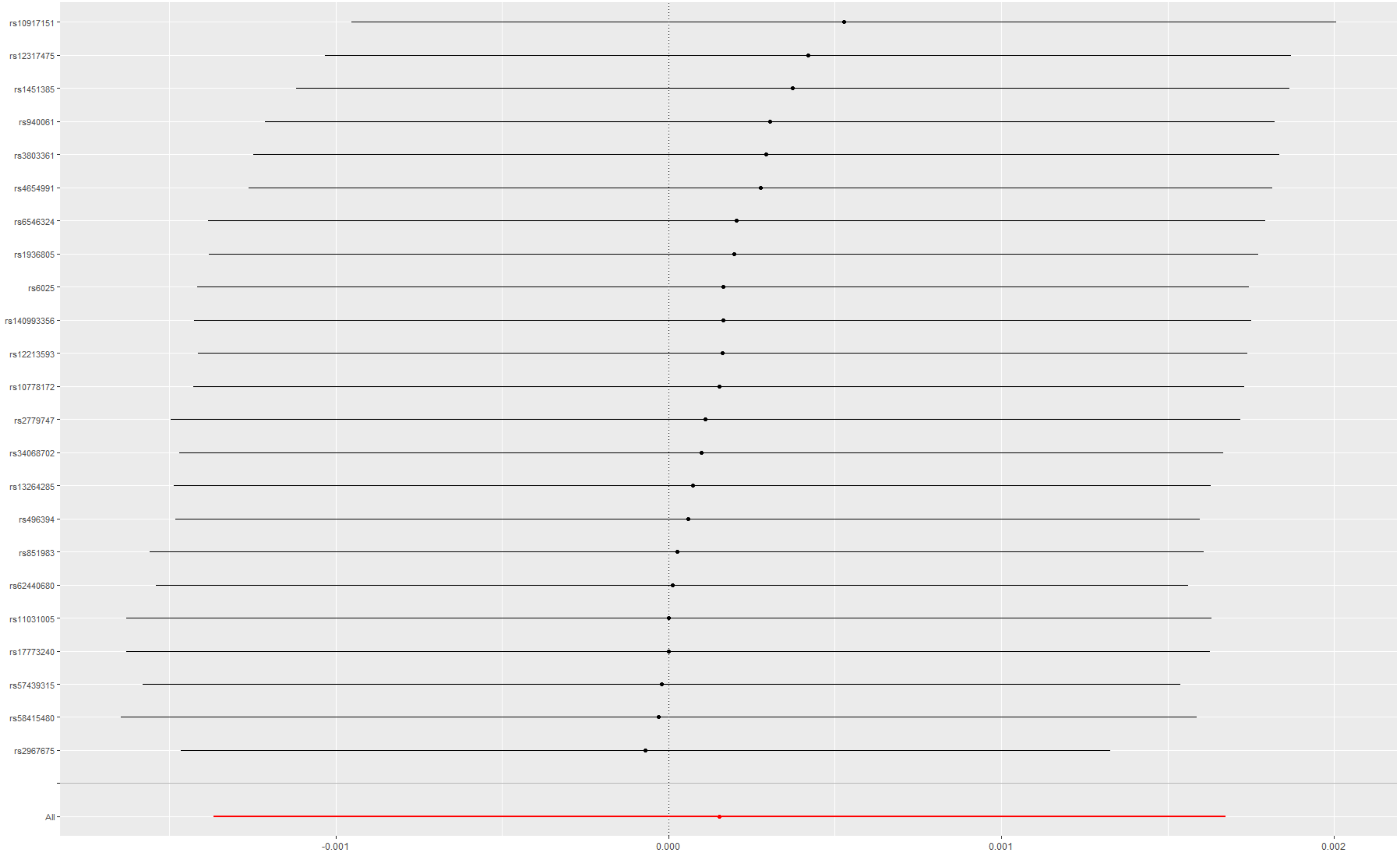

Supplementary Figure 32. Leave-one-out inverse-variance weighted mendelian randomization analyses of endometriosis on colorectal cancer

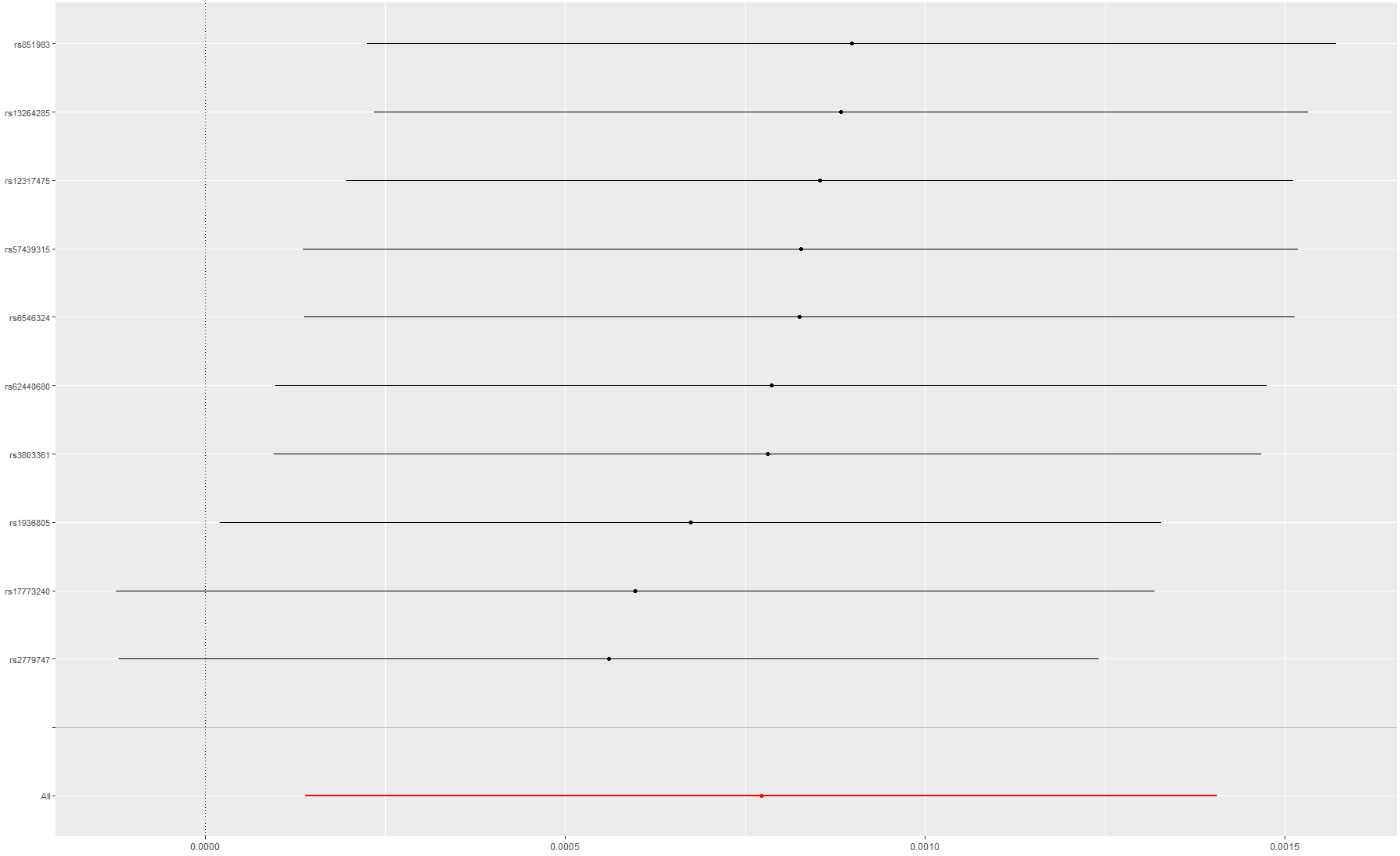

Supplementary Figure 33. Leave-one-out inverse-variance weighted mendelian randomization analyses of endometriosis on bladder cancer

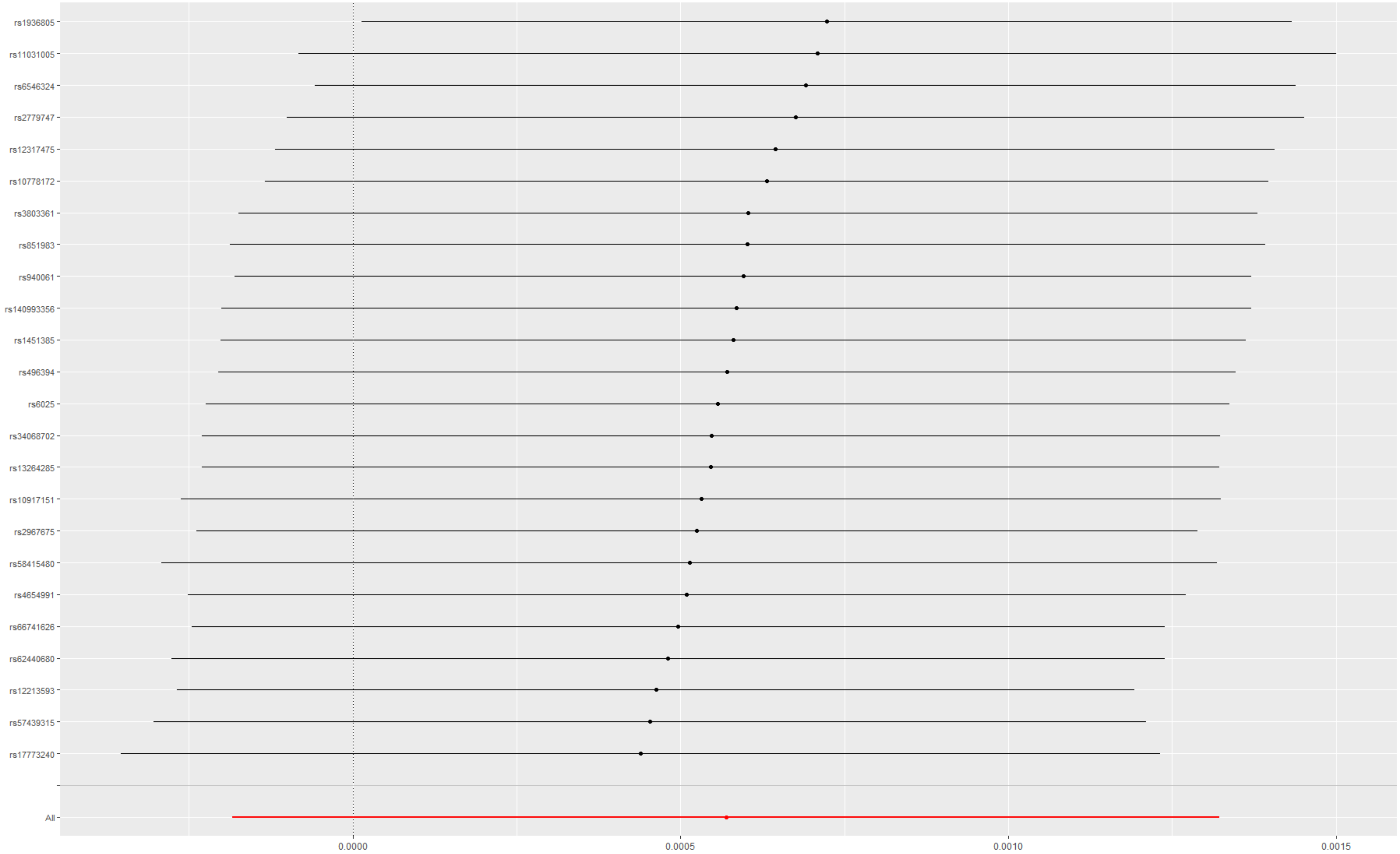

Supplementary Figure 34. Leave-one-out inverse-variance weighted mendelian randomization analyses of endometriosis on lymphoma

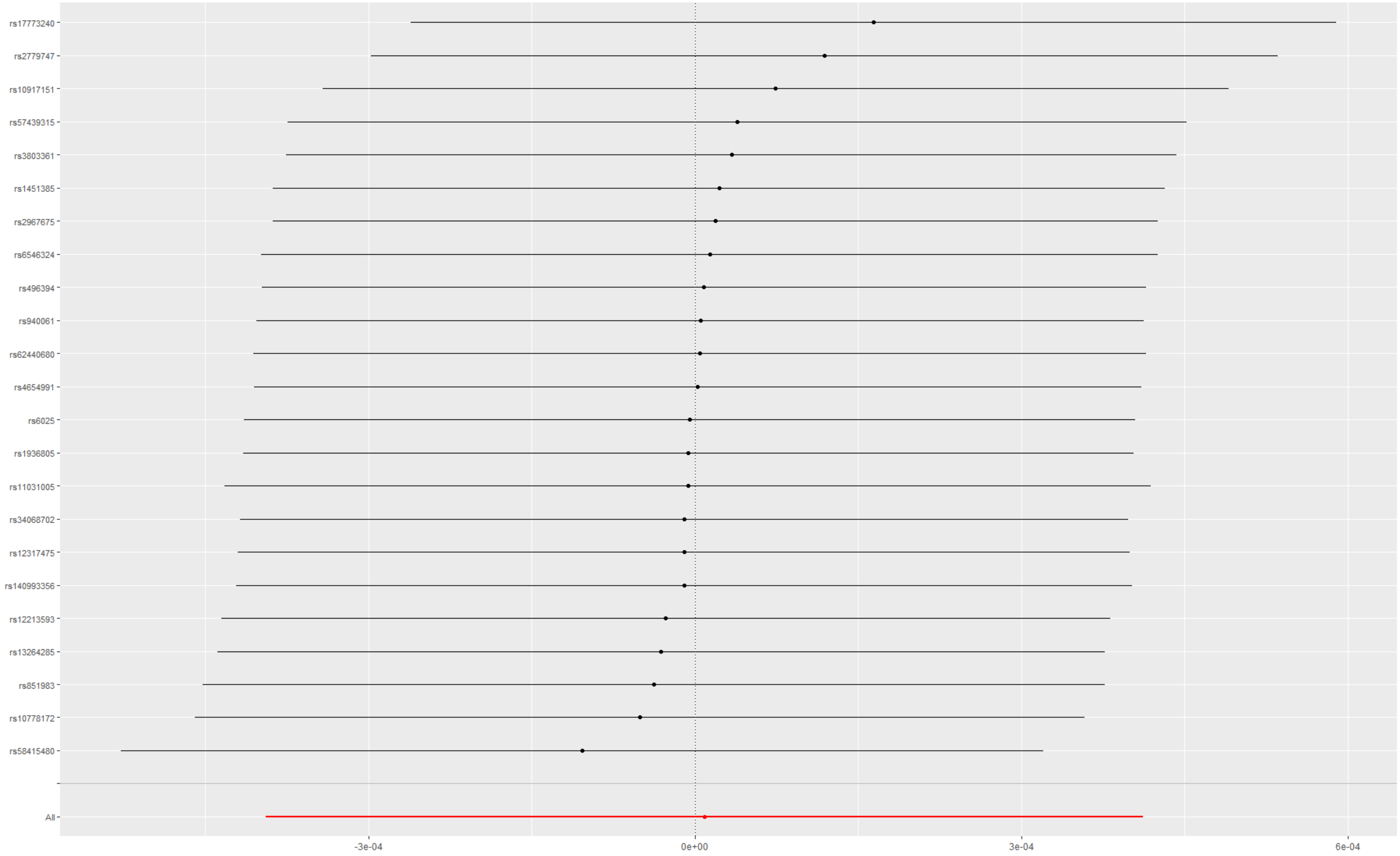

Supplementary Figure 35. Leave-one-out inverse-variance weighted mendelian randomization analyses of endometriosis on brain cancer

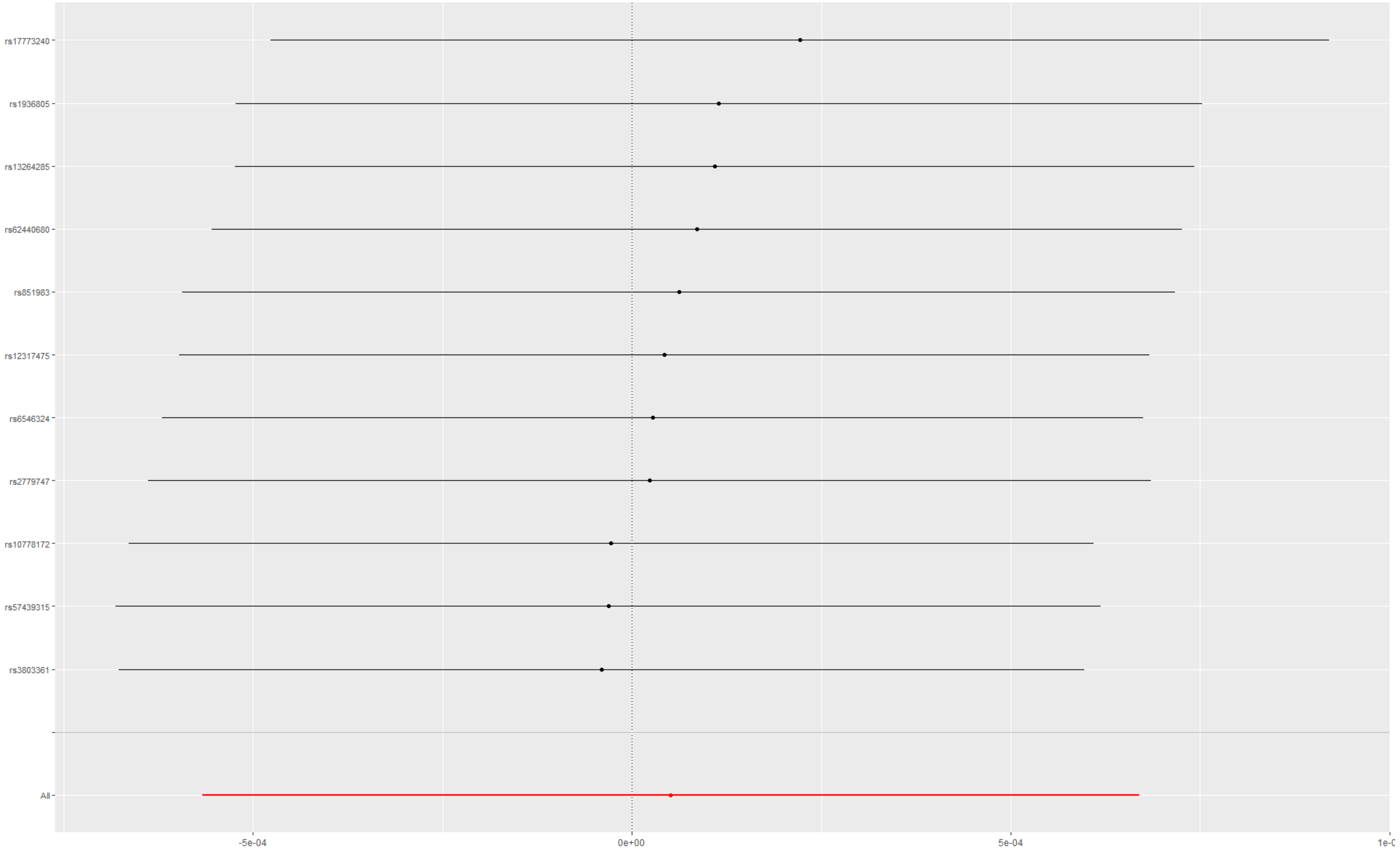

Supplementary Figure 36. Leave-one-out inverse-variance weighted mendelian randomization analyses of endometriosis on kidney cancer

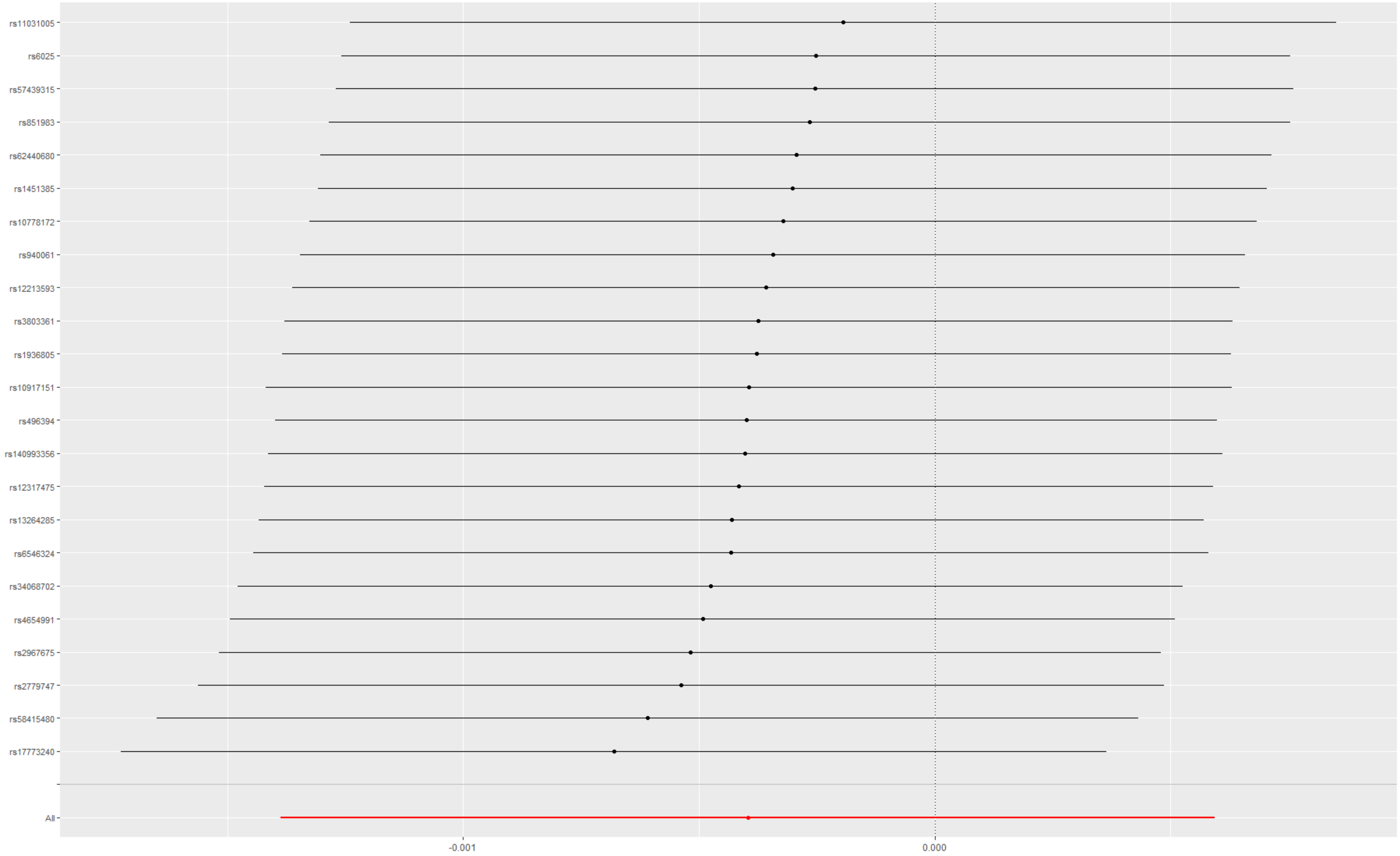

Supplementary Figure 37. Leave-one-out inverse-variance weighted mendelian randomization analyses of endometriosis on melanoma

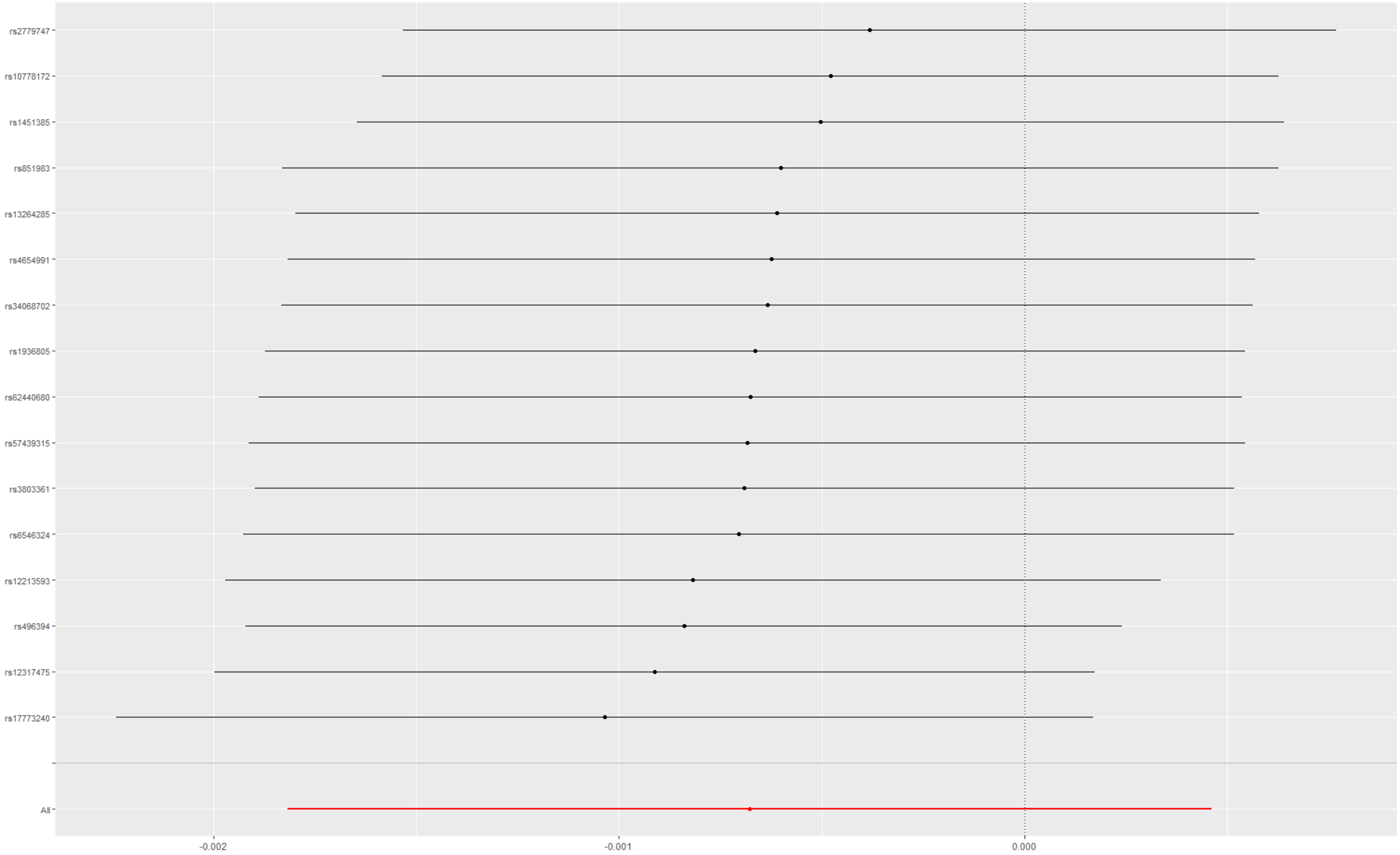

Supplementary Figure 38. Leave-one-out inverse-variance weighted mendelian randomization analyses of endometriosis on cervical cancer
